# Blending Gelators to Tune Gel Structure and Probe Anion-Induced Disassembly

**DOI:** 10.1002/chem.201303153

**Published:** 2013-12-02

**Authors:** Jonathan A Foster, Robert M Edkins, Gary J Cameron, Neil Colgin, Katharina Fucke, Sam Ridgeway, Andrew G Crawford, Todd B Marder, Andrew Beeby, Steven L Cobb, Jonathan W Steed

**Affiliations:** [a] Department of Chemistry, University of DurhamSouth Road, Durham, DH1 3LE (UK); [b] Institut für Anorganische Chemie, Julius-Maximilians-Universität Würzburg97074 Würzburg (Germany)

**Keywords:** blend, co-gels, fluorescence, orthogonal self-assembly, supramolecular chemistry

## Abstract

Blending different low molecular weight gelators (LMWGs) provides a convenient route to tune the properties of a gel and incorporate functionalities such as fluorescence. Blending a series of gelators having a common bis-urea motif, and functionalised with different amino acid-derived end-groups and differing length alkylene spacers is reported. Fluorescent gelators incorporating 1-and 2-pyrenyl moieties provide a probe of the mixed systems alongside structural and morphological data from powder diffraction and electron microscopy. Characterisation of the individual gelators reveals that although the expected α-urea tape motif is preserved, there is considerable variation in the gelation properties, molecular packing, fibre morphology and rheological behaviour. Mixing of the gelators revealed examples in which: 1) the gels formed separate, orthogonal networks maintaining their own packing and morphology, 2) the gels blended together into a single network, either adopting the packing and morphology of one gelator, or 3) a new structure not seen for either of the gelators individually was created. The strong binding of the urea functionalities to anions was exploited as a means of breaking down the gel structure, and the use of fluorescent gel blends provides new insights into anion-mediated gel dissolution.

## Introduction

There has been a great deal of recent interest in the use of multi-component supramolecular gels as a means of creating responsive materials with enhanced functionalities and thus of tuning the properties of the resulting gels.[1] A number of different types of multi-component gels can be distinguished depending on stoichiometry, and the nature and gelation propensity of the individual components. Fascinating results have been obtained by bringing together two or more components in a well-defined stoichiometry to create a gel-forming supermolecule, for example, mixtures of carboxylic acids and amines,[2] nucleobase analogues[3] or dendritic moieties.[4] Gel formation by metal-ion-complexing gelators is also a well explored example of such stoichiometric multi-component gels.[5] These systems are analogues to molecular co-crystals[6] and, hence, could be referred to as “co-gels”. However, the term co-gel has been used more broadly to cover non-stoichiometric mixtures of clays,[7] inorganic sol-gels, such as silica-titania,[8] or chemically modified gelatine type hydrogels.[9] The concept can be further extended to include composite gels incorporating nanoscopic materials, such as clay nanosheets,[10] nanoparticles,[11] carbon nanotubes[12] and graphene.[13]

Less well studied in supramolecular systems is the blending of non-stoichiometric mixtures of two components, which are each, individually, gelators. Mixing of LMWGs may lead to the formation of two independent, non-interacting networks which have been termed “multi-gelator” gels,[14] or mixtures of gel and crystals.[15] These self-sorting systems represent an example of orthogonal self-assembly and are analogues to other self-sorting systems, such as the amphiphile–gelator orthogonal co-assembly reported by van Esch.[16] On the other hand, two or more components can combine to give a single, sample-spanning gel network comprising gel fibres that contain more than one component.[17] These systems represent the gel analogues of solid solutions,[18] and are related to intimate blends of covalent polymers.[19] Such non-stoichiometric gelator “blends” offer the intriguing possibilities of: 1) smoothly and continuously tuning gel properties, and 2) introducing addressable luminescent or redox-active probes without significantly perturbing the overall gel structure. However, the hierarchical nature of gel formation means that even small differences between gelators may result in significant and surprising variation in gelator packing, fibre morphology or gel properties.

We now report the formation of both blended and phase-separated gels from non-stoichiometric mixtures of structurally related gelators (**1**–**7**) derived from amino acids. The seven gelators contain a common bis(urea) core, which is expected to drive gel formation through an α-tape hydrogen bonding interaction[20] to produce self-assembled fibrillar networks (SAFINs).[21] In principle, the common hydrogen bonding motif should tolerate the presence of different terminal amino acid ester substituents within the same fibrils. However, differences in higher order packing effects or spacer length may result in self-sorting and the formation of fibrils containing non-statistical mixtures of gelators or even independent networks. In this study, we contrast the gelling behaviour of the individual gelators with the effect of blending different gelators on the SAFINs formed. In particular, we make use of gelators **6** and **7** that incorporate fluorescent pyrenyl groups to provide an additional handle for studying gel blending and anion-induced disassembly in these mixed gelator systems.[22]

**Figure:**
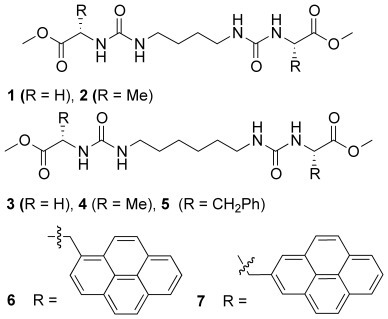


## Results and Discussion

**Figure 1:**
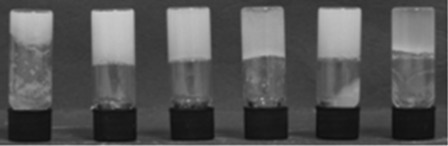
Gels of alanine-derived gelator 4 (1 % w/v) in (left to right) water, acetonitrile, THF, chloroform, ethyl acetate and toluene.

**Gel formation**: Bis(urea) gelators **1**–**5** can be prepared readily by treatment of the appropriate methyl ester protected amino acid with 1,4-diisocyanatobutane or 1,6-diisocyanatohexane. Compound **5** has been reported previously.[23] Samples containing 1 % (w/v) of gelator were heated to boiling point in sealed vials with a range of solvents. On cooling from a hot solution, the glycine derivatives **1** and **3** form gels only in toluene, with gels formed from the hexylene spacer **3** being notably stronger than the butylene analogues. In contrast, alanine derived gelator **4** forms robust gels in toluene, chloroform, ethyl acetate, THF, acetone and acetonitrile and partial gels in dichloromethane (DCM; examples shown in Figure 1). Gels are also produced in ethanol and water but are unstable, particularly the hydrogels, and rapidly break down to give a precipitate. The butylene analogue **2** behaves similarly to **4**; however, the gels are generally weaker and break down over time. The phenylalanine-derived compound **5** produces robust gels from toluene and ethyl acetate. Weaker gels also form in acetone and acetonitrile; however, gel formation is poorly reproducible with precipitates or partial gels often obtained using ostensibly the same procedure. As previously reported, compound **5** also forms robust gels in a number of aqueous solvent mixtures, namely 1:1 DMSO/water, 3:2 methanol/water and 1:4 THF/water.

**Figure 2:**
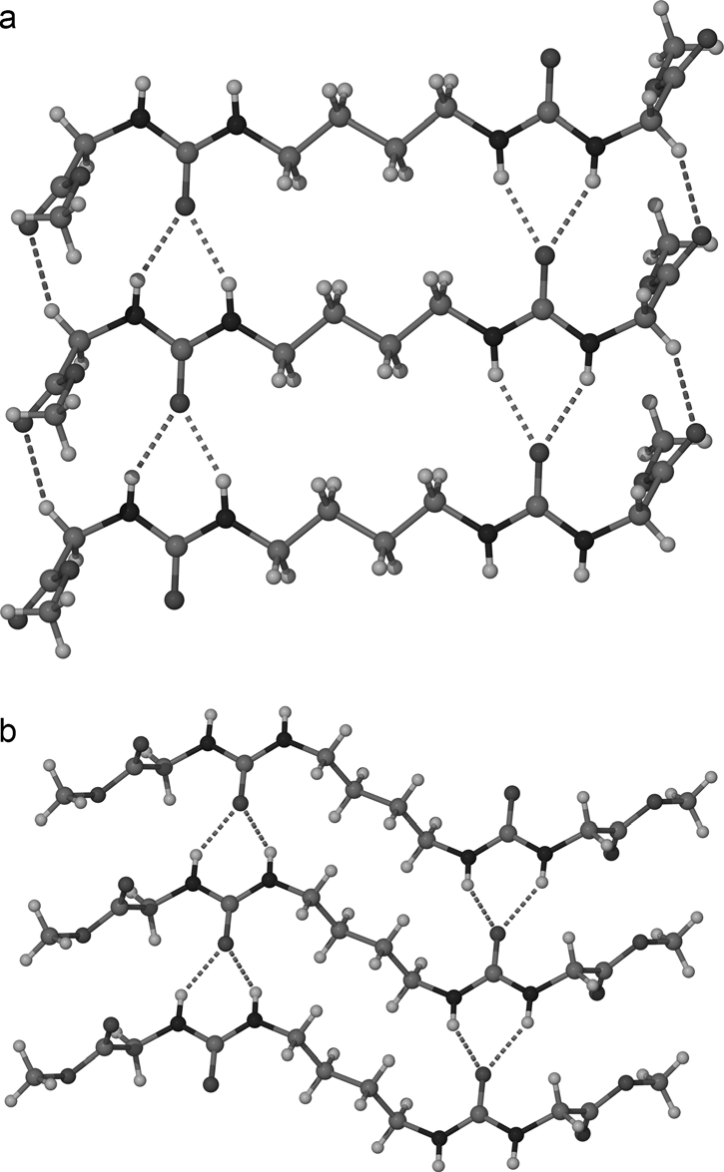
X-ray crystal structures of the conformational polymorphs of gelator 1: a) form A, closely related to the structures of 2–4, and b) form B.

**Gel structure**: Single crystals suitable for X-ray diffraction measurements were obtained from either methanol or water for compounds **1**–**4** and their structures solved. Two concomitant conformational polymorphs[24] of compound **1** crystallised from methanol solution: the majority of the crystals show plate-like morphology and were designated as form A, whereas an additional small amount of needle-shaped crystals were designated form B. The crystal packing arrangement in form A of **1** and in compounds **2**–**4** is isostructural to one another (although not isomorphous) and is based on an antiparallel double urea α-tape hydrogen bonded motif.20a,d, [25] The hydrogen bonding is aligned along the crystallographic [100] direction in each case, and their similar structure is exemplified by the near-constant length of the *a* axis (4.60–4.68 Å) of the primitive unit cells. The alkylene chains all adopt a parallel all-*trans* conformation and the amino acid ester substituents are located almost perpendicular to the long molecular axis, engaging in CH⋅⋅⋅O hydrogen bonding interactions[26] with adjacent molecules. The glycine derivatives **1** and **3** crystallise in centrosymmetric space groups, whereas the chirality of the l-alanine derivatives in **2** and **4** is evident in their adoption of the Sohnke space groups[27] *P*1 and *P*2_1_, respectively. The representative structure of **1** form A is shown in Figure 2 a.

In contrast to the other structures, form B of compound **1** has the two urea functional groups turned out of the plane of the all-*trans* butylene chain. Forms A and B are therefore conformational polymorphs. Conformational polymorphism of this type has been observed previously in butylene-spacer bis(urea) gels bearing phenyl ethyl substituents, and a key question is whether both or only one, and if so which, packing arrangement is involved in forming the gel fibres themselves.[28]

X-ray powder diffraction (XRPD) analysis was undertaken on a range of samples of gelators **1**–**5** prepared with four solvents (acetonitrile, acetone, ethyl acetate and toluene) representing a range of polarities and including both gel-forming media and solvents from which the compounds precipitate without gelation. Samples were prepared by heating 1 % (w/v) of the compound in a sealed vial until a clear solution was formed. The samples were then rapidly cooled in a water bath and briefly sonicated to ensure homogeneous gel formation, where applicable. Xerogels or precipitates were obtained from freshly cooled samples by rapidly removing the solvent under vacuum to minimise the likelihood of solid-form conversion during the drying process. A detailed description of the results is given in the Supporting Information.

At least two different crystalline forms (A and B) were identified for each compound (with some evidence for additional forms in a few cases). Only one polymorph is associated with gel formation for all of the hexylene spaced compounds studied, whereas in the butylene spaced compounds **1** and **2** both forms A and B can give rise to gels. In compounds **3** and **4**, the gelling form does not match that of the single crystal structure obtained, as shown by XRPD. It is therefore interesting to note that although gels can be formed by form A of butylene spaced compounds **1** and **2**, gels are not formed by the isostructural hexylene-spaced analogues. The gradual break down observed for toluene gels **2** and hydrogels of **4** appears to result from a polymorphic transition in which a metastable gelling form converts to a thermodynamically more stable crystalline form. Although there have been previous reports of polymorphism in LMWGs,[29] this role of polymorphism in explaining complex gelation behaviour is perhaps often overlooked.

Two different gels of **1** can be produced from toluene depending on sample preparation and show different morphologies, rheology and XRPD patterns matching the crystal structures of either form A or form B. Cooling of a hot toluene solution of **1** results in the formation of a weak sample-spanning gel, which was shown to have a structure matching that of **1** form A. However, heating of solid compound **1** (form A) in toluene also produces a stiff gelatinous material, which partially immobilises the solvent as an intermediate state before the compound fully dissolves. The XRPD pattern of this gelatinous material once dried indicates that it consists predominantly of form B, with small amounts of form A also present. DSC analysis of the solid form A xerogels of **1** from cooled toluene solutions gives a melting onset of 178 °C followed by recrystallisation and a second melting endotherm with onset 194 °C. In contrast, the form B xerogels isolated from hot toluene show only one melting endotherm at 196 °C. This suggests that form B is the most stable form at high temperature.

Overall, we suggest that the two solid forms A and B of each compound are based on a common urea α-tape packing motif, but with a different end group or spacer conformation (as observed in the single crystal structures of **1**). The B-type forms appear to have a greater propensity to form gels than the A-type, which are also far easier to characterise crystallographically.

**Figure 3:**
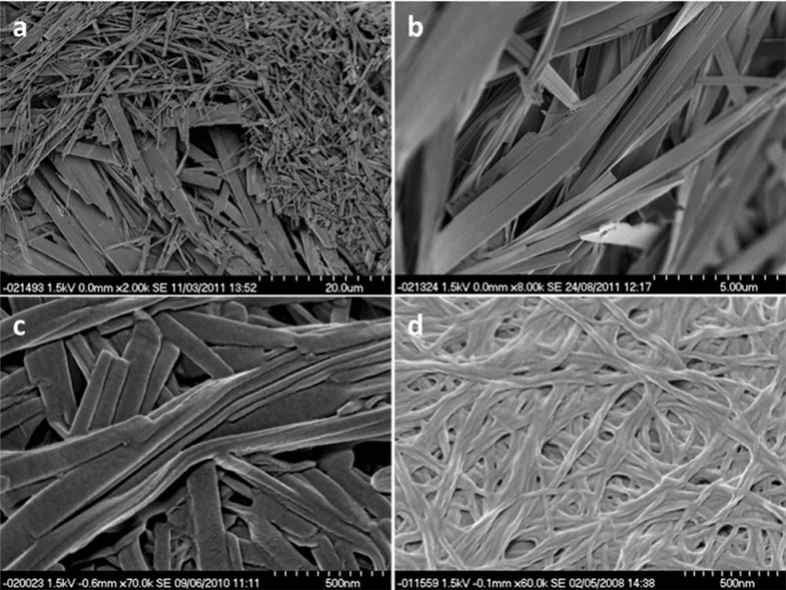
SEM images of xerogels formed from 1 % (w/v) of: a) 1 heated in toluene showing forms A (plates) and B (needles); scale bar: 20 µm; b) gels of 3 in toluene; scale bar: 5 µm; c) gels of 4 in THF; scale bar: 500 nm; d) gels of 5 in toluene; scale bar: 500 nm.

SEM analysis of the xerogels obtained from a variety of solvents showed that compounds **1**–**4** all form two dimensional, ribbon-like fibres. There is greater variation in the dimensions of the fibres within samples than between them, with the thickness of the ribbons typically 10–100 nm, the width between 100 nm and 5 μm and lengths from 100 nm to greater than 100 μm. The ribbons form physically entangled networks with separate fibres weaving and wrapping around each other and are often stacked on top of one another (Figure 3 c). The ribbons can also split along their length and branch into two separate fibres (Figure 3 b), which may provide a further means of cross-linking. The alanine gelators **2** and **4** typically exhibit a xerogel morphology comprising smaller, less linear fibres than other gelators, which corresponds to the more robust and more translucent gels observed. Two distinct morphologies are observed concomitantly for samples of **1** prepared from the gelatinous aggregates formed when **1** is heated in toluene; large plate-like ribbons, and smaller rod-like structures (Figure 3 a). Only the larger, plate-like ribbons are seen in samples prepared from toluene gels of **1**. XRPD data indicate that the different morphologies correspond to the two different polymorphs of **1** with the plate-like structures form A and the rod-like structures form B.

In contrast to the two-dimensional structures formed by the other gelators, phenylalanine derivative **5** forms randomly entangled networks of cylindrical fibres with strong preference of one direction of growth (Figure 3 d). Individual fibrils are microns in length with a relatively consistent diameter of approximately 50 nm. These fibrils appear to wrap around each other, sometimes in a helical manner, to form larger fibres. There is considerable variation in the aggregation behaviour of these fibres depending on the gelation solvent. The fibres from toluene are more randomly orientated than the acetonitrile and acetone samples in which the fibres tend to be bunched together into partially aligned clumps. The fibrils from the 1:4 THF/water mixture wrap around each other forming needle-shaped structures whereas the DMSO/water gel consists of thinner fibres composed of individual fibrils, which branch off and recombine with other fibres. This aligning and physical entanglement of fibrils and fibres appears to be the mechanism for cross-linking to give a sample-spanning network.

**Gel rheology**: Rheological measurements confirmed gel-like behaviour in 1 % (w/v) samples of **2**–**5** in toluene, and rheometric properties are summarised in Table 1. Initially, the elastic modulus (*G*′) of all samples is markedly greater than their viscous modulus (*G*′′). Application of increasing stress to the samples eventually results in the gel yielding, with *G*′′ becoming greater than *G*′. The general trend in gel strength and yield stress matches bench-top observations, with **4** and **5** forming strong robust gels and **2** and **3** more fragile gels.

**Table 1:** Summary of rheometric data for 1 % (w/v) toluene gels of 1–5.

	Compound					
	1form A	1form B	2	3	4	5
*G*′ [Pa]	7.6×10^2^	1.3×10^5^	5.2×10^3^	4.5×10^3^	4.6×10^4^	2.5×10^5^
*G*′′ [Pa]	8.9	4.6×10^3^	9.9×10^2^	3.4×10^2^	3.1×10^3^	2.1×10^4^
*G′*/*G′*′ [Pa]	8.6	27.7	5.3	13.3	15.2	12.2
Yield stress [Pa]	2	>300	20	25	126	>300

As well as the gels of **1** in toluene, the structure of which is assigned as form A by XRPD of the xerogel, the gel-like material formed upon heating a sample of **1** in toluene, which comprises predominantly form B, was also tested. The form B material gives strong gels with high *G*′ values, which failed to yield at an applied stress of 300 Pa. Form A gels, in contrast, were much weaker, readily breaking down to solutions with low applied stress (2 Pa).

**Gel blend co-assembly**: Given the structural similarity between gelators **1**–**5** we anticipated that it should be possible to use them to produce co-gel blends with properties tuneable by varying the gelator composition. The extent of polymorphism found across the series indicates that individual gelators are able to adopt a number of low-energy conformations while conserving the urea α-tape hydrogen bonding motif. However, small changes in substituent can lead to subtle differences in the way the molecules pack together, potentially resulting in differences in fibre morphology, growth patterns, connectivity and, hence, material properties. A key question is whether phase separation occurs such that the mixed gels comprise separate networks of each gelator, or whether the individual fibrils are comprised of both gelators as an intimate blend in an ordered, statistical or random arrangement.

A number of binary combinations of gelators **1**–**5** with a total of 1 % (w/v) of gelator in toluene solution were investigated by SEM and XRPD. All of the combinations formed gels at all of the ratios tested. On one hand, this is unremarkable in that the critical gelation concentration (CGC) of many of the gelators is well below 0.5 % (w/v). On the other hand, given the structural differences of the compounds, it is perhaps surprising that gel formation was not disrupted.

**Figure 4:**
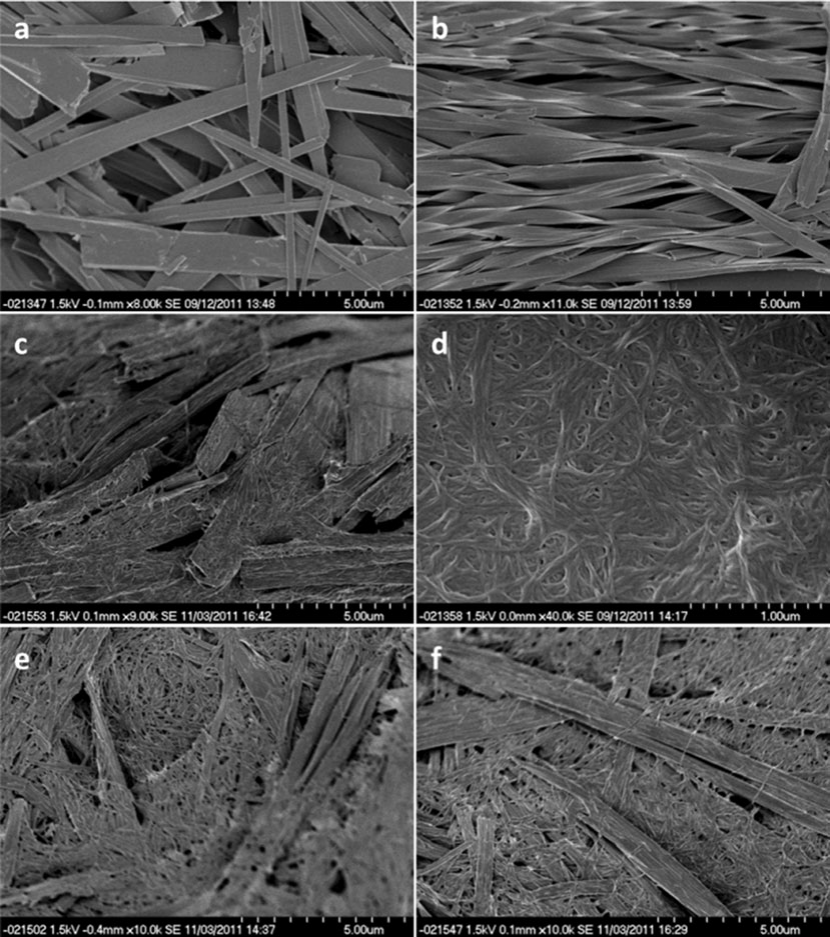
Mixed 1:1 toluene gels (1 %, w/v) interpreted as showing: a) ribbons of 1/2; b) helical co-gel fibres of 3/4; c) separate ribbons and cylindrical fibres of 3/5; d) cylindrical co-gel ribbons of 4/5; e) separate ribbons and cylindrical fibres of 1/5; f) separate ribbons and cylindrical fibres of 2/5. Scale bars a–c, e, f: 5 µm; d: 1 µm.

Figure 4 provides a summary of the morphology of xerogels formed from binary 1:1 mixtures of various gelators in toluene. Mixtures of butylene-spaced gelators **1** and **2** exhibit flat ribbon-like structures similar to those seen for the pure compounds (Figure 4 a). Similarly, mixtures of the hexylene-spaced gelators, **3** and **4**, also form two-dimensional ribbons similar to the parent compounds. However, these ribbons adopt a marked helical twist not observed for the achiral glycine-derived gelator **3** (Figure 4 b). This type of helical morphology is well known and often associated with chiral building blocks,[30] although the twisted morphology must be interpreted with caution since stress during crystallisation due to thermal fields (due to heat of crystallisation), mechanical fields (due to density differences) and compositional fields (due to concentration and impurities) can also result in these kinds of effect.[31] However, the fact that this helical morphology is not observed in the pure substances suggests that in the co-gel, the presence of the chiral L-alanine-derived gelators may exert a “sergeants and soldiers” type effect,[32] inducing a helical twist in the flat ribbon morphology of the achiral glycine-derived gelator molecules. Indeed, the induced twisting of fibres by chiral species has recently been harnessed as a sensor for detecting chiral compounds.[33]

SEM images of 1:1 mixtures of **3** and **5** clearly show separate fibres with the distinct ribbon-like and cylindrical morphologies characteristic of the parent compounds (Figure 4 c). In comparison, 1:1 mixtures of **4** and **5** show almost exclusively a cylindrical morphology (Figure 4 d), with only the occasional ribbon-like structures observed by SEM, in contrast to the dominant ribbon structure of pure **4**. These observations indicate that compound **4** interacts strongly with phenylalanine derivative **5** to produce blends, whereas **3** and **5** are sufficiently dissimilar that they form orthogonal networks, each with the morphology of the pure compounds.

Hexylene-spaced gelator **5** was also combined with butylene-spaced gelators **1** and **2** to assess the impact of spacer length on co-gel formation. As with its hexylene analogue (**3**), glycine derivative **1** forms separate ribbon-and fibre-like structures when combined with **5** (Figure 4 e). Separate ribbons and fibres were also clearly seen for mixtures of **5** with alanine-derived gelator **2** (Figure 4 f). It is interesting to note that with the same spacer length, mixtures of **4** and **5** form co-gels with a single morphology whereas with mismatched spacer lengths, **2** and **5** form orthogonal networks comprising separate fibres and ribbons. This is readily rationalised in terms of compounds with the same spacer length being able to form mixed bis-urea α-tapes more readily than those with different spacer lengths, hence favouring blending of the gelators into a single fibre morphology.

**Figure 5:**
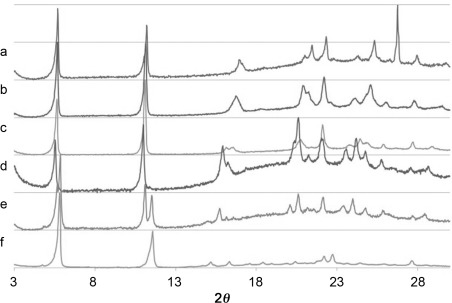
XRPD patterns corresponding to xerogels formed from toluene of glycine and alanine derivatives 1:2 in a ratio of: a) 1:0, b) 4:1, c) 3:2, d) 2:3, e) 1:4, f) 0:1.

Figure 5 shows the XRPD pattern for mixtures of the butylene-spacer glycine and alanine-derived **1** and **2** with the proportion of **1** decreasing in 20 % steps from top to bottom. The XRPD patterns for mixtures ranging from 20 to 60 % **2** in **1** closely resemble the pattern for the pure glycine-derived gelator **1** (form A) and indicate the formation of a single blended phase. Small shifts occur in a number of peaks as the proportion of the alanine derivative in the blend increases, most noticeably a shift in the (010) from 16.7 to around 15° 2*θ*. We interpret these data as resulting from gelator blends which adopt the crystal packing arrangement of compound **1** form A with incorporation of **2** resulting in an increase in the size of the unit cell in the *b* direction, but with no change in the *c*-axis length, as there is little change in the positions of the first two peaks, (001) and (002). The width of the *b* axis corresponds to the direction that the alanine methyl group would substitute for the glycine hydrogen atom in the structure. At a 1:4 ratio of a mixture of compounds **1** and **2**, an additional set of peaks that match those of **2** form B are observed in addition to those of the blend, indicating the formation of an additional, separate network of **2** at high concentrations of the gelator.

XRPD patterns for xerogels arising from the gelation of mixtures of **3** and **4** have a single set of peaks that does not match that of either pure **3** or **4** (see the Supporting Information). The positions of the peaks assigned to the new mixed phase vary significantly depending on the composition of the mixture while retaining the same basic pattern. The XRPD patterns of the **4**/**5** mixtures show a gradual transition in peak shape from well-resolved peaks matching compound **4** to broad peaks corresponding to the pattern of **5**. However, the pattern for the **2**:**3** mixture does not fit with this trend and shows well resolved peaks different from either parent compound. Overall, it is clear from the XRPD data that non-stoichiometric blends of closely related gelators with well-defined packing arrangements form readily.

**Fluorescent gels**: Pyrene derivatives have been used extensively as fluorophores in a variety of applications.[34] The high sensitivity of the vibronic fine structure of pyrene and its derivatives to changes in chemical environment (the Ham effect),[35] their long-lived fluorescence and the formation of fluorescent excimers through π–π interactions make pyrene derivatives particularly useful as fluorescent probes.[36] Extended aromatic ring systems, such as pyrene, have also been used to drive gel formation through π-stacking interactions.32d, [37] Pyrene is usually functionalised at the 1-, 3-, 6-or 8-positions where the maximum contributions of the HOMO lie, making it most amenable to electrophilic aromatic substitution. Substitution of pyrene at the 2-or 7-positions is synthetically challenging as a nodal plane in both the HOMO and LUMO passes through these positions. However, in 2-pyrenyl derivatives, substituents interact less strongly with the pyrene frontier orbitals than at other positions leading to more “pyrene-like” fluorescence.[38] The pyrenyl derivatives required to prepare **6** and **7** were synthesised using a versatile strategy for the preparation of artificial amino acids from an L-iodoalanine precursor formed in four steps from L-serine. Negishi coupling[39] is then used to connect the L-iodoalanine to either 1-or 2-bromopyrene (obtained either from aqueous hydrobromic acid and hydrogen peroxide,[40] or iridium-catalysed borylation,[41] respectively). In designing gelators **6** and **7**, we chose to couple artificial fluorescent amino acids bearing either 1-or 2-pyrenyl substituents to the same bis(urea) core of compounds **3**–**5** in order to produce fluorescent single component gelators. These compounds may also be used as probes of the gel formation process at low concentrations as part of a co-gel blend system. The structural similarity between phenylalanine derivative **5** and the two pyrenylalanine derivatives **6** and **7** is particularly close.

**Figure 6:**
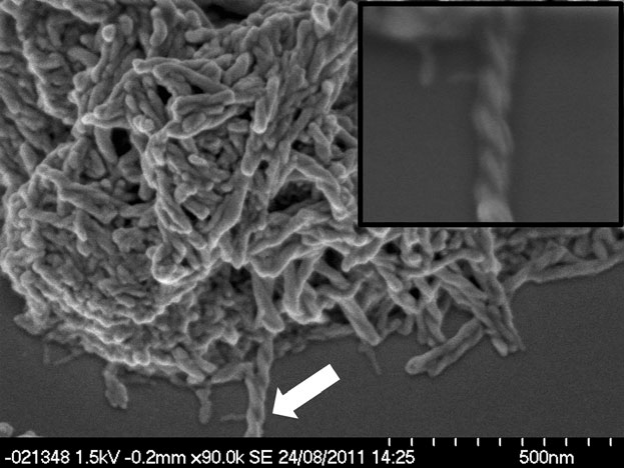
SEM images showing the morphology of 1 % (w/v) xerogels of 6 from methanol. Arrow indicates position of twisted fibre seen in the insert. Scale bar: 500 nm.

The 1-pyrenylalanine derived gelator **6** gels a wide range of solvents across the polarity spectrum at 1 % (w/v). Gels in acetonitrile, ethanol, acetone, THF, DCM, chloroform and toluene are robust and proved stable over a period of several weeks. Gels of methanol and ethyl acetate are only weak, partial gels. SEM revealed a morphology consisting of an entangled network of fine, flexible fibres approximately 50 nm in width (Figure 6). The fibres take the form of left-handed helices—an effect that may arise from the chirality of the molecules.20c, [30] The morphology is consistent across the range of solvents investigated. The 2-pyrenylalanine derivative **7** also forms gels across a range of solvents at 1 % (w/v) particularly acetonitrile, ethanol, methanol, THF and chloroform. The compound was found to be generally poorly soluble and there were difficulties dissolving it, particularly in low boiling point solvents, such as acetone, DCM and ethyl acetate. Attempts to form gels of **7** in toluene were unsuccessful and resulted in a precipitate. Electron microscopy revealed fine helical cylinders in xerogels of **7** formed from chloroform and methanol, similar to those observed in gels of **6**. Fine fibres are also identifiable in SEM images of xerogels of **7** from acetonitrile and the precipitate formed in toluene; however, they have a flatter, less well-defined shape. It is interesting to note that a small difference in the orientation of the pyrenyl group relative to the backbone between the two isomers translates into a difference in gelation behaviour and fibre morphology.

**Figure 7:**
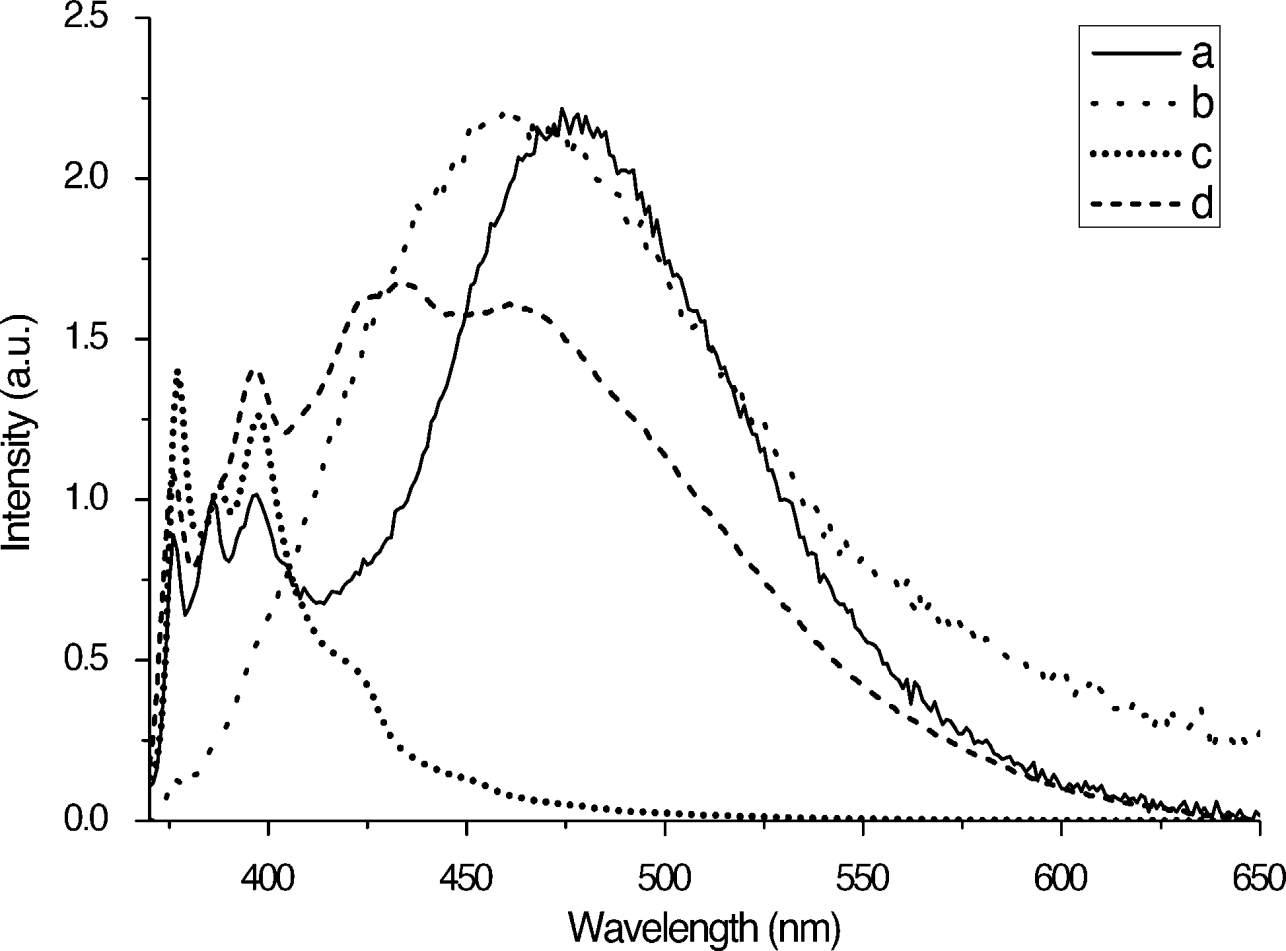
Emission spectra of 7 (*λ*_ex_=345 nm): a) in dilute toluene solution (1×10^−7^ mol dm^−3^); b) as a fibrous solid; c) co-gel blend of 5 and 7 (9:1); and d) after treatment of the 5:7 blend with 10 equivalents of NBu_4_OAc. The spectra are normalised at 386 nm apart from (b), which is scaled to match the intensity of the excimer peak of (a).

The emission spectrum of **7** in dilute toluene solution exhibits bands assigned to pyrenyl monomer emission as well as a broad, featureless excimer band with a maximum around 476 nm (Figure 7 a; see the Supporting Information for analogous spectra of **6**). This excimer band is absent in dilute toluene solutions of the precursor BOC-protected pyrenyl amino-acid methyl ester (used as a control; see the Supporting Information). The occurrence of the excimer band is thought to be due to intramolecular interactions between the pyrenyl-moieties at either end of the compound.[36] The hexamethylene spacer tethers the two pyrenyl functionalities together and is flexible enough for the pyrenyl groups to interact mutually resulting in excimer formation. The same features are observed for **6**, but with weaker excimer emission relative to monomer emission. This is thought to be due to the longer-lived excited state of the 2-pyrenylalanine in **7** allowing more time for excimer formation to take place.[38] Geometrical considerations may also play a role with the more symmetric 2-pyrenyl groups achieving better overlap than the 1-pyrenyl analogues.

Compound **6** forms robust gels in toluene at 1 % (w/v) whereas **7** forms fibrous precipitates. The emission spectra of both samples are dominated by strong excimer bands at about 466 nm, blue-shifted compared to the intramolecular excimer emission seen in dilute solution (e.g., 476 nm for **7**, a shift of 450 cm^−1^). As fibre formation occurs in both systems, the excimer bands are thought to correspond to intermolecular excimer emission resulting from interactions between adjacent pyrene groups within urea hydrogen bonded stacks. The intensity of the excimer emission band is particularly pronounced for **7**, which exhibits no trace of monomer emission. Gel formation by other pyrene derivatives has previously been found to quench[42] as well as induce excimer formation.[43] In both cases reported here, supramolecular polymerisation markedly enhances the relative intensity of the excimer band.

Gelation experiments were undertaken with fluorescent gelators **6** and **7** in mixtures with the glycine-, alanine-and phenylalanine-derived gelators **3**, **4** and **5**. At low concentrations the fluorescent gelators have the potential to act as a probe of gel formation and dissolution in their non-fluorescent analogues, whereas dilution within a structurally related gel may give insights into the origins of the photophysical processes occurring in gels of **6** and **7** themselves. Moreover, perturbation of the photophysical properties of the pyrene derivatives provides direct evidence for the formation of an intimately mixed, single-phase co-gel network rather than the orthogonal self-assembly of two separate networks.[16]

Gel blends were formed in toluene with 1 % (w/v) of mixed gelators comprising 10 % by mass (1:9 ratio) of gelators **6** or **7**, respectively, with either **3**, **4** or **5**. The samples were heated and sonicated until fully dissolved with gels forming upon cooling to room temperature. The pyrene derivatives proved difficult to dissolve completely and a number of heating and cooling cycles were undertaken to ensure complete dissolution and homogenisation occurred.

The emission spectra show that excimer emission is almost completely absent in the mixed gels compared to solution (Figure 7 c). This is thought to arise due to random incorporation of the pyrenyl gelators into bis(urea) tapes of the co-gelator. The X-ray crystallographic studies show that molecules in this series adopt an extended conformation of the alkylene spacers as part of a bis(urea) hydrogen bonded stack (see above). This conformation would prevent the pyrenyl molecule folding over to produce intramolecular excimers as is suggested to be the case in solution. Pyrenylalanine-derived gelator molecules that happen to be adjacent to one another within an individual urea stack may still be able to form intermolecular excimers; however, as the intensity of the excimer band is dramatically reduced, a high degree of blending is suggested to occur such that isolated fluorescent gelator molecules occur within a hydrogen bonded stack comprising predominantly non-fluorescent analogues. The excimer maximum at 463 nm in the 1:9 composition of **7** and **3** mixed gels is similar in energy to the intermolecular excimer observed for the fibrous precipitate formed from the 1 % (w/v) sample of **7** from toluene (466 nm). This suggests that the emission arises from intermolecular rather than intramolecular excimer formation. Overlap with the monomer emission makes identifying the maximum for the excimer band of **6**/**3** difficult, although the band appears substantially blue shifted relative to the intramolecular excimer of the solution sample. Decreasing the concentration of **6** relative to the phenylalanine analogue **5** in the mixed gel results in a decrease in the intensity of the excimer band (see the Supporting Information). This provides further evidence that the excimer band is due to intermolecular interactions between pyrenyl groups, with a lower probability of two pyrenyl moieties being adjacent in the fibres at lower concentrations. Reduction of the excimer band is most pronounced in mixed gels of **5** and **7** indicating that blending is most effective in this system. This is attributed to structural similarities between the two gelators allowing efficient mixing of the gelators in fibres. The emission spectra of the mixed gels did not vary substantially on storing the gels for a period of about one week. Some variation in excimer intensity was observed depending on whether the mixed sols were cooled quickly or slowly in order to induce gelation. Slightly higher excimer intensity was observed on rapid cooling of **6**/**3** or **7**/**3** suggesting more phase separation. However, fast cooling of **6**/**4** or **7**/**4** consistently gave the opposite effect, and no differences were observed with either **6**/**5** or **7**/**5**.

**Figure 8:**
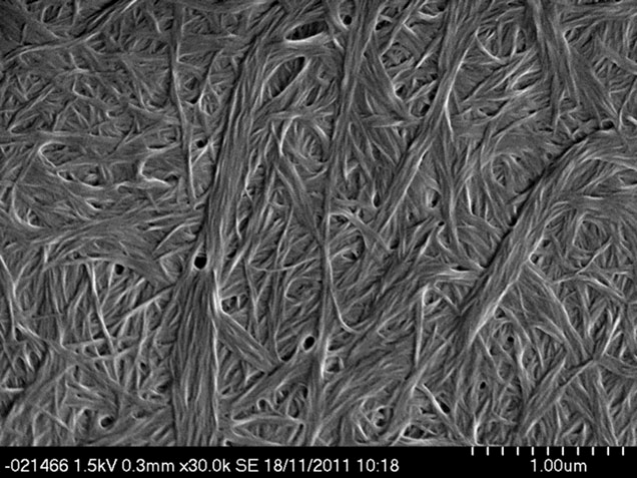
SEM images of xerogels of 6:5 formed from a total of 1 % (w/v) mixed gels in toluene with a ratio of 1:9 by mass. Scale bar: 1 µm.

SEM was used to image the morphology of xerogels formed from 1:9 mixed toluene gels of **6** or **7** with **3**, **4** and **5**. SEM images of the 1:9 mixed gels of **6** and the phenylalanine-derived gelator **5** showed a homogeneous sample comprising fine cylindrical fibres, similar to the xerogels of both pure compounds (Figure 8). The same morphology is also observed for 1:9 mixed gels of **7** and **5** with no evidence of the irregular ribbons observed in the toluene precipitates of pure **7**. Both pyrenylalanine mixed gels with **5** showed broad, poorly defined XRPD patterns with high similarity to each other and to those of the pure compounds **5**, **6** and **7** (see the Supporting Information). Overall, the data suggest a single blend phase with the fluorescent gelators incorporated randomly into the bis(urea) stack in the phenylalanine gel.

Mixed gels of alanine derivative **4** with either pyrenyl analogues show evidence for the formation of some fibres similar in morphology to the large ribbons formed by pure **4**. However, the sample is comprised of predominantly much smaller fibres similar in size to those of the pyrenylalanine gels (see the Supporting Information). The samples are inhomogeneous, with domains dominated by different morphologies. In a sample prepared using less than 0.1 mg mL^−1^ **7** in 1 % (w/v) gels of **4** in toluene (i.e., a 1:99 ratio of **7**/**4**), the majority of the sample consisted of ribbons; however, large regions of fibres were also observed. This indicates that even very small amounts of pyrenyl additives can have a significant effect on the morphology of gels of **4**. XRPD analysis of the mixed gels of **4** revealed a pattern very similar to that of the xerogels of **4**; hence, although dramatically different fibre morphology is seen, the underlying packing still resembles that of the majority-based alanine gelator. Marked broadening of some peaks (particularly 5.2° 2*θ* and 10.0° 2*θ*; see the Supporting Information) may arise from accommodation of the bulky pyrenyl groups.

**Figure 9:**
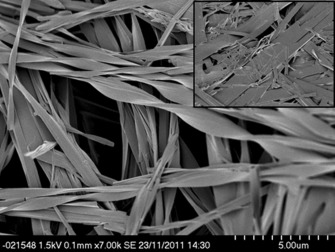
SEM image of helically twisted xerogels of 6:3 formed from a total of 1 % (w/v) mixed gels in toluene with a ratio of 1:9 by mass. Insert: analogous flat ribbons of pure 6 xerogels. Scale bar: 5 µm.

SEM images of mixed gels of the pyrenyl alanine gelators **6** and **7** with glycine derivative **3** (1:9 ratio) indicate the simultaneous formation of three different materials assigned on the basis of their morphology to xerogels of pure **3** (straight ribbons), pure **6** xerogels (fine cylindrical fibres), and a mixed blended phase comprising ribbons similar to those observed for pure **3** but with a clear left-handed twist (Figure 9). Compound **3** is achiral and, as this type of microscale helical twist may be associated with chiral molecules, it is possible that the twisting is induced by the incorporation of the chiral L*-*pyrenylalanine-derived gelators in a “sergeants and soldiers effect”, analogous to the effect observed in the mixed gels of **3** and **4**. In the mixed gels of **7**/**3**, the majority of the ribbons appear to be twisted implying less phase separation than with **6**. Compound **3** has two polymorphic forms: the gelling form B, which is produced in toluene, and non-gelling form A, which is observed from other solvents including acetonitrile. The XRPD patterns of gel blends **3**/**6** and **3**/**7** mixtures of separate gels most closely resemble the non-gelling form A of **3** obtained from acetonitrile. However, there is significant amorphous content and the observed Bragg peaks may reflect the presence of pure **3** in the sample.

**Anion influence**: The hydrogen bonding functionality of ureas allows them to interact strongly with anions, and thus ureas have been utilised in a variety of anion binding sensors, for example.[44] The addition of anions can be used to disrupt the urea–urea interactions in gels, weakening and ultimately preventing gel formation. Controlled addition of anions to gels has therefore received considerable interest as a means of tuning gel rheometry.22a,b, [45] We sought to influence the assembly and properties of the gel blends reported herein using anion complexation, resulting in highly controllable and addressable, ternary systems. The acetate anion was selected for investigation as it has previously been shown to bind strongly to urea functionalities due to its high basicity. Tetrafluoroborate was selected as a reference anion as it only weakly interacts with ureas.20c The anions were used as tetra-*n*-butyl ammonium (TBA) salts due to the weakly coordinating properties of this cation and high solubility in organic solvents.[28,46]

In order to quantify the interaction between the different gelators and anions, solution-based NMR spectroscopic titration studies were undertaken in [D_6_]DMSO due to reasonably high solubility of the gelator, even though the solvent shows competitive hydrogen bonding to the urea moieties. Anion binding constants are given in Table 2. The anion binding titrations indicate that weak 1:1 binding is dominant with additional contributions from 1:2 gelator/anion complexes, consistent with the widely separated disposition of the two urea functionalities. The binding constants are broadly comparable between the different compounds, with **3** showing the strongest binding and **4** the weakest. Analogous titrations with BF_4_^−^ showed no chemical shift change in the urea protons; hence, we conclude that binding of the control anion BF_4_^−^ is very weak under these conditions.

**Table 2:** Anion binding constants determined by ^1^H NMR spectroscopic titrations in [D_6_]DMSO for representative bis(urea) gelators.

Compound	log *β*_11_	log *β*_12_
**3**	1.96(2)	2.91(2)
**4**	1.30(6)	2.53(4)
**5**	1.77(4)	2.61(5)

The effect of anions on the fluorescence of solutions of **6** and **7** were investigated in DMF due to the narrow absorption window of DMSO. Solutions of TBA-acetate or TBA-tetrafluoroborate in DMF (1.3×10^−4^ mol dm^−3^) were gradually added to a solution of gelators **6** or **7** in DMF (1.3×10^−5^ mol dm^−3^) and the emission spectra were recorded. The solution phase emission spectra of both compounds in DMF show the presence of both pyrene monomer and excimer emission with the intensity of the excimer emission markedly enhanced relative to the analogous toluene spectra, perhaps due to greater pyrene–pyrene interaction in DMF relative to competition with toluene. Addition of TBA-acetate to both **6** and **7** results in a drop in the relative intensity of the excimer band (see the Supporting Information). The decrease in the intensity of the excimer emission is thought to be due to conformational changes in the gelator upon binding to the anions preventing the pyrene groups associating to form an intramolecular excimer. Due to weak binding in a competitive solvent, such as DMF, and the very low concentration of gelator, a large excess of anion is required to bring about this change (2500 equiv). Dilution of the final samples with DMF resulted in an increase in the relative excimer emission intensity, which is attributed to the reduced proportion of bound bis(urea) due to the decrease in anion concentration. Addition of non-binding BF_4_^−^ results in no change in the fluorescence emission over the same concentration range. The pyrenyl derivatives **6** and **7** are thus both potentially effective ratiometric sensors for acetate.

Varying equivalents of TBA-acetate were added to 1 % (w/v) of gels of **5** in 1:9 acetonitrile/toluene mixtures in a concentric cylinder couette apparatus. In the absence of anions, strong gel-like behaviour was seen with *G*′ values an order of magnitude higher than *G*′′, an effect which persists even at high oscillatory stresses of 300 Pa. Gels formed in the presence of increasing equivalents of TBA-acetate showed a lower value of *G*′ and a smaller difference in magnitude between *G*′ and *G*′′. The stress with which the gels broke down to give liquid-like behaviour, the yield stress, also decreased with increasing concentrations of anion. With 1.5 equivalents of TBA-acetate, the gel fails to form and the viscous modulus *G*′′ becomes greater than the elastic modulus *G*′. This acetate modulation of the rheological properties of bis(urea) gels is consistent with previous behaviour.[28,47] Interestingly, *G*′ is slightly higher in the 1:9 acetonitrile/toluene gel than for the pure toluene gel (250 000 Pa) of **5** when measured under the same conditions. The same trends are observed for 1 % (w/v) **5** in ethyl acetate with only 0.8 equivalents of acetate required to prevent gel formation, suggesting stronger acetate binding to the gelator in ethyl acetate than the toluene/acetonitrile mixture. The temperature at which gels of **3** and **4** form in toluene and ethyl acetate is greater than the boiling point of the solvents meaning it was not possible to transfer a sol from a sealed vial into the rheometer cup. Studies were therefore undertaken for **4** in chloroform, in which the compound is generally more soluble and gel formation slower and weaker. These observations were reflected in the rheometry, with *G*′ values an order of magnitude lower than for the phenylalanine gels. The presence of increasing concentrations of anions resulted in the same decrease in *G*′ and yield stress observed for gels of **5**. As **3** only forms gels in toluene, it was not possible to undertake studies on this compound.

We sought to exploit the fluorescent properties of **6** and **7** included within a co-gel blend of gelators **3**, **4** and **5** to probe the influence of anions on the gel structure at a molecular level. Studies were undertaken in toluene, because all gelators studied form strong gels in this solvent. Low concentrations of the L-pyrenylalanine gelators were used in order to minimise intermolecular excimer formation and simplify interpretation of the resulting spectra. Due to the low solubility of gelators **6** and **7** in toluene, saturated solutions were formed by suspending 1 mg of gelator in 10 mL of toluene and then filtering off any undissolved material. Co-gel blends were prepared by adding saturated solutions of **6** or **7** to **3**, **4** or **5** (1 %, w/v) followed by repeated heating and sonication cycles to ensure homogeneity. The tetrabutylammonium anion salts are poorly soluble in toluene; thus, acetonitrile was used as a co-solvent. TBA-acetate or TBA-BF_4_ was added as a solution in acetonitrile. Samples were dissolved by heating following the addition of anion solution and the gels were allowed to reform by cooling before a new measurement was taken.

Control experiments using dilute solutions of **6** and **7** in toluene without the presence of the co-gelator showed that the addition of acetate results in a decrease in the intensity of the excimer band (*λ*_max_=470 nm), consistent with the behaviour observed in DMF solution and is thought to be due to conformational changes upon binding of the gelator to the anion, inhibiting intramolecular excimer formation. The loss of excimer emission is more pronounced in toluene than in DMF, with only two equivalents of anion required to extinguish excimer emission. No loss of excimer band intensity is observed with the addition of the tetrafluoroborate, consistent with the weaker binding of the gelators to this anion. Indeed, a small increase in the intensity of the excimer band occurs accompanied by a shift in *λ*_max_ from 457 to 471 nm (650 cm^−1^), presumably due to the change in solvent environment.

**Figure 10:**
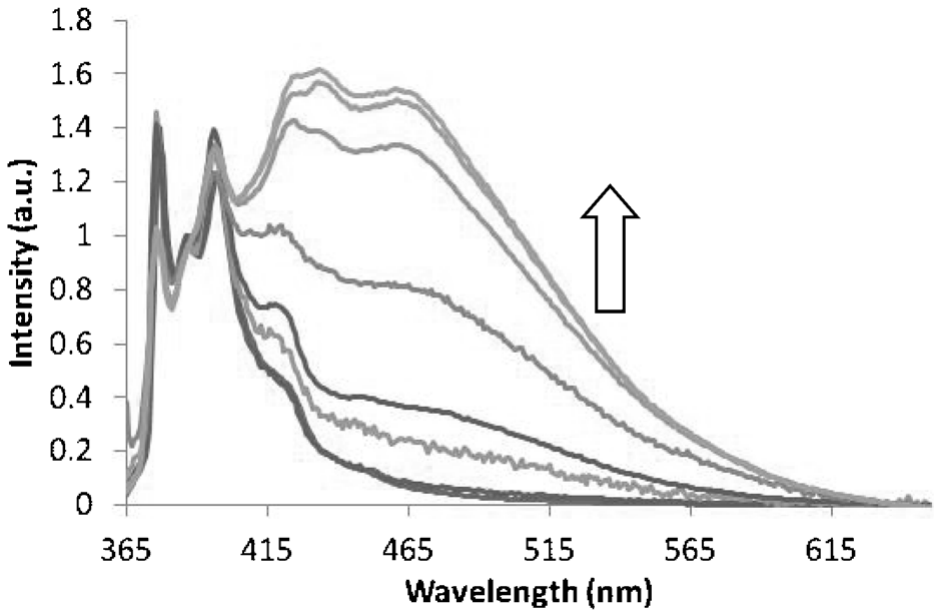
Emission spectra (*λ*_ex_=345 nm) showing the addition of tetrabutylammonium acetate solution to 1 % (w/v) 1:9 mixed gels of 7 and 5. Number of equivalents: 0, 0.5, 1, 2, 5, 10, 15 and 20. Spectra normalised to 386 nm.

Only pyrenyl monomer emission is observed in the mixed gels of **3**, **4** or **5** with **6** or **7** before the addition of anions (Figure 10 and the Supporting Information). This is consistent with the random incorporation of **6** and **7** in an isolated fashion into blend gel fibres preventing intermolecular excimer formation. The extended conformation of the molecules in the fibre also prevents intramolecular excimer formation. The addition of increasing amounts of TBA-acetate results in visible weakening of the gels after addition of about one equivalent of acetate anion with breakdown after approximately 1.5 equivalents, accompanied by a gradual increase in the intensity of the excimer emission bands. By inhibiting fibre formation, we postulate that the anions release the pyrenylalanine gelators to undergo intramolecular excimer formation in solution, as observed for the pure compounds. Although in solutions of the pure compounds **6** and **7** anion binding quenches excimer emission, in the mixed gel system there is a large excess of the non-fluorescent gelators (**3**, **4** or **5**) that can bind and effectively sequester the acetate, limiting the amount of acetate available to bind to, and, hence, quench the released pyrenyl gelators.

Upon addition of less than one equivalent of acetate there is relatively little change in the appearance of the excimer band. Upon addition of 1 to 2 equivalents of acetate the intensity of the excimer emission increases substantially in all samples, corresponding to the breakdown of the gels, which occurs around 1.5 equivalents of TBA-acetate for all of the mixed gels. It is interesting that excimer emission continues to increase with further addition of anions, long after the gel has broken down. This indicates that acetate continues to disrupt aggregation, which is therefore still taking place even after the destruction of the three-dimensional gel structure. Much greater increase in the intensity of the excimer emission are observed for the mixed gels containing **7** compared to those with **6**, consistent with the longer-lived excited state of this isomer.[38] The increase in excimer emission is more pronounced in mixed gels of **5** compared to **3** or **4** indicating greater dissolution of the gels of **5** by acetate. In some cases, for example, the 1:9 gel of **4**/**7**, the increase in excimer emission levels off at higher concentration of acetate, presumably as a result of complete disaggregation and, hence, full dissolution of the pyrenyl gelators.

An increase in the intensity of the excimer band is also seen with the addition of TBA-tetrafluoroborate solution, particularly at higher concentrations at which weakening of the gels is also seen. The effect is most pronounced for co-gels of glycine gelator **3**, which does not form gels with acetonitrile so may be broken down by the addition of the co-solvent. In the case of mixed gels of **3**/**6**, excimer emission is greater with the addition of TBA-tetrafluoroborate than TBA-acetate. This is presumably due to binding of free **6** to acetate, but not BF_4_^−^, reducing excimer emission in the sample but not the reference. Gelators **4** and **5** both gel acetonitrile and the increase in excimer emission is considerably larger with the addition of acetate than tetrafluoroborate solution.

## Conclusions

Amino acid derived bis(ureas) are effective organogelators of a range of solvents and form gels by means of the well-known urea α-tape hydrogen bonding motif. The observation of more than one polymorphic modification suggests that the basic double α-tape packing can adapt in different ways to the nature of the peripheral groups with some arrangements exhibiting a greater propensity to form gels than others. The different polymorphs of **1** resulted in gels with different morphology and rheology, whereas transformation to a more stable polymorph explains the degradation of aqueous gels of **4**.

Clear differences in the gel fibre morphology provide a straight forward means to distinguish between gel blends and orthogonal networks. In some cases, the packing arrangement across the series is sufficiently conserved to tolerate substitution of one member of the series for another to give a single, homogeneous, non-stoichiometric gel blend with tuned properties and morphology. Compound **4** interacts strongly with phenyl alanine derivative **5** to produce a blend, whereas **3** and **5** are sufficiently dissimilar that they form orthogonal networks, each with the morphology of the pure compounds. In the case of achiral **3**, introduction of a chiral additive apparently results in a “sergeants and soldiers” effect and induces a helical twist to the gel ribbons. Compounds with the same spacer length are able to form mixed bis(urea) α-tapes more readily than those with different spacer lengths, hence favouring co-fibre blend formation. Blends of **1** and **2** were found to adopt a single packing motif, which could be identified as corresponding to that of compound **1** (form A), whereas other blends showed new packing motifs not seen for either pure gelator.

In general, the synthetic 1-and 2-pyrenylalanine gelators (**6** and **7**) behave similarly to the other amino acid derivatives in the series. In free solution, the two pyrenyl ends of gelators **6** and **7** are able to interact mutually in their excited state to form an intramolecular excimer, with stronger excimer emission observed for the symmetric 2-pyrenyl derivative. In mixed gel blends with compounds **3**, **4** and **5**, intramolecular excimer formation is prevented as the pyrenyl gelators become incorporated into bis-urea tapes. As the proportion of pyrenylalanine gelator is increased, a new intermolecular excimer band forms due to association between adjacent pyrenylalanine gelators. Compounds **6** and **7** incorporate well into the gel of **5**, resulting in quenching of the excimer emission and no observable differences in gel morphology or XRPD patterns compared to pure gels of **5**. Partial phase separation is observed in mixtures of **6** and **7** with compounds **3** and **4**, although a blended phase predominates.

Formation of pure and mixed gels is markedly disrupted by addition of acetate anions, consistent with binding of acetate to the urea functionalities. Binding of acetate to **6** and **7** in solution results in quenching of the intramolecular excimer emission, thought to result from conformational changes in the gelators upon binding to the anions preventing association of the pyrenyl end groups. Addition of acetate to gel blends containing fluorescent gelators **6** and **7** breaks down the gels, releasing the pyrenylalanine gelators to undergo intramolecular excimer emission whereas the acetate is sequestered by the excess non-fluorescent gelator. Excimer emission intensity continues to grow with the addition of excess acetate indicating that the aggregation of the gelators continues to be disrupted at anion concentrations well above those required to prevent macroscopic gel formation.

Blending closely related gelators provides a ready means for tuning the properties and morphologies of gels as well as a way to gain insights into gel formation and dissolution. However, this work highlights the sensitivity of the gel state to differences in gelator resulting in orthogonal network formation or novel structures and morphologies.

## Experimental Section

**Methyl 2-[4-[(2-methoxy-2-oxo-ethyl)carbamoylamino]butylcarbamoylamino]acetate (1)**: Glycine methyl ester hydrochloride (1.00 g, 7.96 mmol) was suspended in chloroform (30 mL) and an excess of triethylamine was added, whereupon the suspension dissolved. 1,4-Diisocyanatobutane (0.56 g, 3.98 mmol) in chloroform (20 mL) was added dropwise and the reaction was then heated at 70 °C for 18 h, during which period a white suspension formed. The suspension was filtered leaving a white solid that was then washed with water and dried in a drying pistol. Compound **1** was isolated as a white powder (0.96 g, 3.02 mmol, 76 %): ^1^H NMR (700 MHz, [D_6_]DMSO): *δ*=6.13–6.08 (4 H, m, NH), 3.73 (4 H, d, *J*=6.0 Hz, COC*H*_2_), 3.58 (6 H, s, O*C*H_3_), 2.95–2.93 (4 H, m, NHC*H*_2_), 1.32–1.30 ppm (4 H, m, NHCH_2_C*H*_2_); ^13^C{^1^H} NMR (176 MHz, [D_6_]DMSO): *δ*=172.1 (*C*OO), 158.3 (N*C*O), 51.9 (OC*H*_3_), 41.8 (CO*C*H_2_), 40.3–39.6 (NH*C*H_2_ under residual solvent peak), 27.9 ppm (NHCH_2_*C*H_2_); *m*/*z* (ES^+^-MS): 319 ([*M*+H]^+^, 10 %), 341 ([*M*+Na]^+^, 100 %), 420 ([*M*+Et_3_N]^+^, 30 %), 659 ([2 *M*+Na]^+^, 24 %); elemental analysis calcd (%) for C_12_H_22_N_4_O_6_: C 45.28, H 6.97, N 17.60; found: C 45.17, H 6.98, N 17.17. Crystal data for **1** form A: C_12_H_22_N_4_O_6_, *M*_r_=318.34, colourless prism, 2.02×0.36×0.02 mm^3^, oversized uncut crystal due to fraying of layers on cutting, crystallised from methanol, triclinic space group *P*$\bar 1$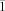, *a*=4.595(1), *b*=5.714(1), *c*=15.744(3) Å, *α*=96.49(2)°, *β*=91.59(2)°, *γ*=111.21(2)°, *V*=381.9(1) Å^3^, *Z=*1, *ρ*_calcd_=1.384 g cm^−3^, *F*_000_=170, Xcalibur, Sapphire3, Gemini ultra, Mo_Kα_ radiation, *λ*=0.71073 Å, *T*=100(2) K, 2*θ*_max_=58.2°, 5401 reflections collected, 1808 unique (*R*_int_=0.0390). Final GoF=1.055, *R*_1_=0.0421, *wR*_2_=0.102, *R* indices based on 1492 reflections with *I*≥2*σ*(*I*) (refinement on *F*^2^), 144 parameters, 0 restraints. Crystal data for **1** form B: C_12_H_22_N_4_O_6_, *M*_r_=318.34, colourless needle, 0.3×0.001×0.001 mm^3^, crystallised from methanol, monoclinic space group *P*2_2_/*n*, *a*=4.554(3), *b*=17.53(1), *c*=10.104(7) Å, *β*=101.686(8)°, *V*=790(1) Å^3^, *Z*=2, *ρ*_calcd_=1.339 g cm^−3^, *F*_000_=340, Kappa Rigaku Saturn724+, instrument I19, Diamond Light Source, synchrotron radiation, *λ*=0.6889 Å, *T*=120(2) K, 2*θ*_max_=48.5°, 5648 reflections collected, 1369 unique (*R*_int_=0.0869). Final GoF=0.936, *R*_1_=0.0665, *wR*_2_=0.166, *R* indices based on 844 reflections with *I*≥2*σ*(*I*) (refinement on *F*^2^), 101 parameters, 2 restraints; Lp correction applied, *μ*=0.108 mm^−1^.

**Methyl (2*S*)-2-[4-[[(1*S*)-2-methoxy-1-methyl-2-oxo-ethyl]carbamoylamino]butyl carbamoylamino]propanoate (2)**: L-Alanine methyl ester hydrochloride (0.94 g, 6.7 mmol) was dissolved in chloroform (50 mL) to give a clear solution. Triethylamine (0.7 g, 6.7 mmol) was added, resulting in a cloudy suspension that did not clear upon heating to 70 °C. 1,4-Diisocyanatobutane (0.47 g, 3.3 mmol) in chloroform (20 mL) was then added dropwise. After 1 h, the solution was cooled to room temperature and the white precipitate that had formed was filtered and washed with DCM and diethyl ether. The compound was dried in a drying pistol for 30 min to give a white powder (0.94 g, 2.7 mmol, 81 % yield): ^1^H NMR (700 MHz, [D_6_]DMSO): *δ*=6.16 (1 H, d, *J*=7.6 Hz, CHN*H*), 5.94 (1 H, t, *J*=5.7 Hz, N*H*CH_2_), 4.11 (1 H, p, *J*=7.3 Hz, C*H), 3.58 (3 H, s, OC*H*_3_), 3.00–2.87 (2 H, m, NHC*H*_2_), 1.35–1.26 (2 H, m, CH_2_CH_2_C*H*_2_), 1.19 ppm (3 H, d, *J*=7.3 Hz, C*C*H*_3_); ^13^C{^1^H} NMR (176 MHz, [D_6_]DMSO): *δ*=174.74 (*C*OO), 157.77 (N*C*O), 52.10 (OCH_3_), 48.55 (C*H*), 39.37 (N*C*H_2_CH_2_), 27.89 (CH_2_CH_2_C*H*_2_), 18.46 ppm (C*CH_3_); *m*/*z* (ES^+^-MS): 369 ([*M*+Na]^+^, 100 %), 715 ([2 *M*+Na]^+^, 30 %), 242 ([fragment−2COOMe], 30 %); elemental analysis calcd (%) for C_14_H_26_N_4_O_6_: C 48.55, H 7.57, N 16.17; found: C 48.20, H 7.52, N 15.95. Crystal data for **2** form A: C_14_H_26_N_4_O_6_, *M*_r_=346.39, colourless plate, 0.5×0.1×0.03 mm^3^, crystallised from methanol, triclinic, space group *P*1, *a*=4.645(2), *b*=6.071(2), *c*=16.165(3) Å, *α*=93.37(2)°, *β*=95.51(2)°, *γ*=109.60(3)°, *V*=425.4(2) Å^3^, *Z*=1, *ρ*_calcd_=1.352 g cm^−3^, *F*_000_=186, Bruker SMART CCD 6000 area detector, Mo_Kα_ radiation, *λ*=0.71073 Å, *T*=120(2) K, 2*θ*_max_=55.0°, 3525 reflections collected, 2807 unique (*R*_int_=0.0849). Final GoF=0.955, *R*_1_=0.0806, *wR*_2_=0.213, *R* indices based on 2901 reflections with *I*≥2*σ*(*I*) (refinement on *F*^2^), 231 parameters, 27 restraints; Lp and absorption corrections applied, *μ*=0.106 mm^−1^.

**Methyl 2-[6-[(2-methoxy-2-oxo-ethyl)carbamoylamino]hexylcarbamoylamino]acetate (3)**: Glycine methyl ester hydrochloride (1.00 g, 7.96 mmol) was suspended in chloroform (30 mL) and an excess of triethylamine was added, whereupon the suspension dissolved. 1,6-Diisocyanatohexane (0.67 g, 3.98 mmol) in chloroform (20 mL) was added dropwise and the solution was then heated under reflux for 18 h, after which period a white suspension formed. The suspension was filtered, washed with water and methanol and dried in a drying pistol at 110 °C for 1 h. Compound **3** was isolated as a white powder (1.22 g, 3.50 mmol, 88 %): ^1^H NMR (500 MHz, [D_6_]DMSO): *δ*=6.15–6.08 (4 H, m, C*N*H*), 3.75 (4 H, d, *J*=6.0 Hz, COC*H*_2_NH), 3.60 (6 H, s, OCH_3_), 2.96 (4 H, dd, *J*=6.7, 12.8 Hz, NC*H*_2_), 1.38–1.31 (4 H, m, NCH_2_C*H*_2_), 1.27–1.20 ppm (4 H, m, CH_2_CH_2_C*H*_2_). ^13^C{^1^H} NMR (126 MHz, [D_6_]DMSO): *δ*=172.5 (*C*OO), 158.6 (N*C*O), 52.2 (*C*H_3_), 42.1 (N*C*H_2_), 40.7–39.7 (N*C*H_2_CH_2_ under DMSO residual solvent peak), 30.6 (NCH_2_*C*H_2_), 26.8 ppm (CH_2_CH_2_*C*H_2_); *m*/*z* (ES^+^-MS): 347 ([*M*+H]^+^, 40 %), 369 ([*M*+Na]^+^, 58 %), 370 ([*M*+H+Na]^+^, 100 %), 715 ([2 *M*+Na]^+^, 26 %); elemental analysis calcd (%) for C_14_H_26_N_4_O_6_: C 48.55, H 7.57, N 16.17; found: C 48.22, H 7.47, N 16.00. Crystal data for **3** form A: C_14_H_26_N_4_O_6_, *M*_r_=346.39, colourless plate, 0.01×0.008×0.002 mm^3^ crystallised from water, triclinic space group, *P*$\bar 1$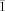, *a*=4.635(3), *b*=5.659(4), *c*=17.93(1) Å, *α*=83.189(7)°, *β*=83.317(7)°, *γ*=69.800(8)°, *V*=436.8(5) Å^3^, *Z*=1, *ρ*_calcd_=1.3167 g cm^−3^, *F*_000_=186, Kappa Rigaku Saturn724+, instrument I19, Diamond Light Source, synchrotron radiation, *λ*=0.6889 Å, *T*=150(2) K, 2*θ*_max_=51.0°, 3661 reflections collected, 1691 unique (*R*_int_=0.0419). Final GoF=1.073, *R*_1_=0.0522, *wR*_2_=0.135 (all data), *R* indices based on 1243 reflections with *I*≥2*σ*(*I*) (refinement on *F*^2^), 160 parameters, 0 restraints; Lp correction applied, *μ*=0.097 mm^−1^.

**Methyl (2*S*)-2-[6-[[(1*S*)-2-methoxy-1-methyl-2-oxo-ethyl]carbamoylamino]hexyl carbamoylamino]propanoate (4)**: L-Alanine methyl ester hydrochloride (3.96 g, 28.5 mmol) was dissolved in chloroform (100 mL) and an excess of triethylamine (3 g, 29.7 mmol) was added. 1,6-Diisocyanatohexane (2.39 g, 14.25 mmol) was added dropwise then the solution was heated under reflux. After 1 h, the solution was cooled to room temperature and the gelatinous precipitate was filtered and washed with DCM (2×25 mL) and then diethyl ether (25 mL). The resulting solid was sonicated in warm water (∼40 °C) for 30 min then filtered, recrystallised from methanol, filtered again and washed with diethyl ether. The compound was dried in a drying pistol for 30 min and the product was obtained as a white powder (4.12 g, 11.0 mmol, 77 % yield). ^1^H NMR (700 MHz, [D_6_]DMSO): *δ*=6.16 (2 H, d, *J*=7.7 Hz, C*N*H*), 5.93 (2 H, t, *J*=5.6 Hz, N*H*CH_2_), 4.11 (2 H, p, *J*=7.3 Hz, C*H), 3.58 (6 H, s, OMe), 2.99–2.86 (4 H, m, NHC*H*_2_), 1.30 (4 H, d, *J*=6.4 Hz, NHCH_2_C*H*_2_), 1.23–1.17 ppm (4 H, m, CH_2_CH_2_C*H*_2_, C*CH_3_); ^13^C{^1^H} NMR (176 MHz, [D_6_]DMSO): *δ*=174.75 (*C*OO), 157.79 (N*C*O), 52.09 (OCH_3_), 48.55 (C*), 40.7–39.7 (N*C*H_2_CH_2_ under DMSO residual solvent peak), 30.36 (NCH_2_*C*H_2_), 26.51 (CH_2_CH_2_C*H*_2_), 18.45 ppm (C*CH_3_). *m*/*z* (ES^+^-MS): 375 ([*M*+H]^+^, 20 %), 397 ([*M*+Na]^+^, 100 %), 771 ([2 *M*+Na]^+^, 18 %); elemental analysis calcd (%) for C_16_H_30_N_4_O_6_: C 51.32, H 8.08, N 14.96; found: C 51.25, H 8.26, N 14.59. Crystal data for **4** form A: C_16_H_30_N_4_O_6_, *M*_r_=374.44, clear colourless plate, 0.01×0.008×0.003 mm^3^ crystallised from methanol, monoclinic space group *P*2_2_, *a*=4.681(2), *b*=35.33(2), *c*=6.149(3) Å, *β*=108.562(4)°, *V*=964.1(7) Å^3^, *Z*=2, *ρ*_calcd_=1.2897 g cm^−3^, *F*_000_=404, Kappa Rigaku Saturn724+, instrument I19, Diamond Light Source, synchrotron radiation, *λ*=0.6889 Å, *T*=150 K, 2*θ*_max_=55.5°, 8115 reflections collected, 3855 unique (*R*_int_=0.0627). Final GoF=1.758, *R*_1_=0.145, *wR*_2_=0.399 (all data), *R* indices based on 3504 reflections with *I*≥2*σ*(*I*) (refinement on *F*^2^), 244 parameters, 0 restraints; Lp and absorption corrections applied, *μ*=0.093 mm^−1^. Absolute structure could not be determined.

**Methyl (2*S*)-2-[6-[[(1*S*)-1-benzyl-2-methoxy-2-oxo-ethyl]carbamoylamino]hexyl carbamoylamino]-3-phenyl-propanoate (5)**: The compound was prepared in accordance with the published procedure.[23] L-Phenylalanine methyl ester dihydrochloride (1.30 g, 6 mmol) was suspended in chloroform (150 mL) by the slow addition of a slight excess of triethylamine (0.62 g, 6.1 mmol). Following heating and sonication of the mixture, a hot filtration was carried out to remove un-dissolved amino acid. 1,6-Diisocyanatohexane (0.50 g, 3.0 mmol) was dissolved in chloroform (50 mL) and slowly added over a period of 1 h. The solution was heated under reflux at 70 °C for 24 h. The chloroform was removed under vacuum and the compound was washed with warm water for 1 h. The suspension was filtered, washed with ethyl acetate and dried in a drying pistol for 30 min. The product was obtained as a white powder (1.26 g, 2.4 mmol, 80 %): ^1^H NMR (700 MHz, [D_6_]DMSO): *δ*=7.28 (4 H, t, *J*=7.6 Hz, Ar*H*), 7.21 (2 H, t, *J*=7.6 Hz, Ar*H*), 7.15 (4 H, d, *J*=7.2 Hz, Ar*H*), 6.12 (2 H, d, *J*=8.3 Hz, C*N*H*_2_), 6.06 (2 H, t, *J*=5.8 Hz, CH_2_N*H*), 4.36 (2 H, td, *J*=5.5, 8.1, 13.6 Hz, C*H), 3.59 (6 H, s, OC*H*_3_), 2.98–2.90 (6 H, m, C*CH and NHC*H*_2_), 2.87 (2 H, dd, *J*=8.0, 13.8 Hz, C*C*H*′), 1.34–1.24 (4 H, m, NHCH_2_C*H*_2_), 1.24–1.14 ppm (4 H, m, NHCH_2_CH_2_C*H*_2_); ^13^C{^1^H} NMR (176 MHz, [D_6_]DMSO): *δ*=173.8 (s, NCO), 158.0 (COO), 137.8 (Ar*C*), 129.9 (Ar*C*), 129.0 (Ar*C*), 127.3 (Ar*C*), 54.7 (C*), 52.4 (O*C*H_3_), 41.0–39.5 (m, DMSO+NH*C*H_2_), 38.3 (C*CH_2_), 30.6 (NHCH_2_*C*H_2_), 26.8 ppm (NHCH_2_CH_2_*C*H_2_); *m*/*z* (ES^+^-MS): 527 ([*M*+H]^+^, 78 %), 549 ([*M*+Na]^+^, 88 %), 1052 ([2 *M*+H]^+^, 13 %), 1074 ([2 *M*+Na]^+^, 100 %); elemental analysis calcd (%) for C_28_H_38_N_4_O_6_: C 63.86, H 7.27, N 10.64; found: C 63.58, H 7.24, N 10.77.

**Methyl (2*S*)-2-[6-[[(1*S*)-2-methoxy-2-oxo-1-(pyren-1-ylmethyl)ethyl]carbamoylamino] hexylcarbamoylamino]-3-pyren-1-yl-propanoate (6)**: BOC-protected 1-pyrenylalanine methylester (see the Supporting Information; 0.07 g, 0.17 mmol) was stirred at room temperature in 1:1 dry TFA/DCM (4 mL). NMR spectroscopy indicated deprotection had occurred within 1 h. The solvent was removed under vacuum and the product was re-suspended in chloroform (10 mL). Triethylamine was added dropwise to produce a neutral solution, as monitored using indicator paper. 1,6-Diisocyanatohexane (0.013 g, 0.08 mmol) was added dropwise and the solution was heated under reflux for 18 h. The mixture was cooled and the fine precipitate that had formed was filtered and washed with cold chloroform and then diethyl ether. The precipitate was sonicated as a suspension in distilled water (5 mL), filtered and dried in a drying pistol for 30 min. The product was obtained as a cream-coloured solid (yield: 0.039 g, 0.05 mmol, 30 %). ^1^H NMR (700 MHz, [D_6_]DMSO): *δ*=8.39 (2 H, d, *J*=9.2 Hz, Ar-*H*), 8.32 (4 H, t, *J*=8.6 Hz, Ar-*H*), 8.26 (4 H, dd, *J*=17.1, 8.5 Hz, Ar-*H*), 8.18 (4 H, s, Ar-*H*), 8.11 (2 H, t, *J*=7.5 Hz, Ar-*H*), 7.92 (2 H, d, *J*=7.8 Hz, Ar-*H*), 6.38 (2 H, d, *J*=8.2 Hz, C*N*H*), 6.07 (2 H, t, *J*=5.3 Hz, N*H*CH_2_), 4.70 (4 H, dd, *J*=15.0, 7.6 Hz, C*H), 3.79 (2 H, dd, *J*=14.1, 6.2 Hz, C*C*H*), 3.67 (2 H, dd, *J*=13.8, 7.9 Hz, C*C*H*′), 3.67 (6 H, s, OC*H*_3_), 2.94 (4 H, dd, *J*=13.1, 6.5 Hz, NHC*H*_2_), 1.32–1.24 (4 H, m, NHCH_2_C*H*_2_), 1.20–1.11 ppm (4 H, m, CH_2_CH_2_C*H*_2_); ^13^C{^1^H} NMR (176 MHz, [D_6_]DMSO): *δ*=173.8 (*C*OO), 158.1 (S, N*C*O), 138.4 (Ar*C*), 131.6 (Ar*C*), 131.1 (s, Ar*C*), 130.6 (Ar*C*), 129.5 (Ar*C*), 129.1(Ar*C*), 128.2 (Ar*C*), 128.1 (Ar*C*), 127.6 (Ar*C*), 126.9 (Ar*C*), 125.9 (Ar*C*), 125.7 (Ar*C*), 125.4 (Ar*C*), 124.9 (Ar*C*), 124.8 (Ar*C*), (Ar*C*), 123.9 (Ar*C*), 55.1 (C*), 52.5 (O*C*H_3_), 40.3 (N*C*H_2_ under DMSO peak), 36.1 (C*CH_2_), 30.6 (NCH_2_*C*H_2_), 26.8 ppm (NCH_2_CH_2_*C*H_2_); *m*/*z* (ASAP^+^, 650 °C): 215 ([PyCH_2_]^+^, 100 %), 710 ([*M*−2 MeOH]^+^, 55 %); elemental analysis calcd (%) for C_48_H_46_N_4_O_6_**⋅**0.15 CHCl_3_: C 73.01, H 5.87, N 7.07; found: C 72.98, H 5.94, N 7.11.

**Methyl (2*S*)-2-[6-[[(1*S*)-2-methoxy-2-oxo-1-(pyren-2-ylmethyl)ethyl]carbamoylamino] hexylcarbamoylamino]-3-pyren-2-yl-propanoate (7)**: BOC-protected 2-pyrenylalanine methylester (see the Supporting Information; 0.12 g, 0.296 mmol) was stirred at room temperature in dry 1:1 TFA/CDCl_3_ (4 mL). NMR spectroscopy indicated deprotection had occurred within 1 h. The solvent was removed under vacuum and the product was re-suspended in chloroform (10 mL). Triethylamine was added dropwise to produce a neutral solution, monitored using indicator paper. 1,6-Diisocyanatohexane (0.025 g, 0.148 mmol) was then added dropwise and the solution was heated under reflux at 70 °C for 18 h. The mixture was cooled and the precipitate that had formed was filtered and washed with cold chloroform and then diethyl ether. The product was obtained as an off-white solid (yield: 0.072 g, 0.093 mmol, 31 %). ^1^H NMR (700 MHz, [D_6_]DMSO): *δ*=8.28 (4 H, d, *J*=7.5 Hz, Ar-*H*), 8.17 (4 H, d, *J*=7.5 Hz, Ar-*H*), 8.12 (4 H, d, *J*=7.5 Hz, Ar-*H*), 8.10 (4 H, s, Ar-*H*), 8.05 (2 H, t, *J*=7.5 Hz, Ar-H), 6.26 (2 H, d, *J*=8.2 Hz, C*N*H*), 6.03 (1 H, t, *J*=5.4 Hz, N*H*CH_2_), 4.67 (2 H, dd, *J*=13.8, 8.1 Hz, C*H), 3.64 (6 H, s, OC*H*_3_), 3.44 (2 H, dd, *J*=13.8, 5.5 Hz, C*C*H*), 3.34 (2 H under H_2_O solvent peak, C*C*H*′) 2.87 (4 H, dd, *J* 12.9, 6.5, NHC*H*_2_), 1.23–1.19 (4 H, m, NHCH_2_C*H*_2_), 1.10–1.08 ppm (3 H, m, CH_2_CH_2_C*H*_2_); ^13^C{^1^H} NMR (176 MHz, [D_6_]DMSO): *δ*=173.8 (s, *C*OO), 158.0 (N*C*O), 136.0 (Ar*C*), 131.2 (Ar*C*), 131.1 (Ar*C*), 128.1 (Ar*C*), 127.8 (Ar*C*), 126.6 (Ar*C*), 126.5 (Ar*C*), 125.7 (Ar*C*), 124.3 (Ar*C*), 123.3 (Ar*C*), 55.1 (C*), 52.4 (O*C*H_3_), 46.4 (N*C*H_2_), 38.9 (C*CH_2_), 30.5 (NCH_2_*C*H_2_), 26.6 ppm (NCH_2_CH_2_*C*H_2_); *m*/*z* (ASAP^+^, 550 °C): 215 ([PyCH_2_]^+^, 100 %), 439 ([*M*−C_20_H_17_NO_2_−MeOH]^+^, 35 %), 710 ([*M*−2 MeOH]^+^, 5 %); elemental analysis calcd (%) for C_48_H_46_N_4_O_6_**⋅**0.5 CHCl_3_: C 69.80, H 5.62, N 6.71; found: C 69.88, H 5.79, N 6.68.
