# Supplementary material for: Blending Gelators to Tune Gel Structure and Probe Anion-Induced Disassembly
Source: Chemistry. 2013 Dec 2;20(1):279–91. doi: 10.1002/chem.201303153 (PMC3994942; doi:10.1002/chem.201303153)
Supplement: Supplementary file 1 [file chem0020-0279-sd1.pdf]

CHEMISTRY
A EUROPEAN JOURNAL

Supporting Information

© Copyright Wiley-VCH Verlag GmbH & Co. KGaA, 69451 Weinheim, 2013

Blending Gelators to Tune Gel Structure and Probe Anion-Induced Disassembly

Jonathan A. Foster,[a] Robert M. Edkins,[a] Gary J. Cameron,[a] Neil Colgin,[a] Katharina Fucke,[a] Sam Ridgeway,[a] Andrew G. Crawford,[a] Todd B. Marder,[b] Andrew Beeby,[a] Steven L. Cobb,[a] and Jonathan W. Steed*[a]

chem_201303153_sm_miscellaneous_information.pdf

S1 Synthesis

All solvents and reagents were obtained from standard commercial sources. The drying pistol was evacuated using an aspirator and heated by toluene under reflux. All NMR spectra were performed on a Varian Mercury-400 (400 MHz for 1H), Varian Inova-500 machine (500 MHz for ^{1}H, 126 Hz for ^{13}C) or a Varian DD-700 (700 MHz for ^{1}H, 176 MHz for ^{13}C) and were referenced to residual solvent. Mass spectrometry for compounds which dissolve in methanol was undertaken using a Thermo-Finnigan LTQ FT machine running in positive electron spray (ES) mode. Insoluble compounds were measured using a Waters Xevo QTOF spectrometer equipped with an Atmospheric Solids Analysis Probe (ASAP). Elemental analysis was performed using an Exeter Analytical inc. CE-400 Elemental Analyser.

(*S*)-Methyl-2-(*tert*-Butoxycarbonylamino)-3-(pyren-1-yl)propanoate

Acid washed zinc (0.400 g, 6.12 mmol) was agitated under vacuum at 100°C for 15 min. The temperature was reduced to 70°C and the reaction put under an atmosphere of argon. Iodine (0.010 g, 0.039 mmol) dissolved in DMF (1 mL) was added. After stirring for 15 min, *N*-(*tert*-butoxycarbonyl)-3-iodo-L-alanine methylester[1] (0.486 g, 1.52 mmol) dissolved in DMF (1 mL) was added and the mixture stirred for a further 15 min. At this point the temperature was reduced to 50°C and $Pd(dba)_2$ (0.030 g, 0.052 mmol), $P(O\text{-}Tol)_3$ (0.040 g, 0.13 mmol) and 1-bromopyrene (0.430 g, 1.53 mmol) were added. The reaction was left to stir for 5hours after which time the temperature was reduced to rt and the reaction left to stir overnight. The crude product was separated from the reaction mixture by silica gel column chromatography, initially using Hex-EtOAc (95:5) as the elutant. A second silica gel column using Hex-EtOAc (99:1) as the elutant, afforded (*S*)-Methyl-2-(*tert*-butoxycarbonylamino)-3-(pyren-1-yl)propanoate (0.243 g, 40%) as a white solid. m.p. 138.6-139.5 ^{o}C; IR (neat) 3354, 2962, 1755, 1686, 1520, 1244 cm^{-1}; ^{1}H NMR (700 MHz,$CDCl_3$): 8.31 (1 H, d, *J* 9.1, Ar-<u>H</u>), 8.18 (2H, m, Ar-<u>H</u>), 8.13 (1 H, d, *J* 9.8, Ar-<u>H</u>), 8.11 (1 H, d, *J* 7.7, Ar-<u>H</u>), 8.06-8.00 (3 H, m, Ar-<u>H</u>), 7.80 (1 H, d, *J* 7.7, Ar-<u>H</u>), 5.11 (1 H, d, *J* 7.0, N<u>H</u>), 4.85 (1 H, m, H-α), 3.87 (d1 H, d, *J* 14.0, 7.0, 1H, H-β), 3.79 (1 H, dd, *J* 14.0, 7.0, H-β), 3.61 (3 H, s, OC<u>H$_3$</u>), 1.40 (9 H, s, *t*Bu); ^{13}C NMR (176 MHz, $CDCl_3$) 172.6, 155.2, 131.5, 131.0, 130.7, 130.4, 129.8, 128.1, 128.0, 127.6, 127.3, 126.1, 125.3, 125.2, 125.1, 125.0, 124.9, 123.2, 80.1, 55.1, 52.4, 36.1, 28.4; HRMS (ESI +ve) *m/z* 404.1857 (M + H $C_{25}H_{26}NO_4$ requires 404.1862).

(*S*)-Methyl-2-(*tert*-Butoxycarbonylamino)-3-(pyren-2-yl)propanoate

Acid washed zinc (0.547 g, 8.37 mmol) was agitated under vacuum at 100 ^{o}C for 15 min. The temperature was reduced to 70°C and the reaction put under an argon atmosphere. Iodine

(0.010 g, 0.039 mmol) dissolved in DMF (1 mL) was added. After 15 min, *N*-(*tert*-Butoxycarbonyl)-3-iodo-L-alanine methylester[1] (0.640 g, 1.94 mmol) dissolved in DMF (1 mL) was added and the mixture stirred for a further 15 min. At this point the temperature was reduced to 50°C and $Pd(dba)_2$ (0.030 g, 0.052 mmol), $P(O\text{-}Tol)_3$ (0.040 g, 0.13 mmol) and 2-bromopyrene (0.547 g, 1.94 mmol) were added. After stirring for 5hours the temperature was reduced to rt and the reaction left to stir overnight. Silica gel column chromatography, using Hex-EtOAc (90:10) as the elutant, yielded an impure product. A second silica gel column, using Hex-EtOAc (95 : 5), afforded the pure product (*S*)-Methyl-2-(*tert*-butoxycarbonylamino)-3-(pyren-2-yl)propanoate (0.352 g, 45%) as a beige solid. m.p. 126.5-127.6°C; IR (neat) 3370, 2973, 2358, 1754, 1686, 1519, 1156 cm^{-1}; ^1H NMR (700MHz, $CDCl_3$) 8.18 (2 H, d, *J* 7.7, Ar-H), 8.07 (2 H, d, *J* 9.1, Ar-H), 8.02-7.99 (3 H, m, Ar-H), 7.95 (2 H, brs, Ar-H), 5.07 (1 H, brd, *J* 7.7, NH), 4.80 (1 H, m, H-α), 3.72 (3 H, s, OCH_3), 3.57 (1 H, dd, *J* 14.0, 6.3 1H, H-β), 3.50 (1 H, dd, *J* 14.0, 6.3 H-β), 1.41 (9 H, s, *t*Bu); ^{13}C NMR (176 MHz, $CDCl_3$) 172.5, 155.3, 134.0, 131.5, 131.1, 127.9, 127.2, 126.0, 125.8, 125.2, 124.6, 123.9, 80.16, 55.1, 52.4, 39.0, 28.5; HRMS (ESI +ve) *m/z* 404.1870 (M + H $C_{25}H_{26}NO_4$ requires 404.1862).

S2 Single Crystal X-ray diffraction measurements

Suitable single crystals were mounted using perflouropolyether on a thin glass fibre or preformed tip. Crystallographic measurements were carried out using a Bruker SMART 6000, Oxford Diffraction Gemini or Kappa Rigaku Saturn 724+. The instruments are equipped with a graphite monochromatic Mo-Kα radiation (λ = 0.71073) or synchrotron radiation (undulator, λ = 0.68890). The data collection temperature was maintained using by an open flow N_2 Oxford Cryostream device. Integration was carried out using SAINT, CrysAlisPro or Crystal Clear software. Data sets were corrected for Lorentz and polarization effects and for the effects of absorption. Structures were solved using direct methods and refined by full-matrix least squares on F^2 for all data using SHELXTL[2] and OLEX2.[3] All non-hydrogen atoms were treated as anisotropic. Hydrogen atoms were fixed in idealised positions and allowed to ride on the parent atom to which they are attached. Hydrogen atom thermal parameters were tied to those of the parent atom. Where possible N-H and O-H hydrogen atoms were located experimentally and their position and displacement parameters refined or their position parameters constrained to ideal distances from the parent atoms. Molecular graphics were produced using the program X-Seed.[4]

S3 Gel Formation

Gelators were screened for gelation behaviour against a range of solvents across the polarity spectrum. An amount corresponding to 1 % weight to volume (% w/v) of the compound relative to the solvent was weighed into a small vial, sealed and heated until fully dissolved using a heatgun. The samples were cooled rapidly by placing the vials in a water bath at room temperature and sonicating them briefly when the first signs of precipitation were observed.[5] The results upon cooling to room temperature are recorded in Table S3.1. Gel formation was characterised by a simple vial inversion test; if the solvent was fully immobilised it was considered to have gelled. The term partial gel was ascribed to samples where only partial trapping of the solvent occurred. Heating took place in sealed vials using a heat gun and in some cases the dissolution temperature of the compound was above the boiling point of the solvent at atmospheric pressure.

Table S3.1 Compounds **1-7** screened against a range of solvents for gelation behaviour by heating 1 % w/v of the compound in the corresponding solvent until fully dissolved and recording the results upon cooling.

Solvent	Compound						
	**1**	**2**	**3**	**4**	**5**	**6**	**7**
H_2O	P	P	P	G[[a]	I	I	I
DMSO	S	S	S	S	S	S	S
CH_3CN	P	P	P	G	G	G	G
MeOH	P	P	P	S	S	PG	G
EtOH	P	P	P	G[a]	S	G	G
Acetone	P	G	P	G	G	G	I
THF	P	G	P	G	S	G	G
DCM	P	G[a]	P	PG	S	G	I
EtOAc	P	G	P	G	G	PG	I
$CHCl_3$	P	G[a]	P	G	S	G	G
Et_2O	I	I	I	I	I	I	I
Toluene	G	G[a]	G	G	G	G	P
Hexane	I	I	I	I	I	I	I

P = precipitate, S = solution, G = Gel, PG = partial gel, O = oil, I = insoluble with heating, [a] = gel unstable, breaks down over time.

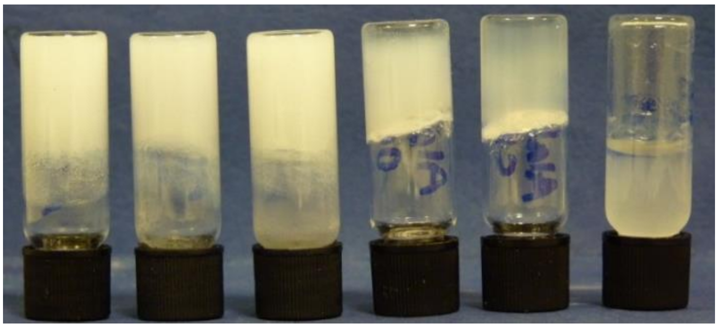

Figure S3.1 Photograph showing 1 % w/v gels of phenylalanine derived gelator **2** in (left to right) 3:2 $MeOH:H_2O$, 1:4 $THF:H_2O$, 1:1 $DMSO:H_2O$, CH_3CN, Ethyl acetate, Acetone, Benzene, Toluene

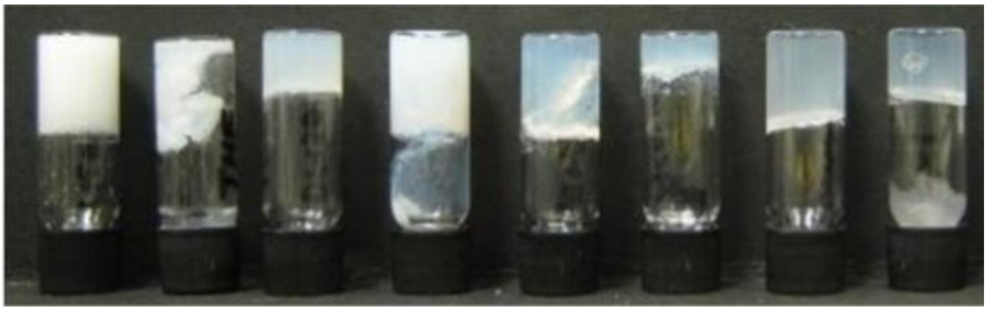

Figure S3.2 Photograph showing 1 % w/v gels of phenylalanine derived gelator **5** in (left to right) 3:2 $MeOH:H_2O$, 1:4 $THF:H_2O$, 1:1 $DMSO:H_2O$, CH_3CN, Ethyl acetate, Acetone, Benzene, Toluene

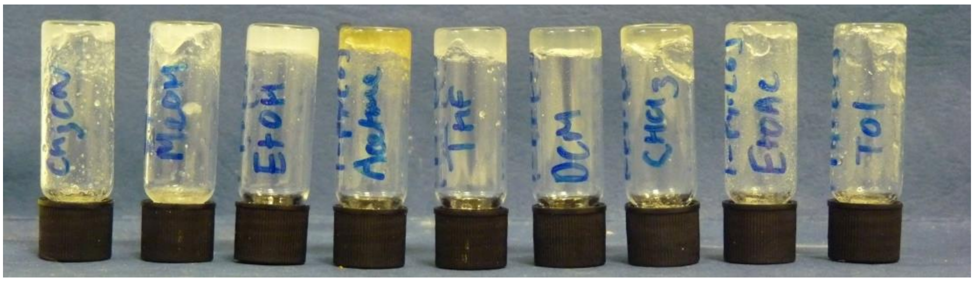

Figure S3.3 Gels containing 1 % w/v **6** in (left to right): acetonitrile, methanol, ethanol, acetone, tetrahydrofuran, dichloromethane, chloroform, ethyl acetate and toluene.

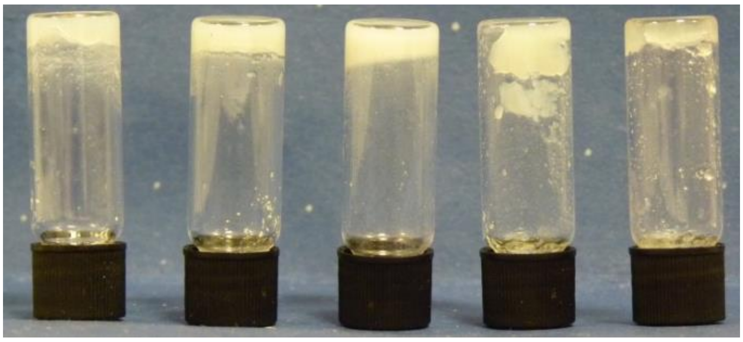

Figure S3.4 Gels of **7** at 1 % w/v in (left to right): acetonitrile, methanol, ethanol, tetrahydrofuran, chloroform

S4 Solid state analysis

Samples were prepared for solid state analysis by rapid removal of the solvent from freshly prepared gels by absorption of the solvent into filter paper followed by vacuum desication. Powder diffraction was performed on glass slides using a Brucker D8 or D5000 X-Ray Diffractometers using CuKα radiation at a wavelength of 1.5406 Å. Differential scanning calorimetry was undertaken on a Perkin Elmer Pyris 1 DSC or TA instruments Q1000 DSC using 1 mg of sample weighed into the centre of an aluminium pan and heated from 0-220°C at 10°C per minute. Fourier transform infrared spectra were recorded with a Perkin Elmer Spectrum 100 ATR instrument (Perkin-Elmer, Norwalk, Ct., USA). For each spectrum, 16 scans were conducted over a spectral range of 4000 to 600 cm^{-1} with a resolution of 4 cm^{-1}.

XRPD analysis of samples of **1** (Figure S4.1) isolated as toluene xerogels or dried precipitates from the other solvents indicated the presence of solely form A (by comparison with the XRPD pattern calculated from the single crystal data). A second form is obtained by heating solid compound **1** in toluene which results in the formation of gelatinous aggregates which partially gel the solvent before the compound has fully dissolved. Comparison of the XRPD pattern of this gelatinous material once dried with calculated patterns for the single crystals of **1** indicates that it consists predominantly of form B, with small amounts of form A also present. Upon further heating of the sample, the gelator fully dissolves and gels can be formed upon cooling using the regular procedure. DSC analysis of the solid form A xerogels of **1** from cooled toluene solutions gives a melting onset of 178 °C followed by recrystallization and a second melting endotherm with onset 194 °C (Figure S4.12). In contrast, the form B xerogels isolated from hot toluene show only one melting endotherm at 196 °C (Figure S4.13).

Two different polymorphs are also observed for butylene-spaced alanine derivative **2** and both may be obtained from gels. The XRPD pattern of xerogels (Figure S4.2) obtained from acetone matches the single crystal data and hence this form is designated form A, whilst xerogels obtained from toluene and ethyl acetate show a different XRPD pattern assigned as a form B. The precipitate obtained from acetonitrile is found to be an approximately 1:1 mixture of forms A and B. Toluene gels allowed to evaporate slowly over several days produce powder patterns matching those of Form A, in contrast to rapidly dried fresh samples which show patterns assigned as Form B. This time dependence suggests conversion of form B to form A over time, or upon drying, suggesting form B is metastable and that form A is the thermodynamic form under ambient conditions.

The non-gelling precipitates of **3** obtained from acetone and acetonitrile gave XRPD patterns (Figure S4.3) which match those generated for the crystal structure of **3**. However, XRPD patterns for the xerogels of **3** obtained from toluene do not match those of form A and are assigned as a new form, form B, of unknown structure. The XRPD pattern for the precipitate of **3** obtained from ethyl acetate showed a mixture of predominantly form A with form B as a minor component.

The XRPD patterns for xerogels from of the hexamethylene spacer alanine derived **4** (Figure S4.4) obtained from all four solvents (acetonitrile, acetone, ethyl acetate and toluene) show a pattern which is different to that of the known crystal structure (form A), so are assigned form B. Both ethanol and water gels of **4** rapidly break down to give a precipitates with an XRPD pattern that indicates the presence of a mixture of the gelling form B and another form with an XRPD pattern resembling that of form A, although the presence of a third form cannot be ruled out.

XRPD patterns for xerogels of **5** formed from acetonitrile and acetone closely match one another (Figure S4.5). However, the XRPD patterns for the toluene and ethyl acetate samples are weakly diffracting and an assignment could not be made on the basis of the broad, poorly defined XRPD patterns. IR spectra for all of the samples (Figure S4.6) show a strong match and on this basis the xerogels are all assigned as formB. Xerogels produced from gels of **5** in a number of binary solvent mixtures were also investigated. DMSO:water (1:1) and THF:water (1:4) samples gave XRPD patterns which match those obtained from acetonitrile and acetone (form B), whilst xerogels formed from gels of methanol:water (3:2) gave different XRPD patterns, assigned as form A.

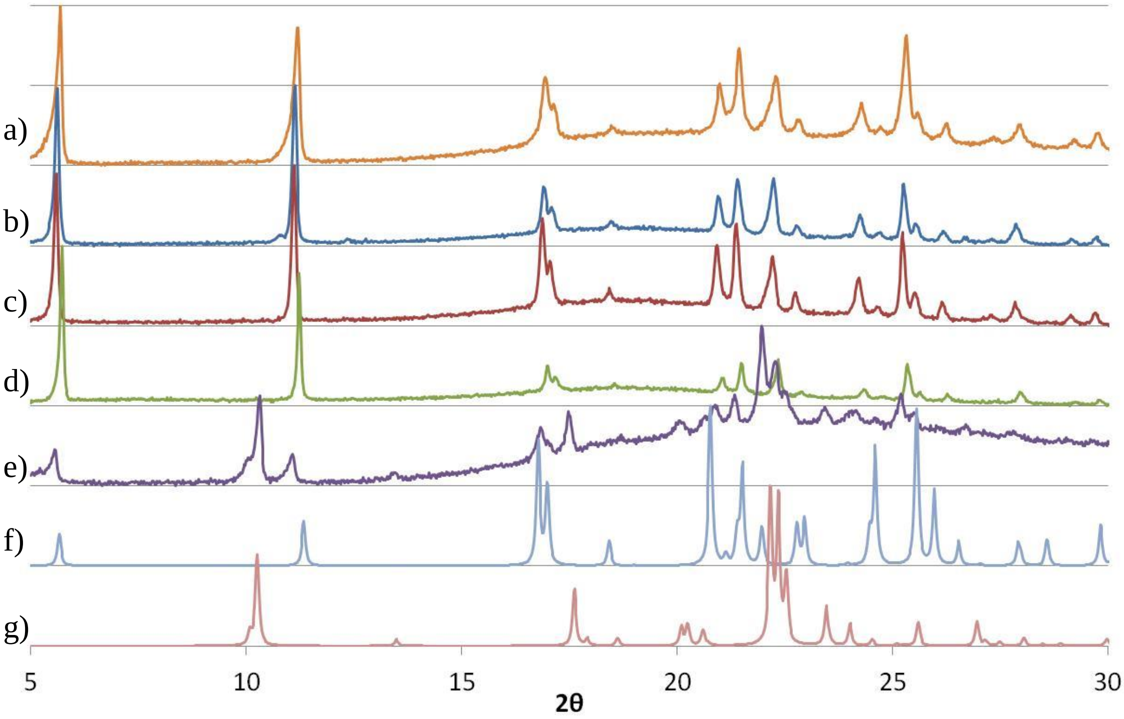

Figure S4.1 XRPD patterns showing recrystallisation of 1 % w/v **1** from: a) toluene- form A (orange), b) ethyl acetate- form A (blue), c) acetone- form A (red), d) acetonitrile- form A (green), e) in toluene at high temperatures- form B(+A) (purple). Calculated crystal structures of f) compound **1** form A (mid blue) and g) form B (pink).

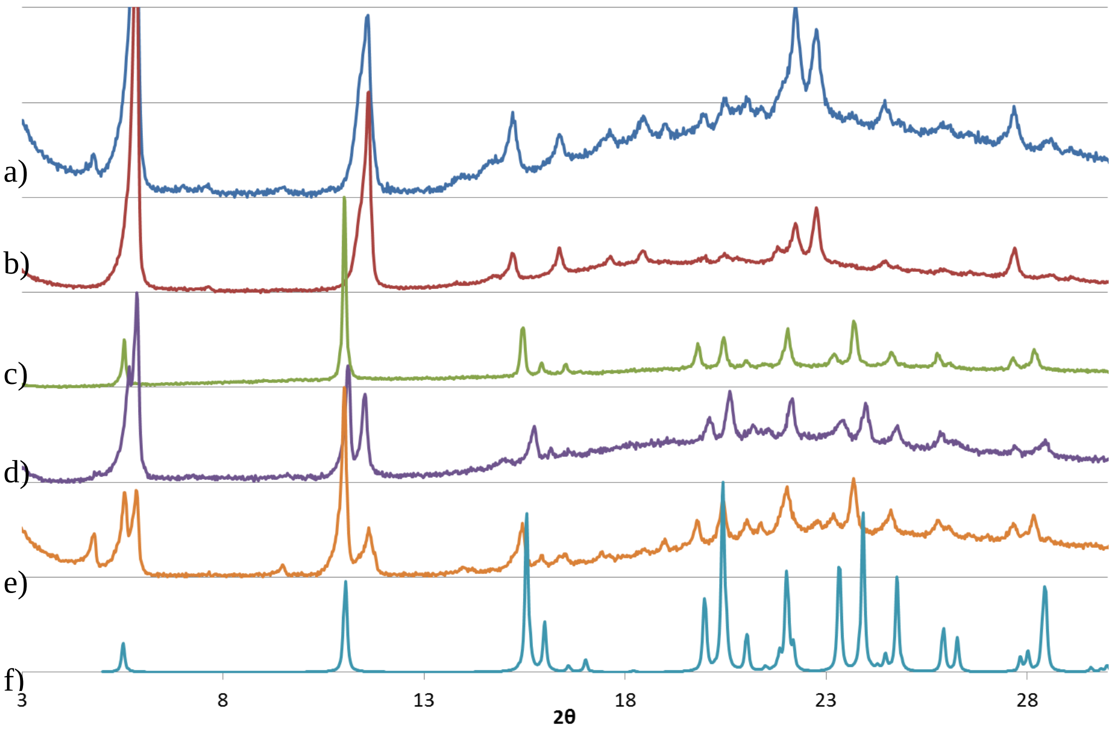

Figure S4.2 XRPD patterns showing recrystallisation of 1 % w/v **2** from: a) toluene- form B (blue), b) ethyl acetate- form B (red), c) acetone- form A (green), d) acetonitrile- form A and B (purple), e) by slow evaporation from toluene- form A(+B) (orange). Calculated crystal structures of f) compound **2** form A (mid blue)

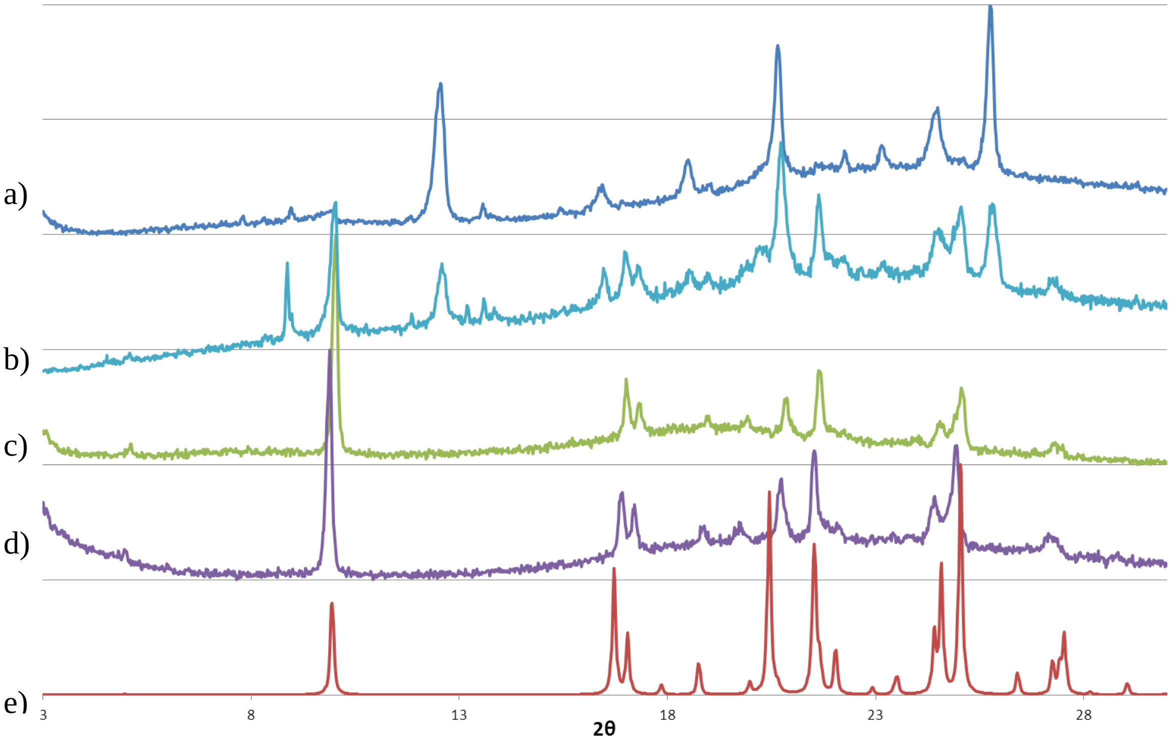

Figure S4.3 XRPD patterns showing recrystallisation of 1 wt% **3** (top to bottom) from toluene- form B (blue), ethylacetate- Form A (+B) (light blue), acetone- form A (green), acetonitrile- form A (purple). Calculated crystal structures of **3** form A (red)

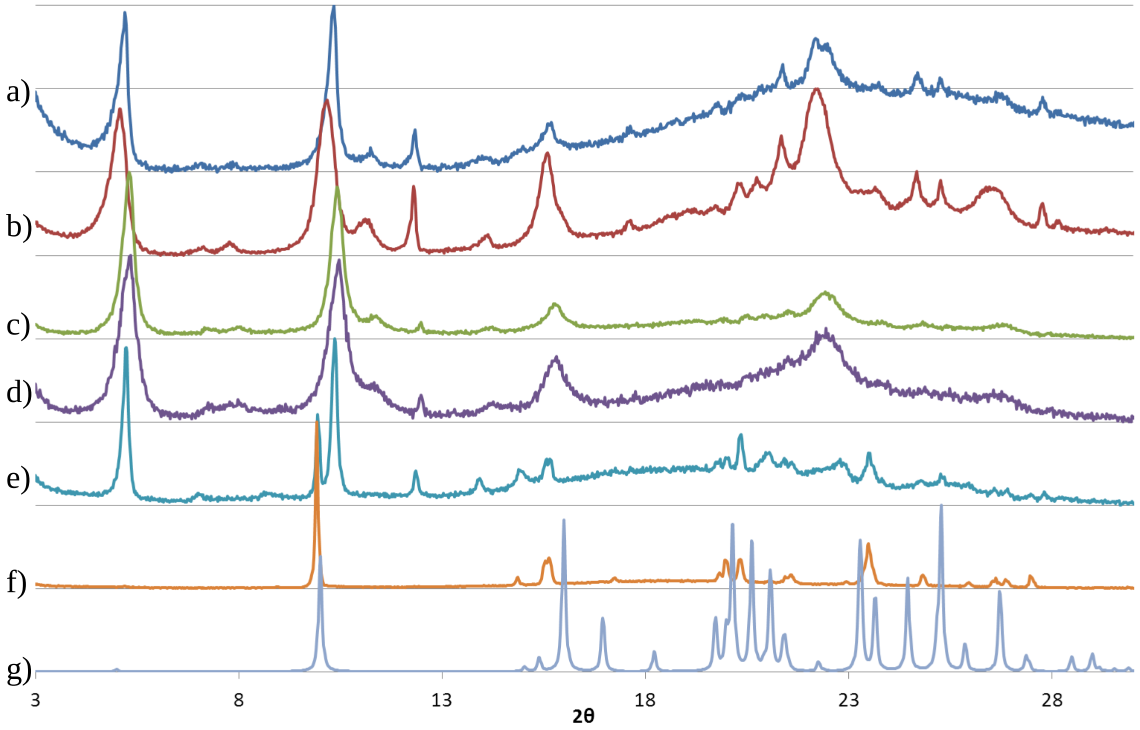

Figure S4.4 XRPD patterns showing recrystallisation of 1 % w/v **4** from: a) toluene- form B (blue), b) ethyl acetate- form B (red), c) acetone- form B (green), d) acetonitrile- form B (purple), e) water– form B and another form resembling form A (light blue), f) broken down gel from water- Form A or a new form (orange). g) Calculated XRPD pattern from the single crystal structure of **4** form A (grey)

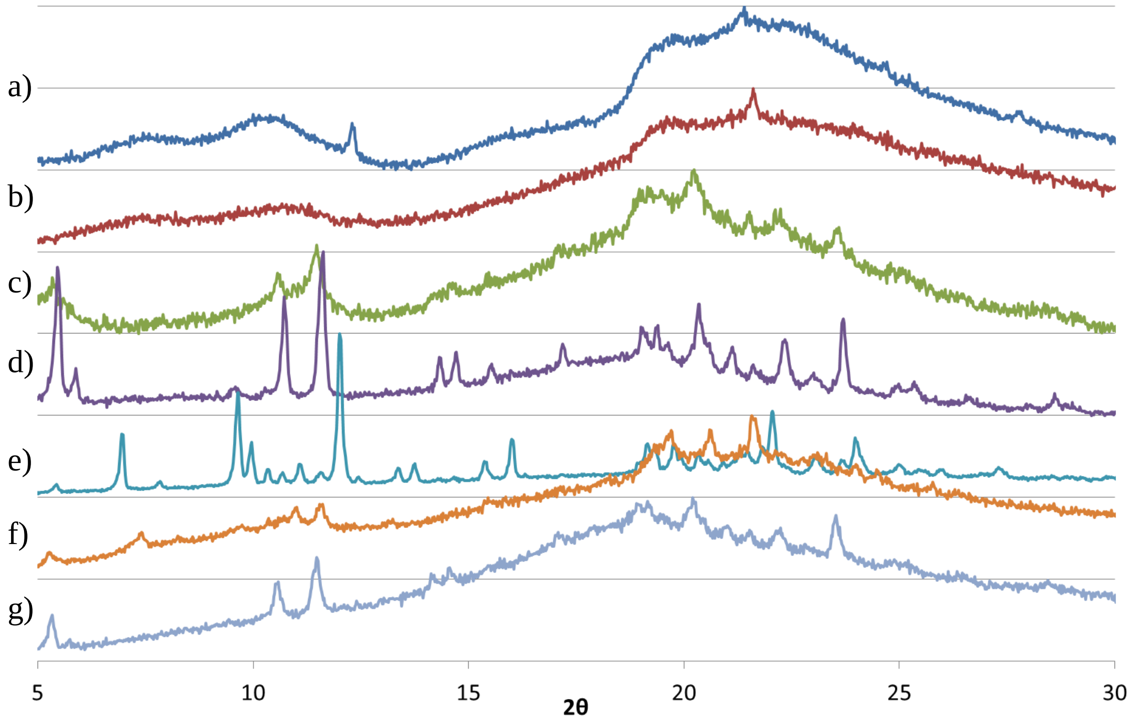

Figure S4.5 XRPD patterns showing recrystallisation of 1 % w/v **5** from: a) toluene- form B (blue), b) ethyl acetate- form B (red), c) acetone- form B (green), d) acetonitrile- form B (purple), e) 3:2 methanol:water- form A, f) 1:4 THF:water- form B g) 1:1 DMSO:water- form B.

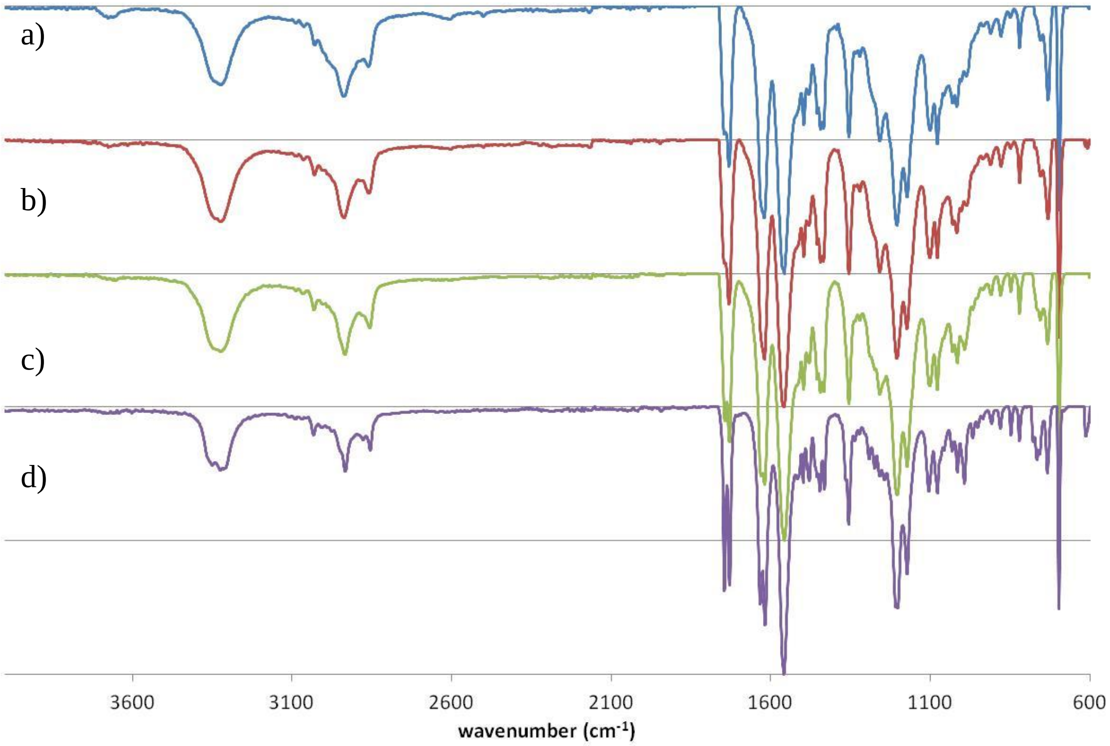

Figure S4.6 IR spectra showing recrystallisation of 1 wt% **5** a) from toluene (blue), b) ethylacetate (red), c) acetone (green), d) acetonitrile (purple). All compounds are assigned as form B.

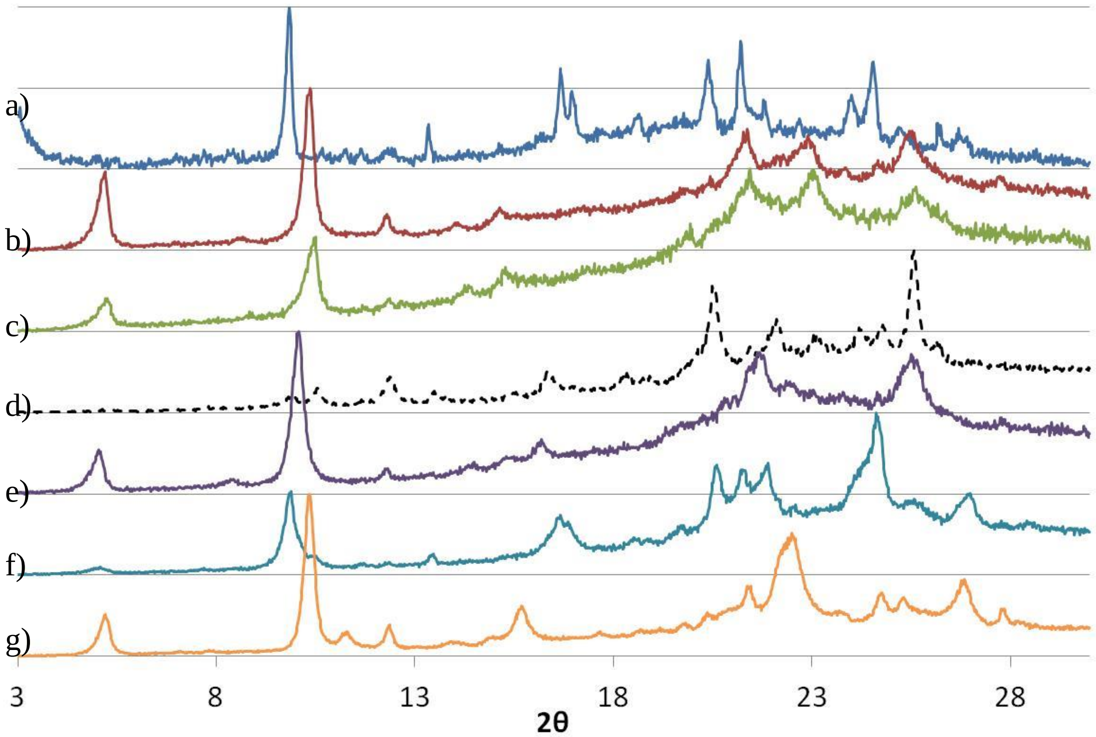

Figure S4.7 XRPD patterns corresponding to xerogels formed from toluene of **3**:**4** in a ratio of a) 1:0 (blue), b) 4:1 (red), c) 3:2 (green), d) 1:1 (dotted black), e) 2:3 (purple), f) 1:4 (light blue), g) 0:1 (orange).

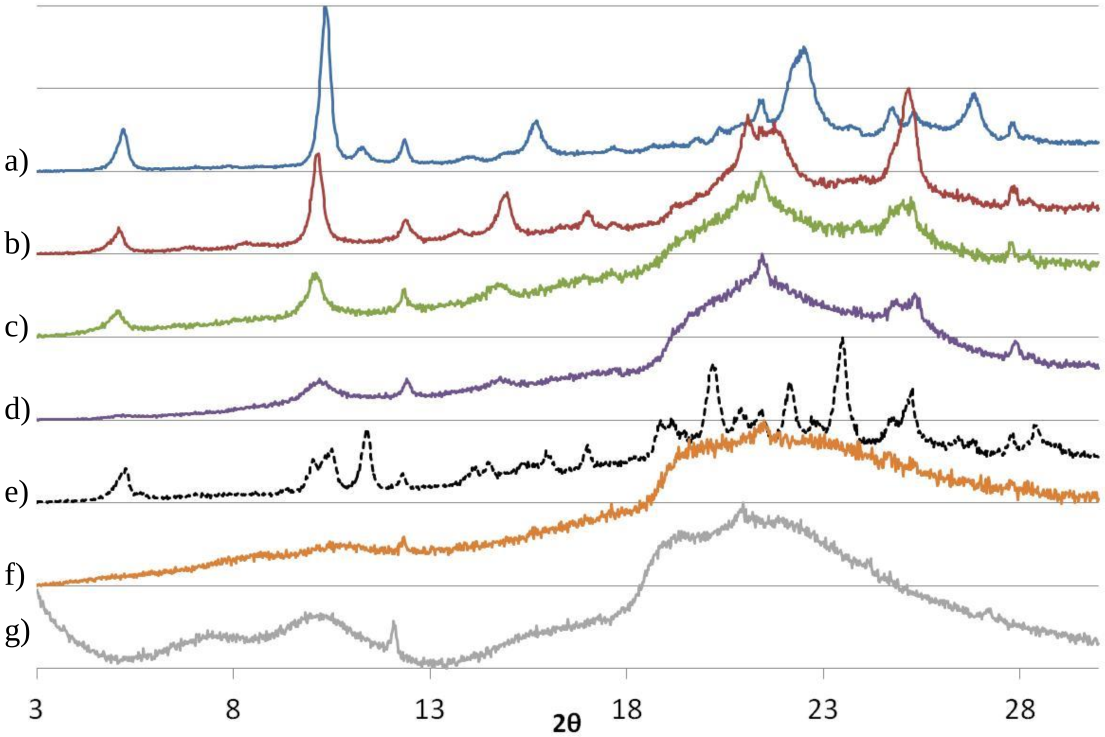

Figure S4.8 XRPD patterns corresponding to xerogels formed from toluene of **4**:**5** in a ratio of a) 1:0 (blue), b) 4:1 (red), c) 3:2 (green), d) 1:1 (purple), e) 2:3 (dotted black), f) 1:4 (orange) and g) 0:1 (grey).

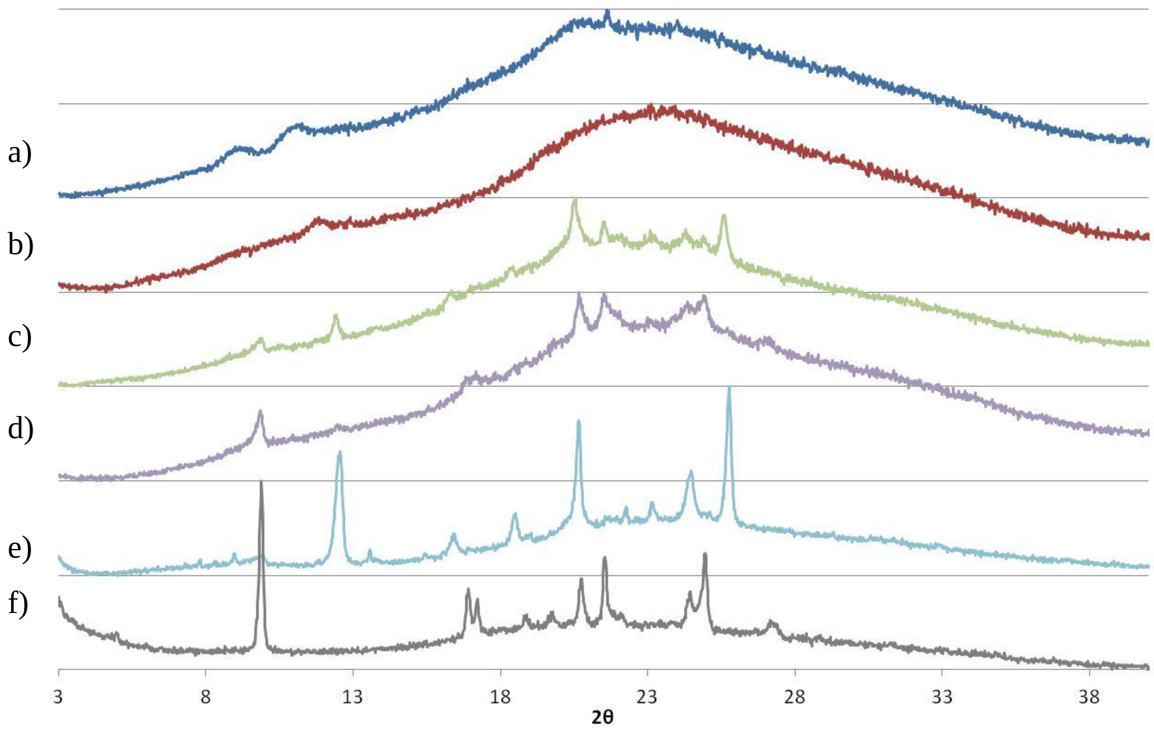

Figure S4.9 XRPD patterns for xerogels prepared from fast cooled toluene gels with 1 % w/v: a) **6** (dark blue), b) **7** (red), c) 1:9 **6**:**3** (green), d) 1:9 **7**:**3** (purple), e) **3** form B (light blue). f) Pattern for non-gel forming precipitate from acetonitrile of **3**, form A (grey).

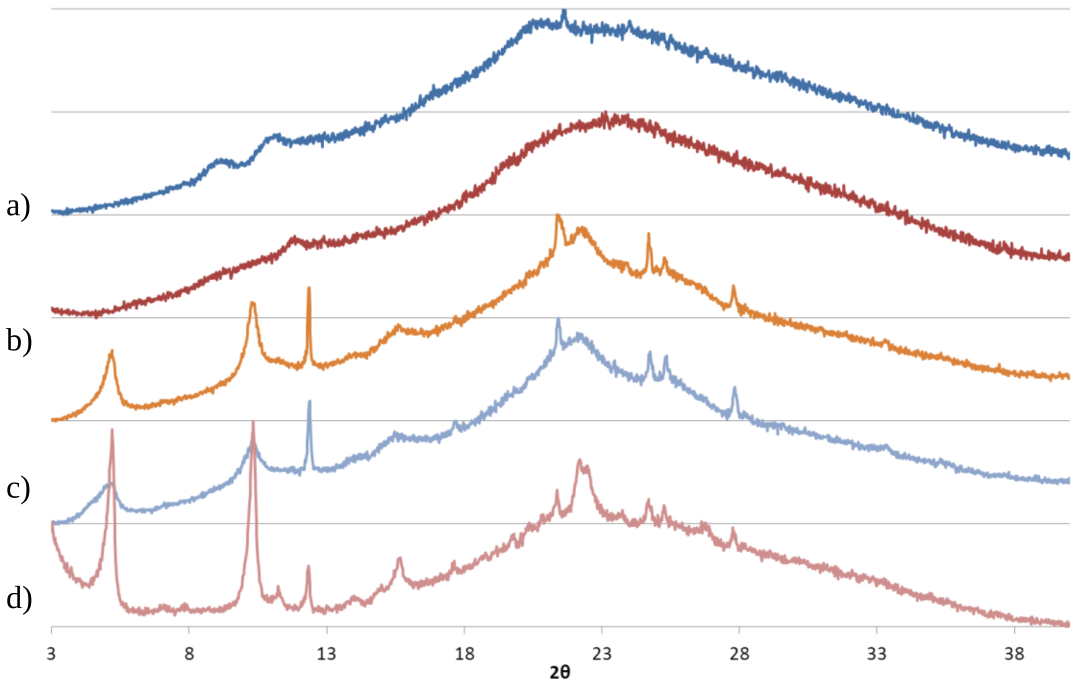

Figure S4.10 XRPD patterns for xerogels prepared from fast cooled toluene gels with 1 % w/v: a) **6** (dark blue), b) **7** (red), c) 1:9 **6**:**4** (orange), d) 1:9 **7:4** (light blue), e) **4** (pink).

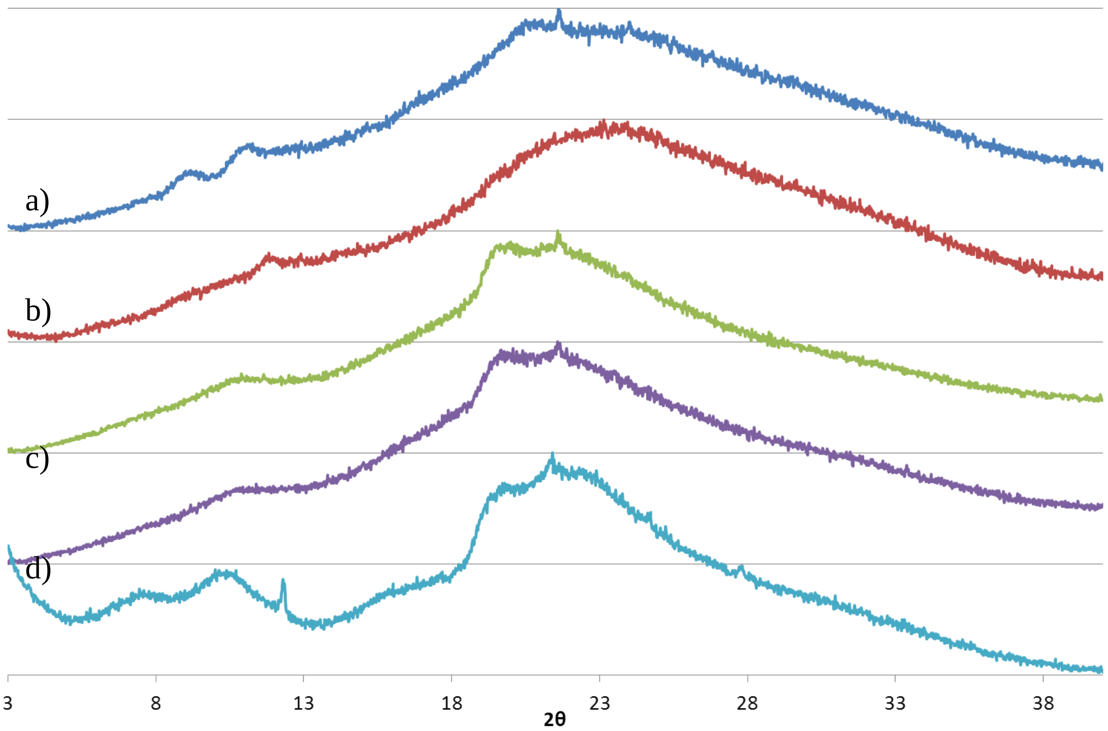

Figure S4.11 XRPD patterns for xerogels prepared from fast cooled toluene gels with 1 % w/v: a) **6** (dark blue), b) **7** (red)**,** c) 1:9 **6**:**5** (green), d) 1:9 **7**:**5** (purple), e) **5** (light blue).

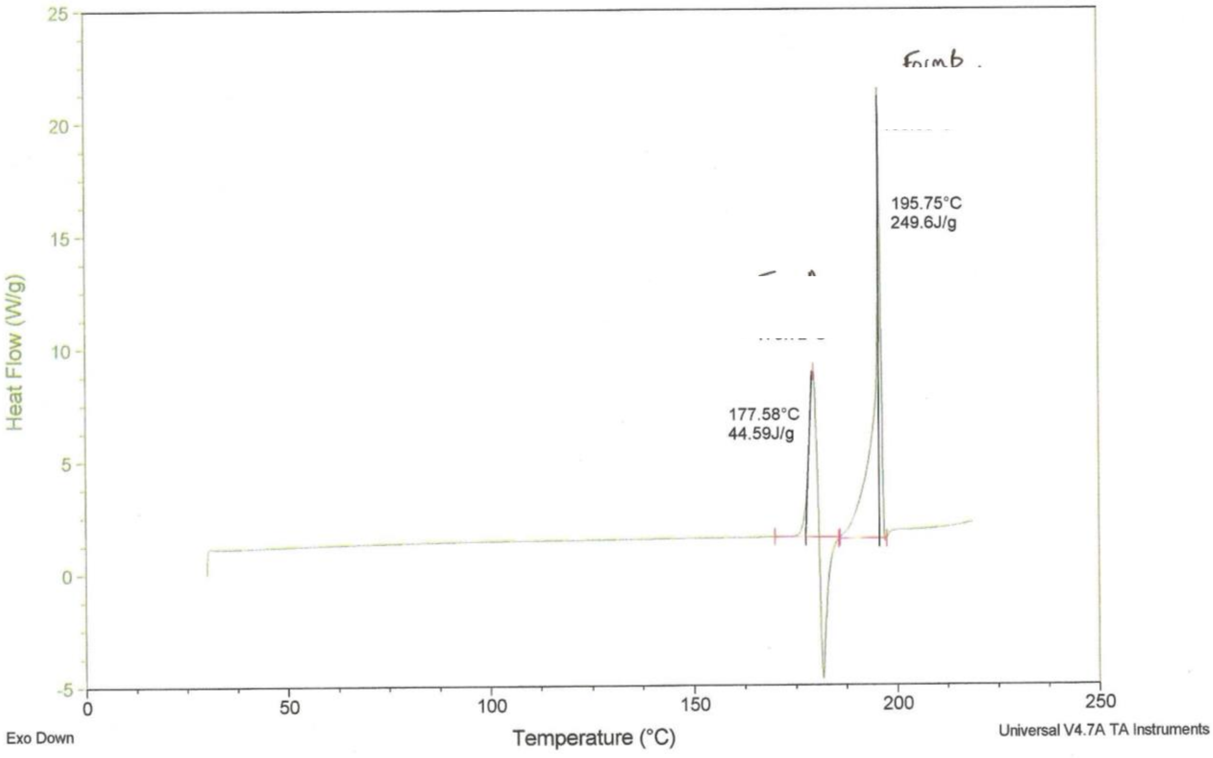

Figure S4.12 Differential Scanning Calorimetry (DSC) trace for partial gels formed by heating in toluene before complete dissolution. The trace shows heating of 0.8390 mg of **1** form A with at a rate of 10 °C/min.

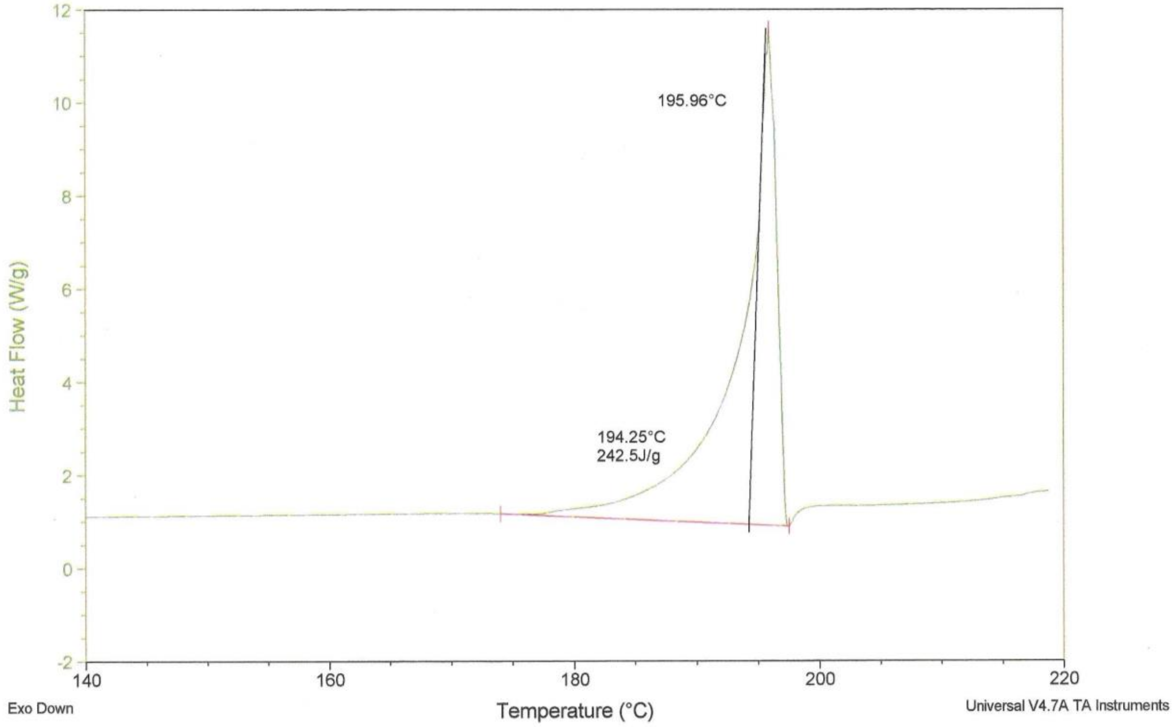

Figure S4.13 Differential Scanning Calorimetry (DSC) trace for dried toluene gels. The trace shows heating of 1.209 mg of **1** form A with at a rate of 10 °C/min.

S5 SEM studies

Samples prepared for SEM were applied directly to silicon wafer chips (Agar Scientific) using a cocktail stick for gels or pipettes for liquids and the solvent allowed to evaporate. Samples were stored under vacuum at $1x10^{-5}$ mbar then sputter coated with 5nm platinum in a Cressington 328 coating unit, at 40mA (density 21.09 and tooling set at 1) with rotation and a 30^{0} angle of tilt. Samples were imaged using a Hitachi S-5200 field emission scanning electron microscope at 1.5kV.

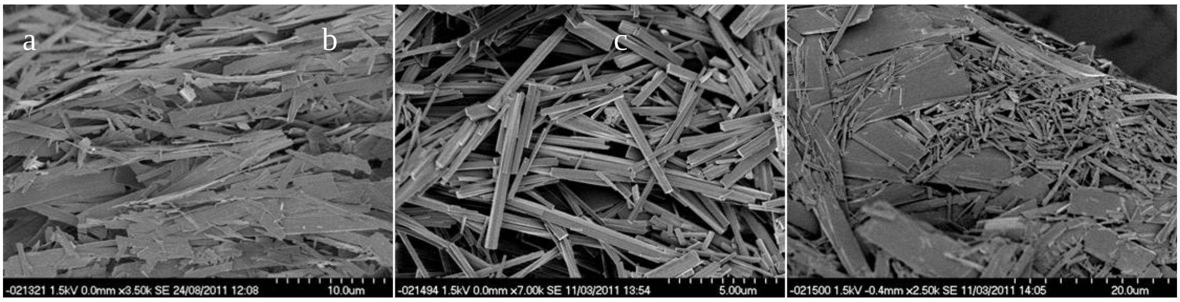

Figure S5.1: Xerogels formed from 1 % w/v gels of **1** from toluene assigned a) form A b) form B c) miture of form A and B

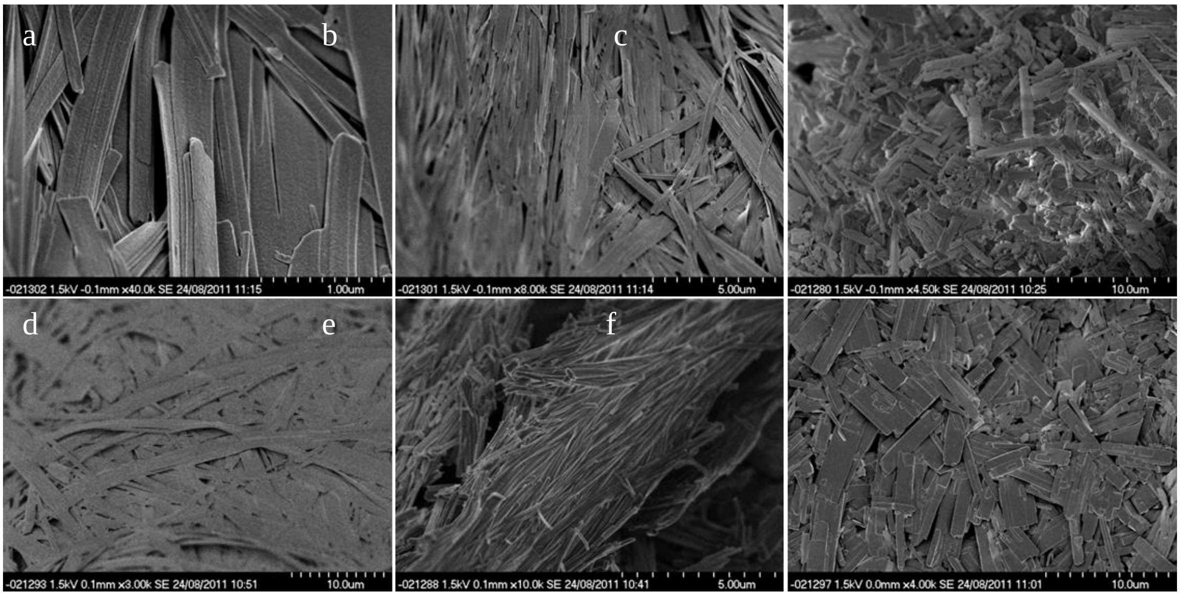

Figure S5.2: Xerogels formed from 1 % w/v gels of **2** in a) toluene, b) toluene, c) chloroform d) ethylacetate e) DCM, f) THF

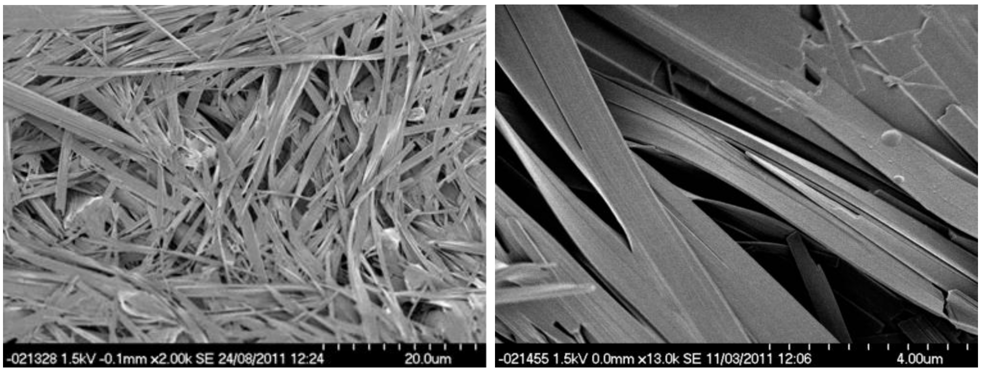

Figure S5.3: Xerogels formed from 1 % w/v toluene gels of **3**

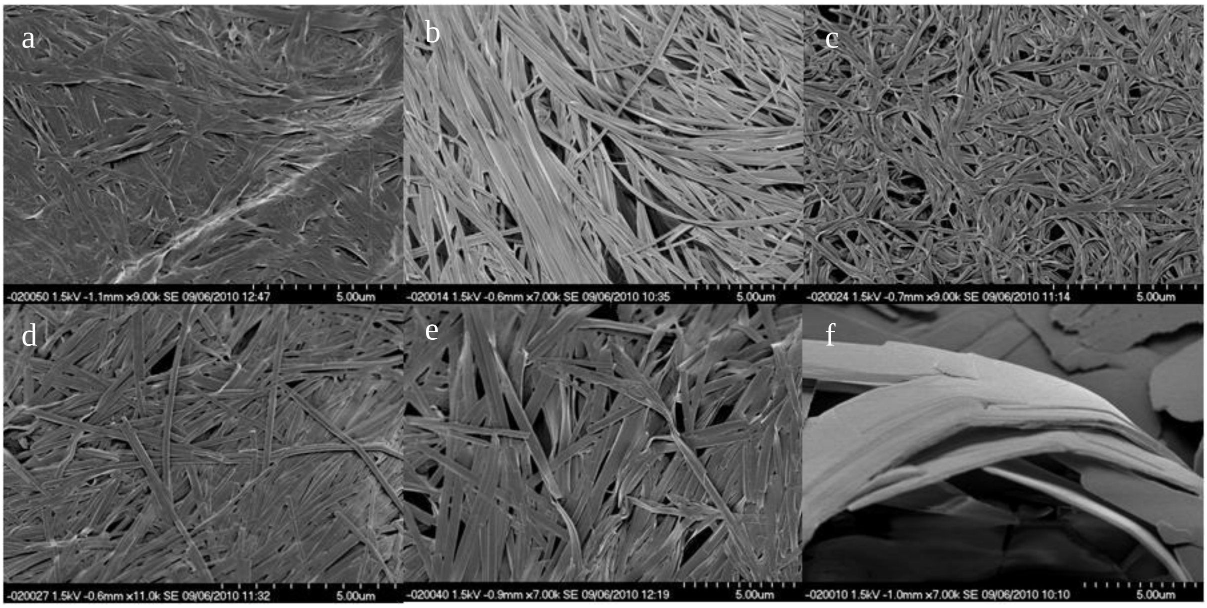

Figure S5.4 SEM images of 1 % w/v **4** fibres in a) toluene b) acetonitrile c) tetrahydrofuran d) chloroform e) ethyl acetate f) water.

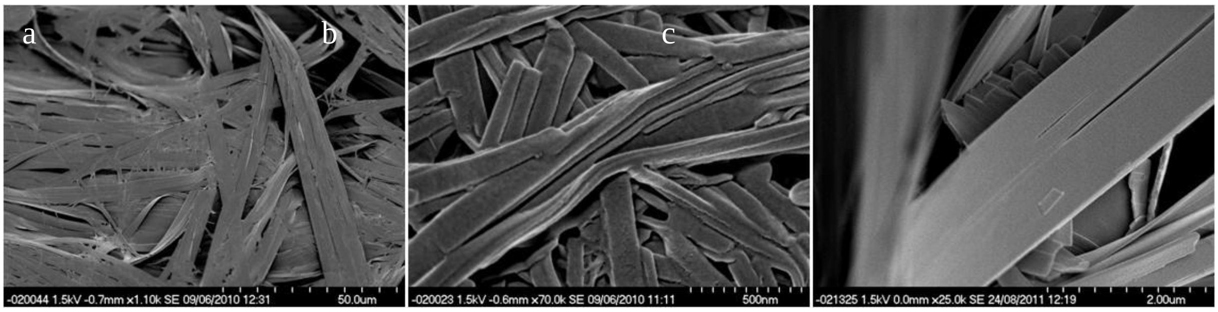

Figure S5.5 SEM images of xerogels formed from 1 % w/v gels of a) **4** in toluene, b) **4** in THF and c) **3** in toluene. Note the differences in scale between a) and b), stacking of ribbons in b) and splitting of ribbons in c).

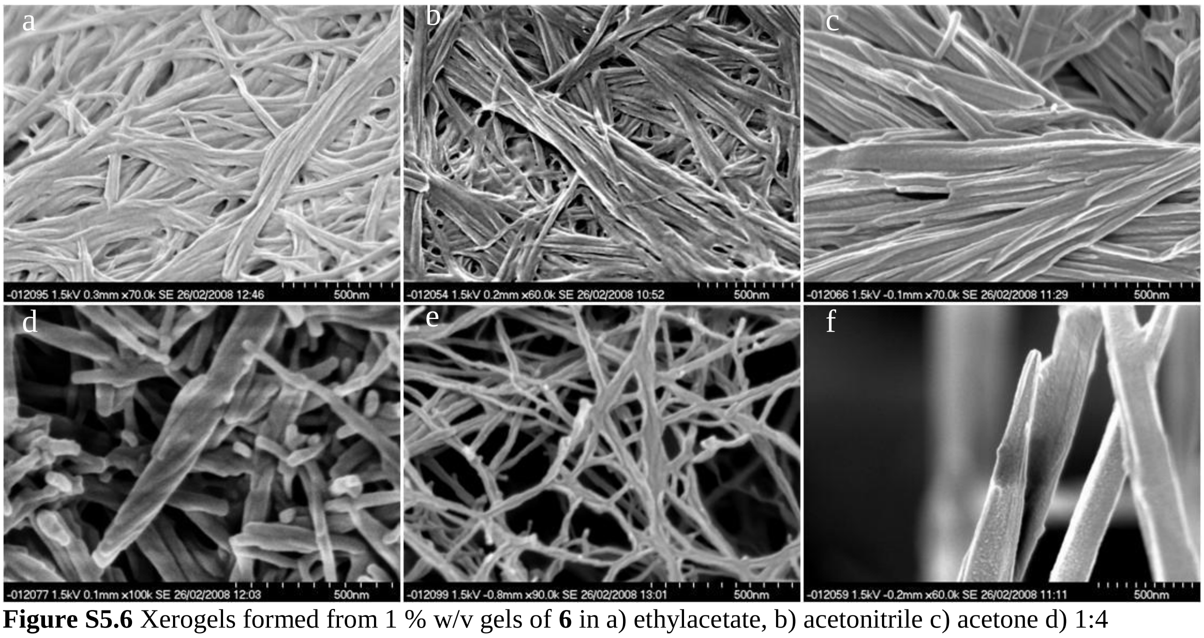

Figure S5.6 Xerogels formed from 1 % w/v gels of **6** in a) ethylacetate, b) acetonitrile c) acetone d) 1:4 THF:water, e) 1:1 DMSO water and f) 3:2 Methanol:water

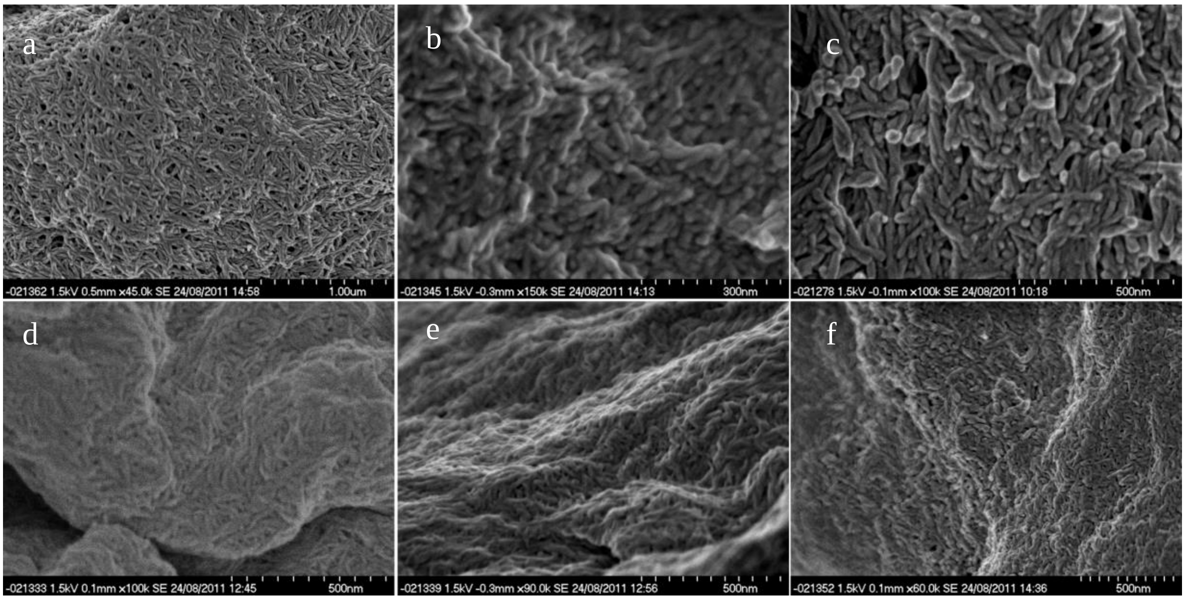

Figure 5.7: Xerogels formed from 1 % w/v gels of **7** in a) Toluene, b) ethylacetate c) acetone d) acetonitrile e) DCM f) THF

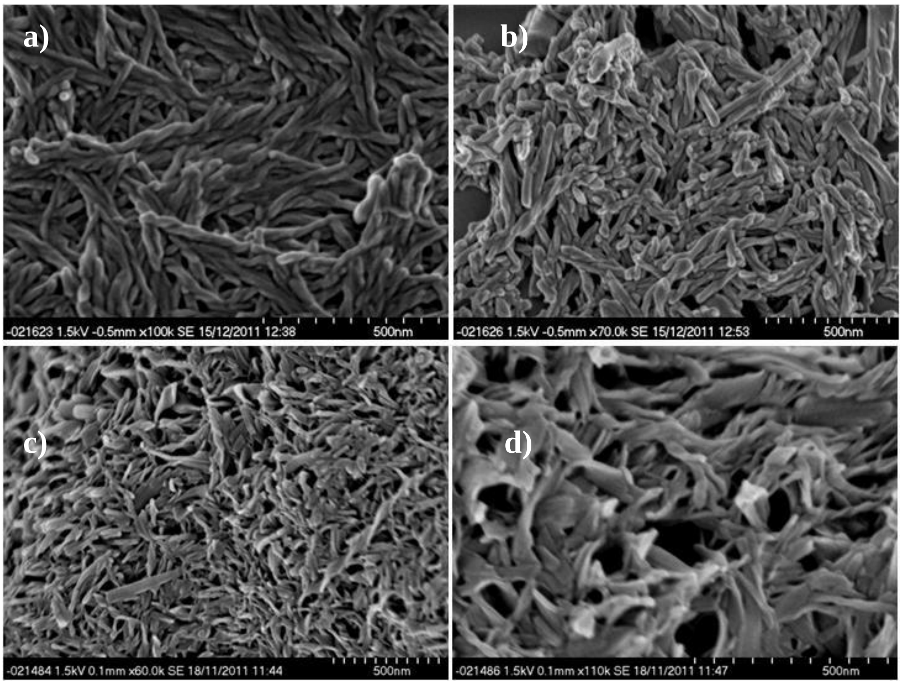

Figure 5.8 SEM images showing the morphology of 1 % w/v xerogels of 8 shown at various magnifications produced from: a) and b) toluene c) acetonitrile d) methanol

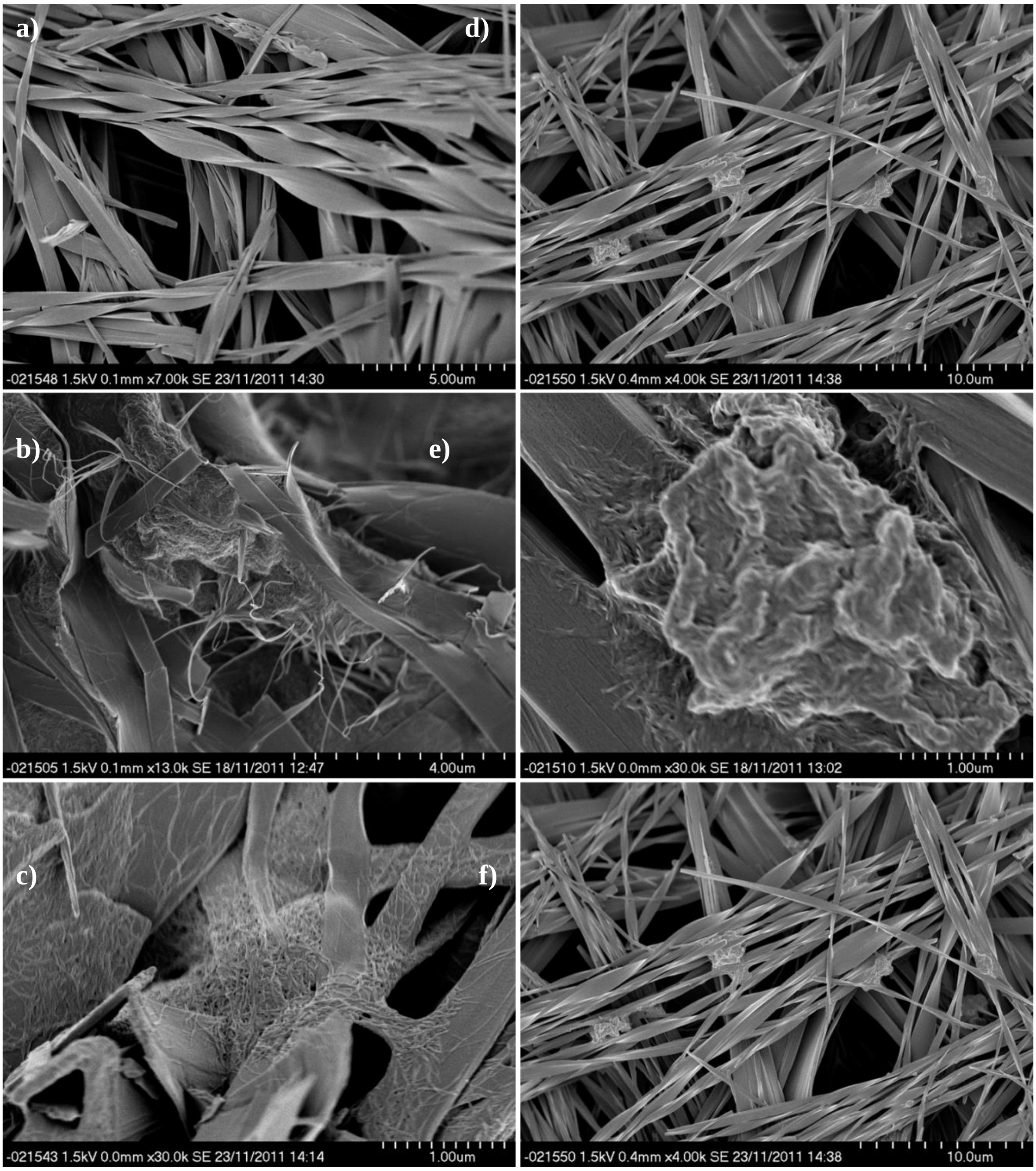

Figure S5.9 SEM images of xerogels formed from 1 % w/v 1:9 mixed toluene gels of a) and b) slow cooled **6**:**3**, c) fast cooled **6**:**3**, d) and e) slow cooled **6**:**3** f) fast cooled **6**:**3**

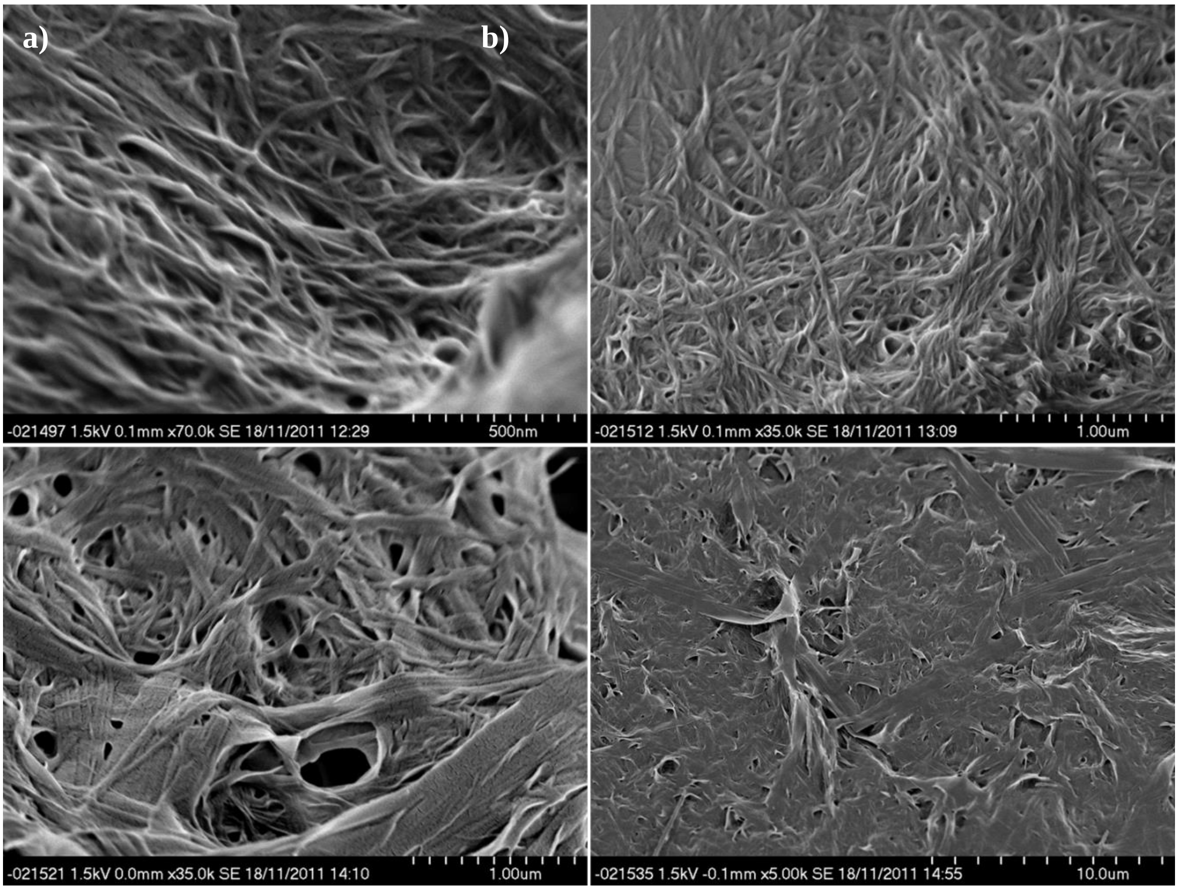

Figure S5.10 SEM images of xerogels formed 1 % w/v 1:9 mixed gels from a) slow cooled **6**:**4** b) slow cooled **7**:**4** c) fast cooled **6**:**4** d) fast cooled **7**:**4**.

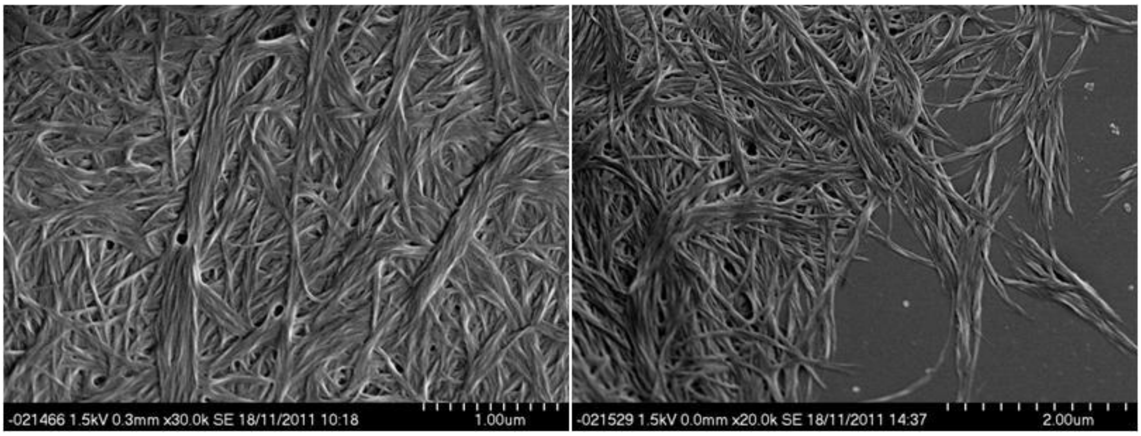

Figure S5.11 SEM images of xerogels formed from a total of 1 % w/v mixed gels in toluene with a ratio of a) 1:9 **6**:**5** slow cooled, b)1:9 **7**:**5** fast cooled.

S6 ^{1}H NMR spectroscopic titrations

NMR spectra were performed on a Varian Mercury-400 (400 MHz for ^{1}H). All chemical shifts are reported in ppm and referenced to the residual protic solvent. A solution of the host species of known concentration, typically 0.02–0.03 mM, was made up in an NMR tube using DMSO-d_6 (0.5 ml). Solutions of TBA Acetate (1 ml) were made ten times the concentration of the host solution. The guest solution was typically added in 10 µl aliquots, representing 0.2 equivalents of the guest with respect to the host. Larger aliquots were used above 2 equivalents of guest. Spectra were recorded after each addition. Results were analysed using the curve-fitting program HypNMR 2006.[6]

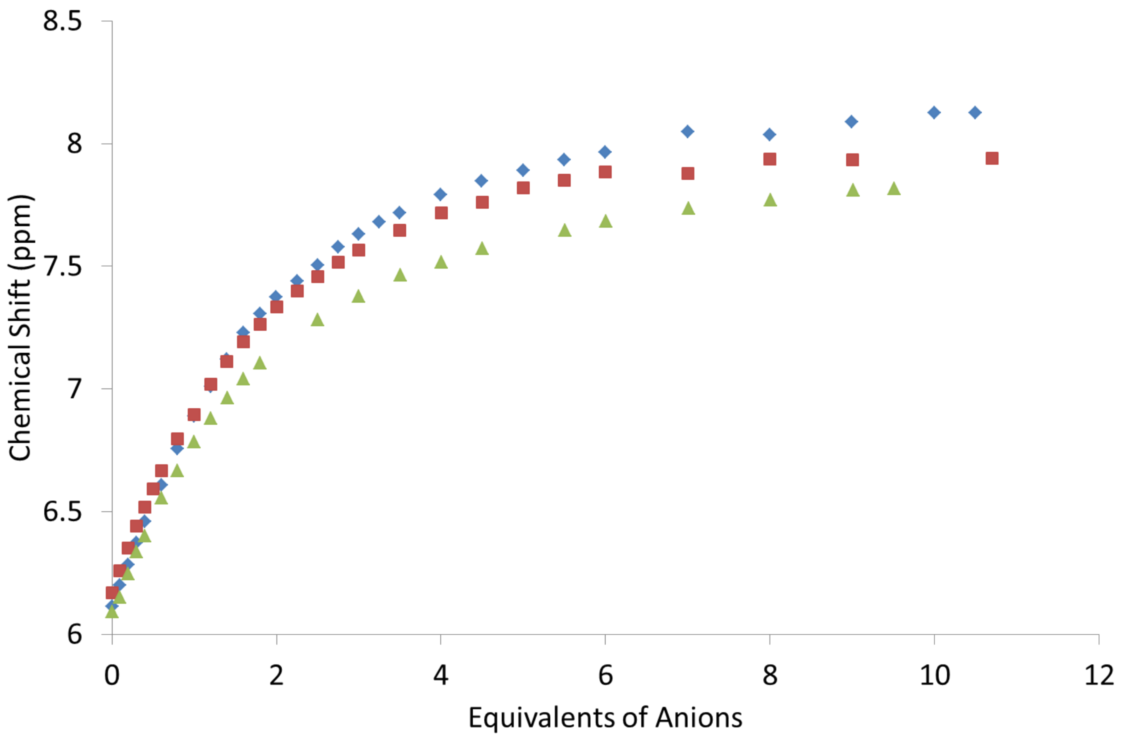

Figure S6.1 NMR titration data for TBA-acetate with gelators **3** (blue diamond), **4** (red square) and **5** (green triangle) in DMSO-d_6.

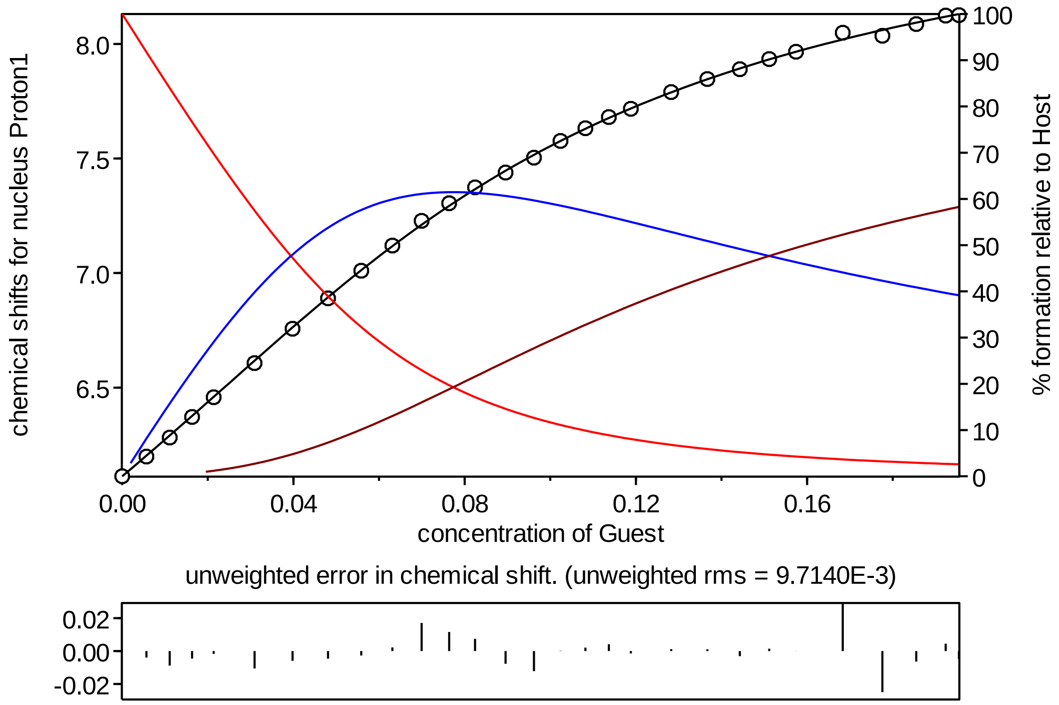

Figure S6.2 NMR titration data and fit for binding of TBA acetate by **3** in DMSO: black circles- experiemental measurements, black line- fit of model to data, red line- free host in solution, blue line- 1:1 host:guest binding, brown line- 1:2 host:guest binding.

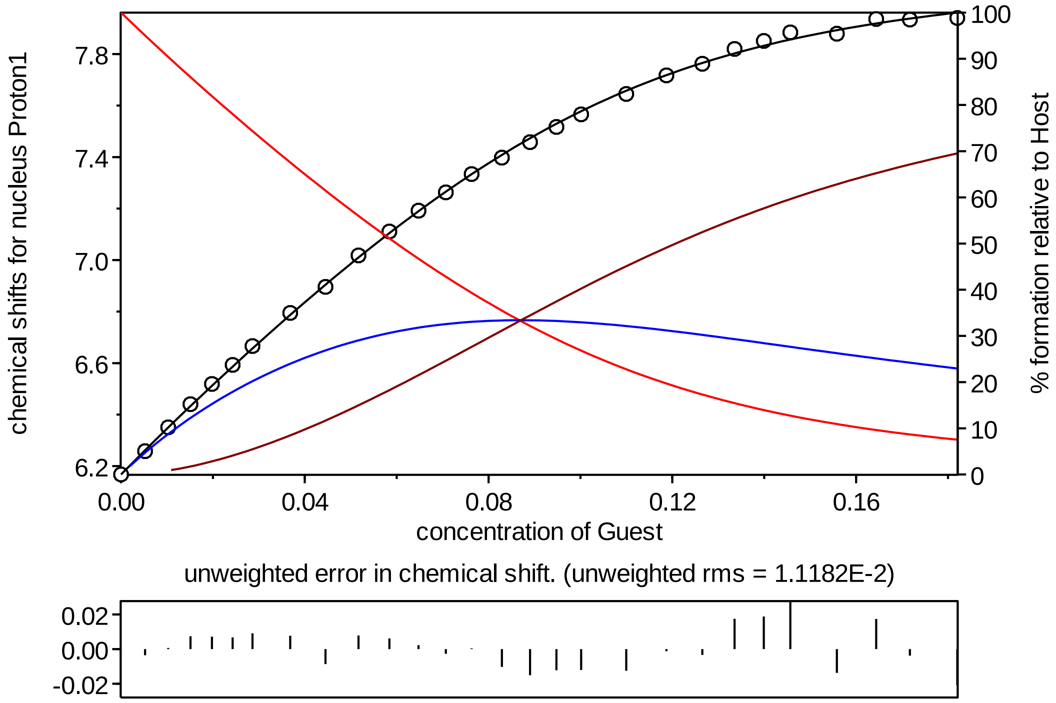

Figure S6.3 NMR titration data and fit for binding of TBA acetate by **4** in DMSO: black circles- experiemental measurements, black line- fit of model to data, red line- free host in solution, blue line- 1:1 host:guest binding, brown line- 1:2 host:guest binding.

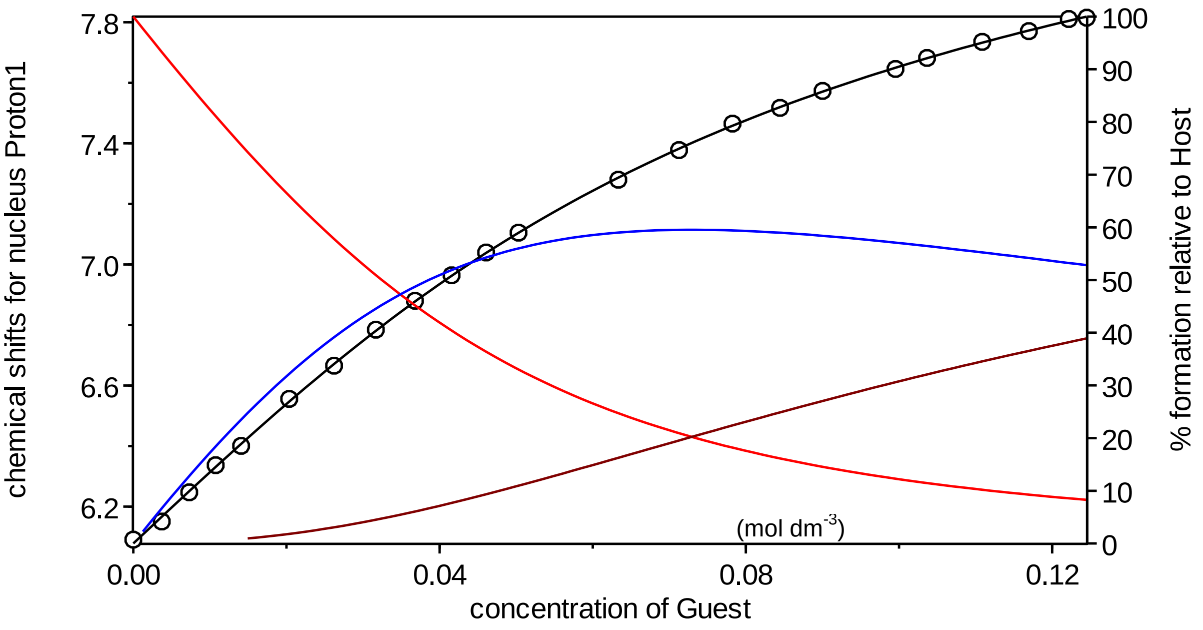

Figure S6.4 Speciation plot showing experimental data for ^{1}H NMR spectroscopy titrations for the binding of TBA acetate by **5** in DMSO fitted using a 1:1 and 1:2 host guest binding model: black circles- experimental measurements, black line- fit of model to data, red line- free host in solution, blue line- 1:1 host:guest binding, brown line- 1:2 host:guest binding.

S7 Job Plot

Stock solutions of **5** (0.0255 g in 5 ml DMSO-d_6) and TBA-acetate (0.0146 g in 5 ml DMSO) were prepared in glass vials. The solutions were added to separate NMR tubes to give samples with the same total volume (0.8 ml) but different mole fractions of **5**:TBA-acetate ranging from 0.9-0.1 **5**. Results were analysed in Microsoft Excel.

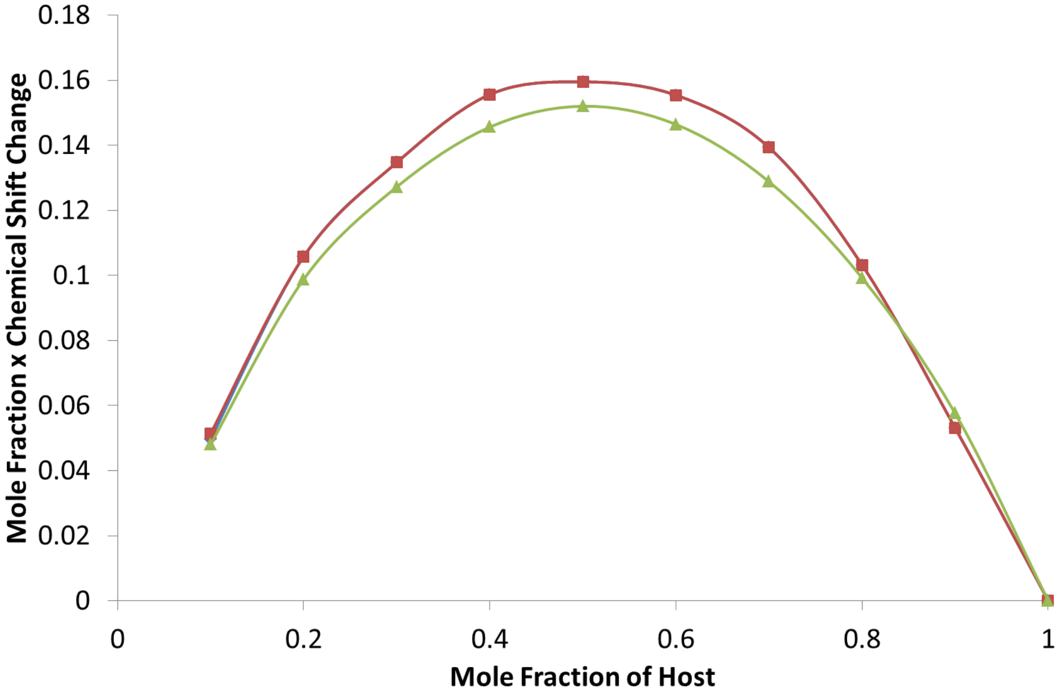

Figure S7.1 Job plot comparing the chemical shifts of the urea protons of **5** at different mole fractions of TBA-acetate. The maximum around 0.5 suggesting that the 1:1 species is predominant. The 2:1 complex is not observed in the Job plot as the weak binding means very little 2:1 complex formation takes place, particularly under the dilute concentrations used in the Job plot (about 2 mmol dm^{-3}).

S8 Rheological Studies

heology experiments were performed using a TA Instruments Advanced Rheometer 2000. Measurements of the gels were made using a 40 mm steel plate geometry was used with a gap of 500 µm and 4 ml of sample in each case. Samples were prepared by weighing 0.04 g of gelator into an 7 ml glass vial along with 4 ml of toluene (1 % w/v). The vials were sealed and heated until the gelator had fully dissolved (care must be taken due to pressure build up). The samples were rapidly cooled in a water bath and briefly sonicated at the first sign of precipitation to ensure homogeneous gel formation. The gel was transfered onto the centre of the plate of the rheometer using a spatula and the plate squashed the gel resulting in the loss of some solvent. Samples of **1** were prepared by combining four separate 1.7 ml vials each containing 1 ml of gel. Experiments were measured at 20°C and a solvent trap was used to reduce solvent evaporation. Frequency sweep measurements were performed over a range of 1 to 100 Hz with a constant osc. stress of 10 Pa. Oscillatory stress measurements were performed over a range of 0.01-100 % strain at a constant frequency of 1 Hz. Oscillatory stress sweep measurements were preformed over a range of 0.01-300 Pa with a constant frequency value of 1 Hz.

Studies on the effect of anion concentration on gel formation were conducted using a concentric cylinder couette geometry with a gap of 1000 µm and 10 ml of sample was used in each case. Samples were prepared by weighing 0.1 g of gelator into a 15 ml glass vial along with the appropriate molar equivalents of TBA-acetate and 10 ml of solvent mixture. The vials were sealed and carefully heated until the gelator had fully dissolved (care must be taken due to pressure build up). The hot gelator solution was transferred into the pre-heated concentric cylinders using a preheated glass pipette. Gels of **5** in 1:9 toluene to acetonitrile mixtures were cooled from 90-20°C whilst ethyl acetate gels of **5** and chloroform gels of **4** were cooled from 50-20°C. Oscillatory stress sweep measurements were preformed over a range of 0.01-300 Pa with a constant frequency value of 1 Hz.

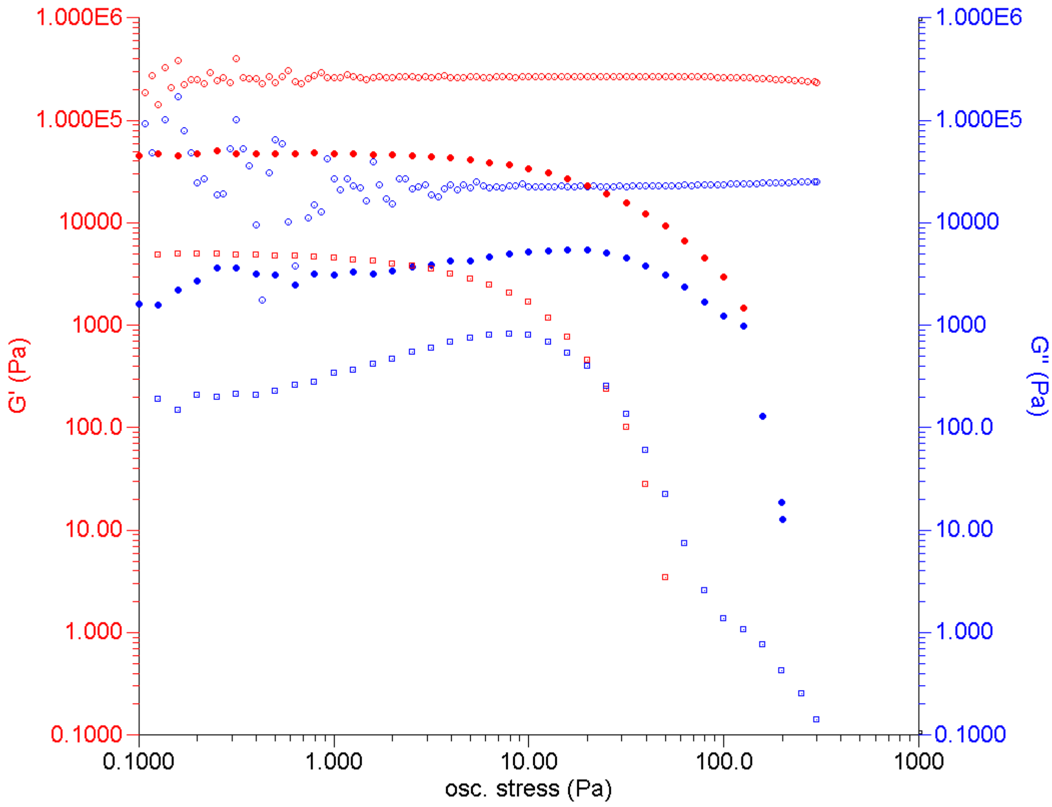

Figure S8.1 Oscillitory stress sweep comparing amino acid derived gelators at a constant frequency of 1 Hz.; open circles **5**, closed circles **4**, open squares **3**.

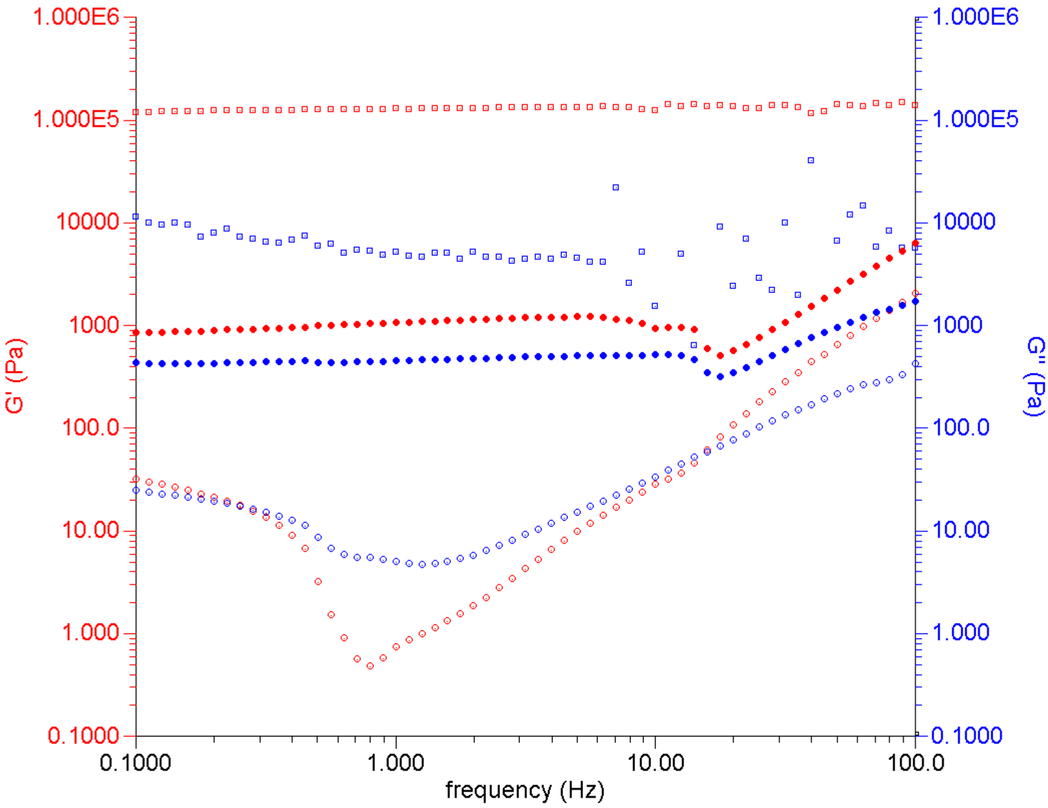

Figure S8.2 Frequency sweep comparing amino acid derived gelators at a constant stress of 10 Pa; 1 Hz.; open circles **1** form A, open squares **1** form B, closed circle **2**.

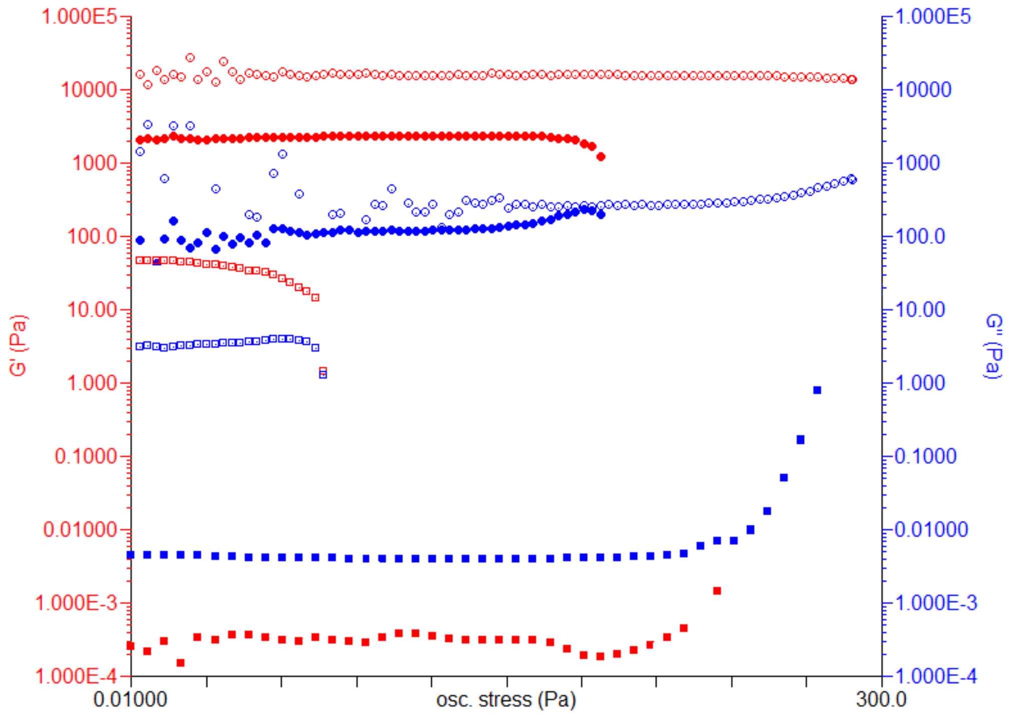

Figure S8.3 Oscillatory stress sweep for 1 % w/v **5** in 1:9 CH_3CN:toluene at increasing concentrations of TBA-acetate: 0 equiv. (open circle) 0.5 equiv. (closed circle) 1 equiv. (open squares) 1.5 equiv (closed square) at frequency of 1Hz. Data curtailed after yield stress for clarity.

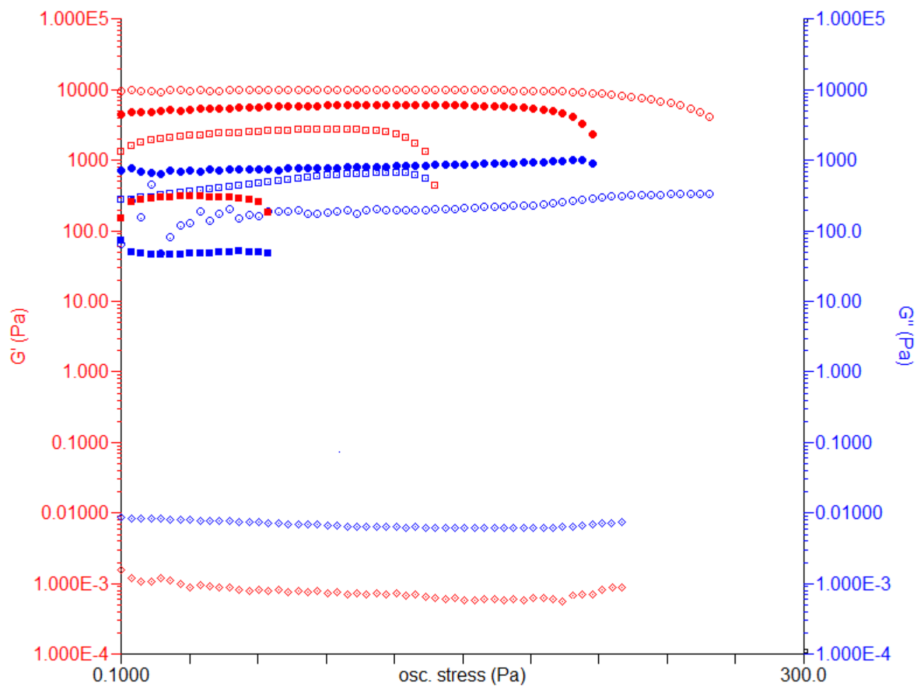

Figure S8.4 Plot of rheometric study showing *G′* (red) and *G″* (blue) values for 1% w/v ethyl acetate gels of **5** at various oscillatory stresses with varying equivalents of anions: 0 (open circles), 0.2 (closed circles), 0.4 (open squares), 0.6 (closed squares), 0.8 (open diamonds). Data curtailed after yield stress for clarity.

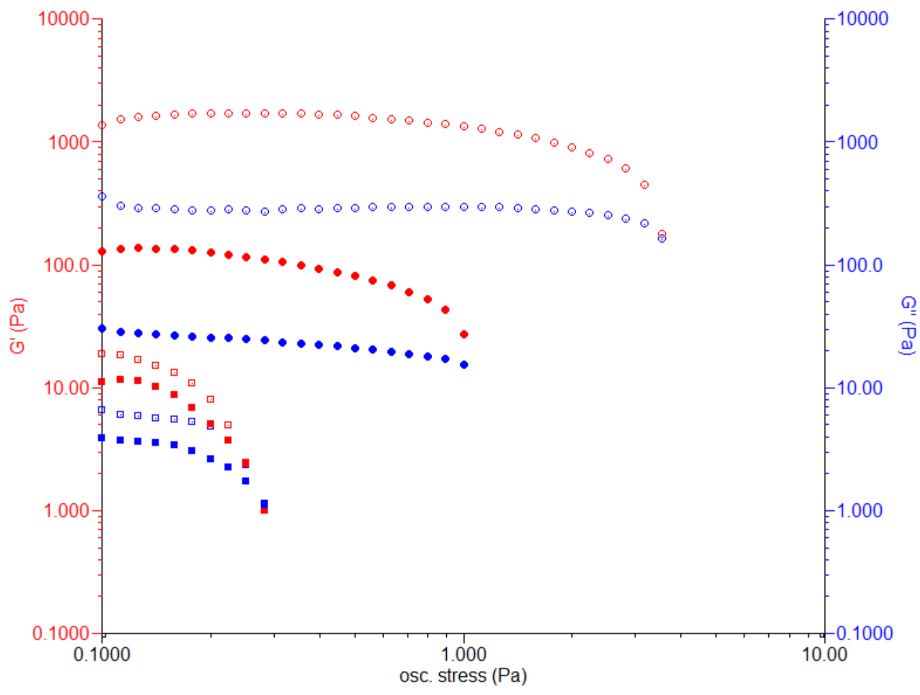

Figure S8.5 Plot of rheometric study showing *G′* (red) and *G″* (blue) values for 1% w/v chloroform gels of **4** at various oscillatory stresses with varying equivalents of anions; 0 (open circles), 0.1 (closed circles), 0.2 (open squares), 0.3 (closed squares), 0.4 (open diamonds), 0.5 (closed diamonds). Data curtailed after yield stress for clarity.

S9 Fluorescence Studies

UV-vis absorption spectra were obtained on a Hewlett-Packard 8453 diode array spectrophotometer using standard 1 cm width quartz cells. All emission spectra were produced with an excitation wavelength of 345 nm which corresponds to a maximum in the UV-vis absorption spectra for both **6** and **7** in dilute toluene solution. Emission spectra were obtained using a Jobin-Yvon Horiba Fluorolog 3-22 Tau-3 spectrofluorimeter and were corrected for the spectral response of the instrument. Solution based spectra were recorded with a right angle illumination and collection method using a standard quartz cell with a path length of 1 cm. Gel samples were measured in front-facing geometry in 1.75 ml screw top vial. Slit widths were varied with samples ranging from 1.5 to 8 nm depending on concentration and intensity of sample emission.

Analytical grade solvents were used without drying or degassing procedures. No emission is observed for gelators **3** or **4** and negligibly weak fluorescence was observed for concentrated gels of phenylalanine derived gelator **5**. Dilute toluene solutions were produced by heating and sonicating 1 mg of **6** or **7** in 10 ml of solvent and to create a saturated solution at room temperature. The samples were then filtered through cotton wool to remove excess solid and the concentration was estimated to be in the order of 1 x 10^{-7} mol dm^{-3}.

1:9 mixed gel samples were prepared by weighing 0.0010 g of **6** or **7** and 0.0090 g of **3**, **4** or **5** into glass screw top vials and adding 1 ml of solvent. Samples were repeatedly heated with a heat gun and sonicated to ensure complete dissolution and homogeneous gel formation. Slow cooled samples were prepared by placing the vial on a wooden bench top and allowing to cool to room temperature. Fast cooled samples were prepared by heating the sample with a heat gun until fully dissolved. The sample was then rapidly immersed in a water bath (16°C) and at the first sign of phase separation (approximately 10 seconds) briefly sonicated (approximately 1 second). If the sample is sonicated well above the T_{gel} the sample remains in solution whilst prolonged sonication disrupts gels which have already formed. Differences in the temperature to which samples are heated and the stochastic nature of gel nucleation can mean there is some variation in the time taken for gels to form.

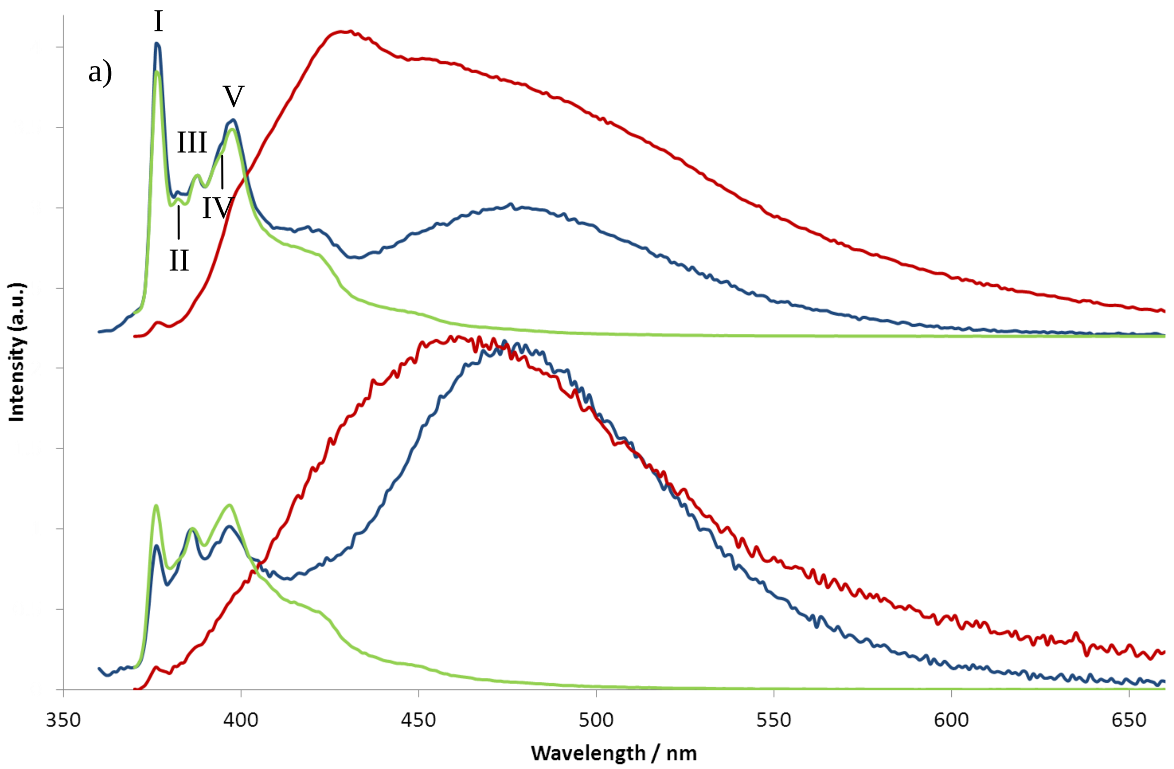

Figure 9.11 Overlaid normalised emission spectra for (a) (*S*)-Methyl-2-(*tert*-Butoxycarbonylamino)-3-(pyren-1-yl)propanoate in dilute toluene solution (green), 1-pyrenylalanine gelator **6** in dilute solution (blue) and **6** as a 1 % w/v gel. Bands I-V are labelled. (b) (*S*)-Methyl-2-(*tert*-Butoxycarbonylamino)-3-(pyren-2-yl)propanoate in dilute toluene solution (green), 2-pyrenylalanine gelator **7** in dilute solution (blue) and **7** as a 1 % w/v precipitate. λ_{ex} = 345 nm, spectra normalised to band III (386 nm) apart from the 1 % w/v samples of **6** and **7** which are adjusted to allow comparison between the spectra.

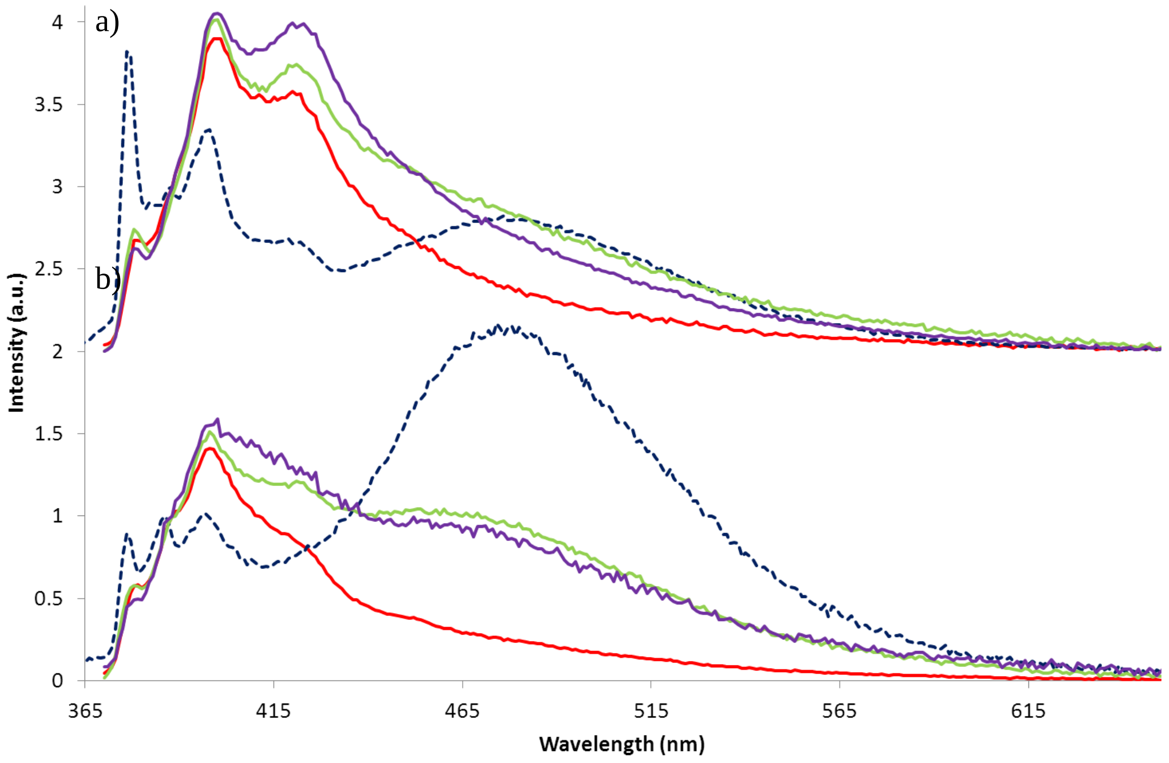

Figure S9.2 Overlaid normalised spectra for (a) **6** (b) **7** in 1:9 mixed gels with **3** (green), **4** (purple) **5** (red) and as a dilute toluene solution (dotted blue). λ_{ex} = 345 nm, spectra normalised to band III (386 nm).

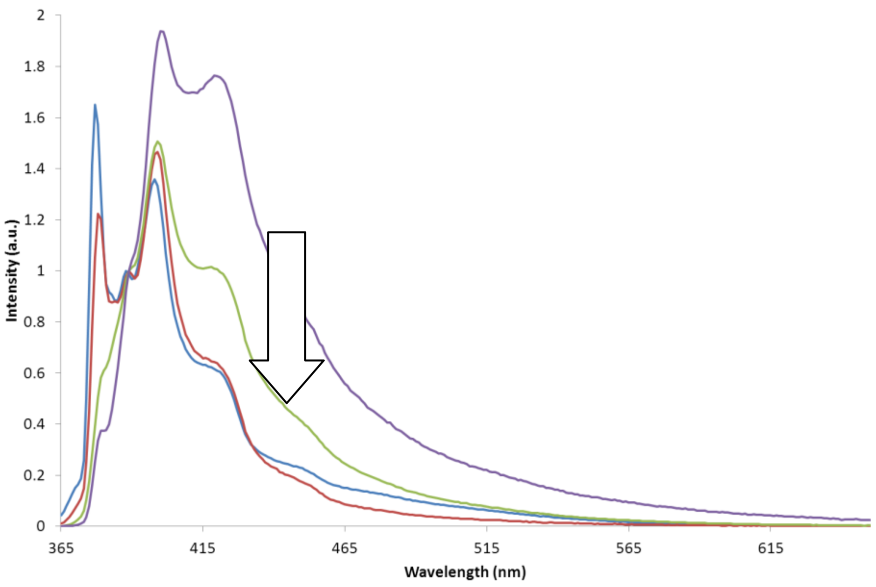

Figure S9.3 Mixed 1 % w/v toluene gels with of **6**:**5** at different ratios: 1:9 (purple), 1:19 (green), 1:99 (red), saturated solution of **6** in 1 % w/v gel of **5** (blue). Arrow shows effect of decreasing ratio of **6** relative to **5** on the intensity of the excimer band. λ_{ex} = 345 nm, spectra normalised to band III (386 nm).

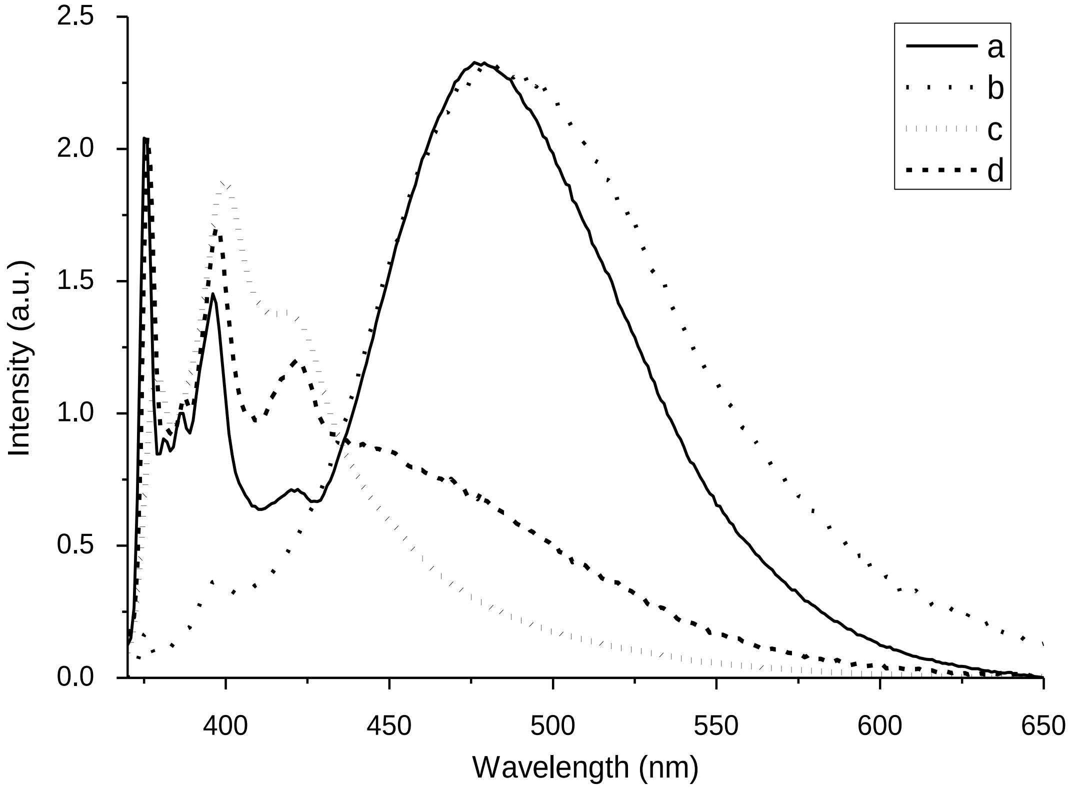

Figure S9.4 Fluorescence emission spectra of **6** (λ_{ex} =345 nm) (a) in dilute toluene solution (~1x10^{-7} mol dm^{-3}), (b) as a single-component toluene gel, (c) co-gel blend of **5** and **6** (9:1) and (d) after treatment of the **5**:**6** blend with 10 molar equivalents of NBu_4OAc. The spectra are normalized at 386 nm apart from (b) which is scaled to match the intensity of the excimer peak of (a).

Sample preparation was found to have a significant influence on the emission spectra of the resulting mixed gels. All mixed gel samples were heated with a heat gun until a clear solution was formed. Two different cooling protocols were then adopted: a fast cooling method in which the samples were imersed in a cool water bath at approximately 16°C and briefly sonicated for approximately 1 s, and a slow cooling method where the samples were simply placed on a wooden bench top and allowed to cool. Fast cooled samples generally formed gels in under a minute whilst slow cooled samples took up to 10 minutes for the gels to fully form. Repeats were carried out using the same samples with the gel reformed each time according to either the fast or slow cooling procedures. Heating of the samples took place in sealed vials as the temperature required to dissolve gelators **3** and **4** is greater than that of the boiling point of the solvent (toluene 110°C). It was found that a heat gun, rather than a thermostatically controlled heating block, allowed the gelator to be rapidly dissolved without damaging the plastic caps of the vials. Consequently, the temperature to which each sample is heated will differ slightly for each repeat which may explain the variation seen within the results from each protocol. The resulting emission spectra are shown in Figure S9.5 with fast cooled samples shown in blue and slow cooled samples in red. Some differences were found in the morphology and XRPD patterns of samples from cold and fast cooled samples, however, no theory to account for the changes could be developed and they are not discussed further here.

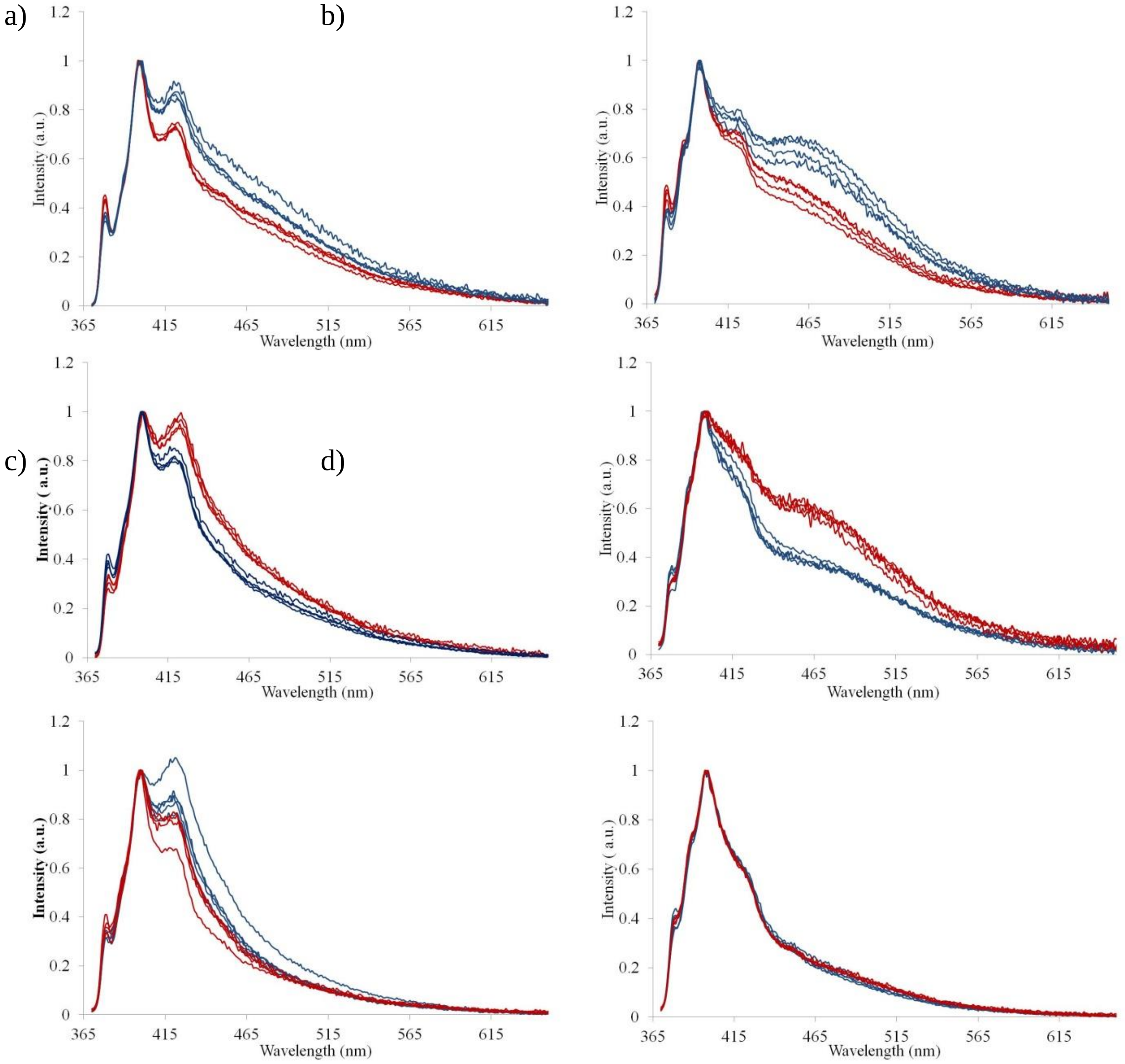

Figure S9.5 Overlaid fluorescence emissions spectra for fast (blue) and slow (red) cooled 1 % w/v 1:9 toluene gels of: a) **6**:**3** b) **7**:**3** c) **6**: **4** d) **7**:**4** e) **6**:**5** f) **7**:**5**. λ_{ex} = 345 nm, spectra normalised to band V (399 nm).

Table S9.1 Phase of mixed gels upon the addition of increasing equivalents of TBA-acetate.

Mixed Gel 1:9		Anion	TBA-Acetate solution							
			0	0.5	1	1.5	2	5	10	20
**6**	**3**	AcO^-	G	G	WG	S	S	S	S	-
		BF_4	G	G	G	G	G	PG	PG	PG
	**4**	AcO^-	G	G	WG	S	S	S	S	-
		BF_4	G	G	G	G	G	G	G	WG
	**5**	AcO^-	G	G	WG	S	S	S	S	-
		BF_4	G	G	G	G	G	G	G	WG
**7**	**3**	AcO^-	G	G	WG	S	S	S	S	S
		BF_4	G	G	G	G	G	PG	PG	PG
	**4**	AcO^-	G	G	WG	S	S	S	S	S
		BF_4	G	G	G	G	G	G	G	WG
	**5**	AcO^-	G	G	WG	S	S	S	S	S
		BF_4	G	G	G	G	G	G	G	WG

G= gel, WG= weak gel, PG= partial gel, S= solution.

S10 Anion addition to 6 and 7 in dilute DMF solution

Concentrated stock solutions were prepared by dissolving **6** or **7** (0.001 g) in DMF (1 ml). Samples were made by diluting 0.02 ml of stock solution with 2 ml of DMF in a quartz cuvette. Stock solutions of anion were formed by dissolving TBA actetate (0.0390 g) or TBA tetrafluoroborate (0.0425 g) in 1 ml of DMF. Anion solutions were added to the cuvette using a microsyringe and shaken, but not heated, between measurements.

a) b)

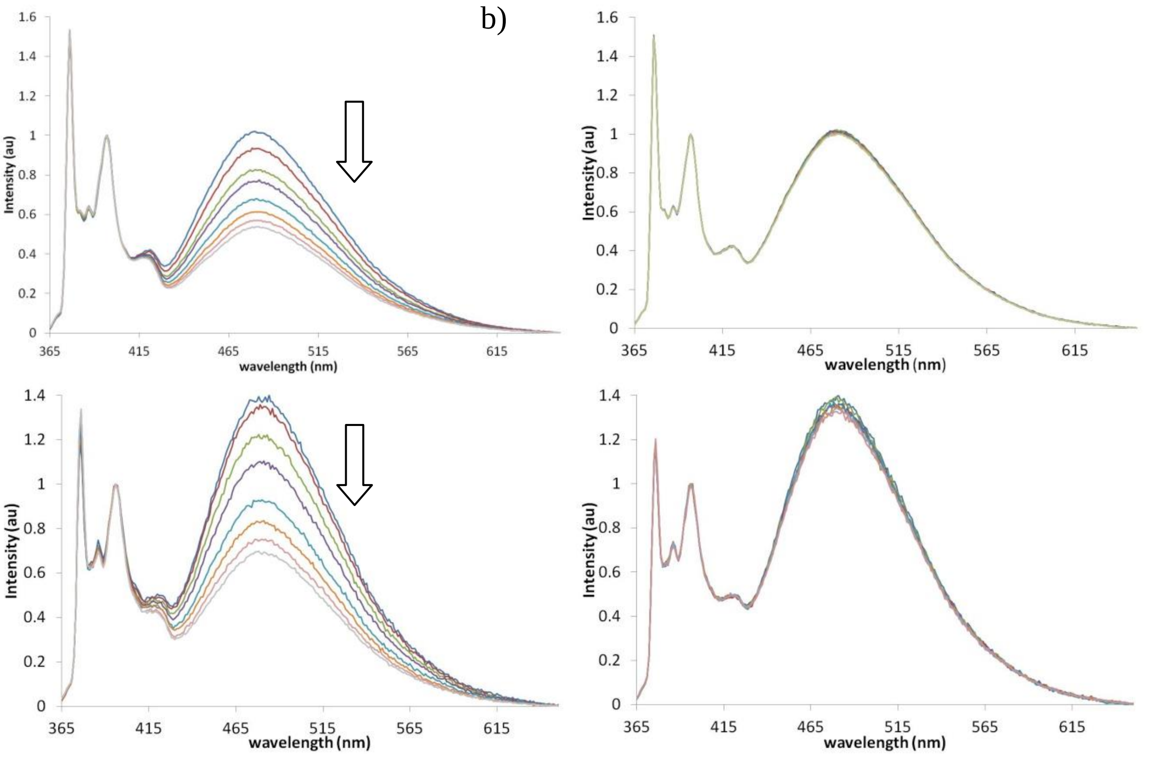

Figure S10.1 Emission spectra for a solution of (a and b) **6** or (c and d) **7** in DMF (1.3 x10^{-5} mol dm^{-3}) to which increasing equivalents of (a and c) TBA-OAc or (b and d) TBA-BF_4 solution (1.3 x10^{-4} mol dm^{-3}) are added: 0 eq. (blue), 50 eq. (red) 250 eq. (green) 500 eq. (purple) 1000 eq. (light blue) 1500 eq (orange) 2000 eq. (pink) 2500 eq. (grey). Arrow shows effect of the addition of increasing equivalents of anions on excimer band. λ_{ex} = 345 nm, spectra normalised to band V (398 nm).

S11 Anion addition to 6 and 7 in toluene mixed gels

Stock solutions were prepared by heating and sonicating 1 mg of **6** o **7** in 10 ml of toluene for 5 minutes then allowing the sample to cool to room temperature. Saturated solutions were formed by passing the solution through a pipette containing cotton wool to remove the undissolved material. Mixed gel samples were prepared by weighing 10 mg of gelators **3**, **4** and **5** into 1.75 ml vials and adding 1 ml of the toluene stock solution containing **6** or **7**. The samples were heated until all material was fully dissolved then rapidly cooled in a water bath with brief sonication in accordance with the fast cooling method. Stock solutions of anion were prepared by dissolving 0.172 g of TBA-acetate or 0.187 g of TBA-tetrafluoroborate in 0.6 ml of acetonitrile. Gels were reformed following the addition of each aliquot of anions and emission spectra recorded after a 5 minute cooling period.

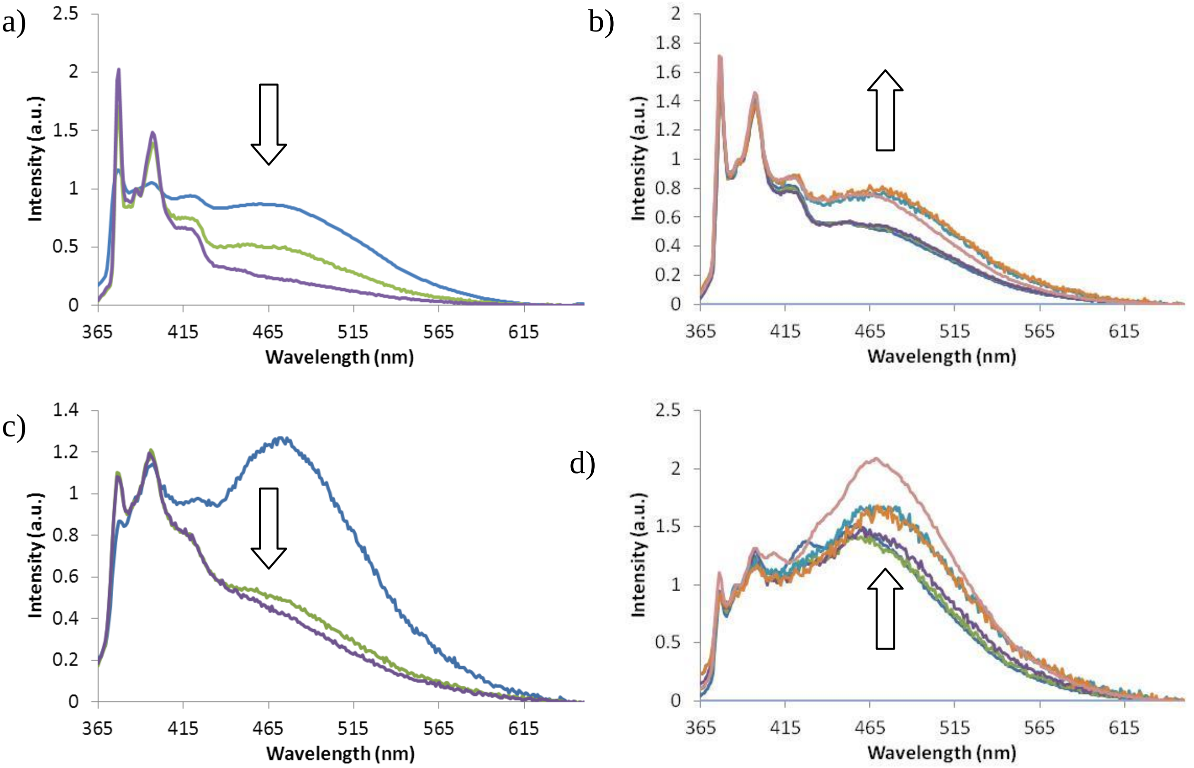

Figure S11.1 Fluorescence emission spectra recorded for saturated toluene solutions of (a and b) **6** (c and d) **7** upon the addition of (a and c) TBA-Acetate solution or (b and d) TBA-tetrafluoroborate solution (0.95 mmol dm^{-3}): 0 ml (blue), 0.02 ml (green), 0.04 ml (purple). Further additions of BF_4 solution were made: 0.01 ml (turquoise), 0.1 ml (orange), 0.2 ml (light blue), 0.4 ml (pink). Arrows show the effect of increasing concentration of anion solution on excimer band intensity. λex = 345 nm, spectra normalised to band III (386 nm).

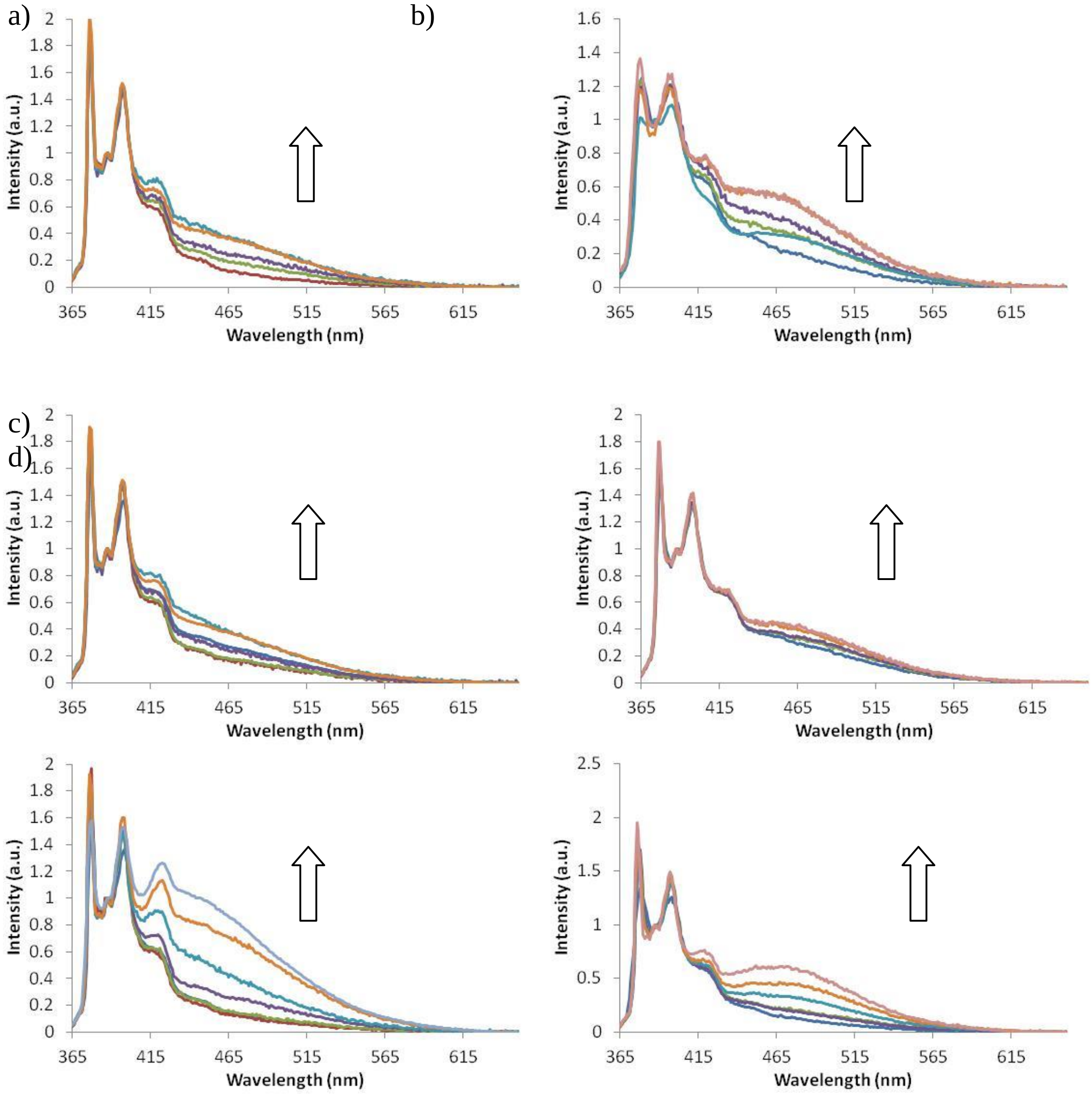

Figure S11.2 Emission spectra showing the addition of anion solutions to 1 % w/v 1:9 mixed gels of **6** with a) **3**+AcO^{-}, b) **3**+BF_4^-, c) **4**+AcO^{-} , d) **4**+BF_4^-, e) **5**+AcO^{-}, f) **5**+BF_4^-: 0 eq. (blue), 0.5 eq (red), 1 eq. (green), 2 eq. (purple), 5 eq. (turquoise), 10 eq (orange), 15 ml (light grey), 20 eq (pink). Arrows shows the effect of increasing concentration of anion solution on the excimer band. λ_{ex} = 345 nm, spectra normalised to band III (386 nm).

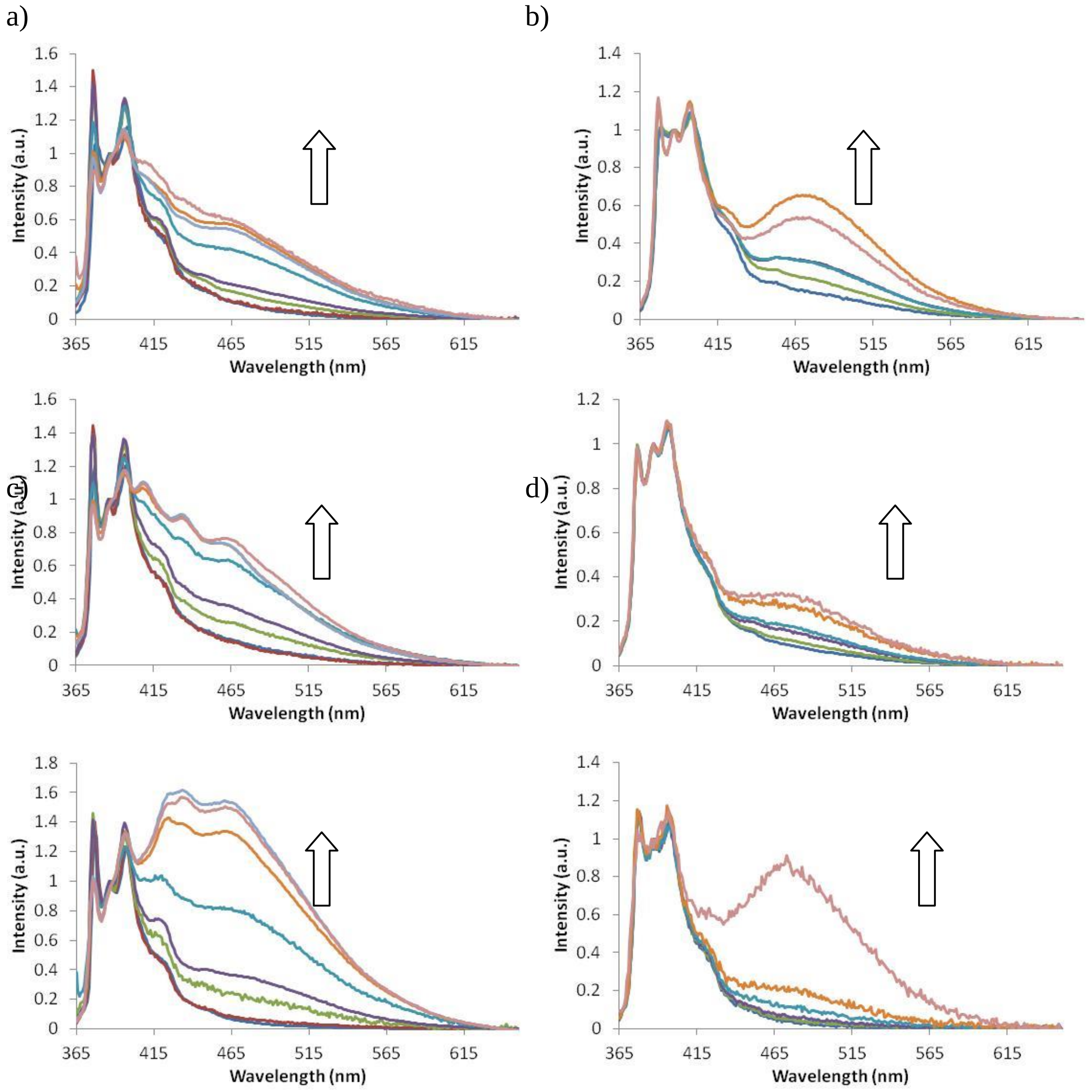

Figure S11.3 Emission spectra showing the addition of anion solutions to 1 % w/v 1:9 mixed gels of **7** with a) **3**+AcO^-, b) **3**+BF_4^-, c) **4**+AcO^- , d) **4**+BF_4^-, e) **5**+AcO^-, f) **5**+BF_4^-: 0 eq. (blue), 0.5 eq (red), 1 eq. (green), 2 eq. (purple), 5 eq. (turquoise), 10 eq (orange), 15 ml (light grey), 20 eq (pink). Arrows shows the effect of increasing concentration of anion solution on the excimer band. λ_{ex} = 345 nm, spectra normalised to band III (386 nm).

References

[1] B. M. Trost and M. T. Rudd, *Org. Lett.* **2003**, *5*, 4599-4602.

[2] G. M. Sheldrick, *Acta Crystallogr. Sect. A* **2008**, *64*, 112-122.

[3] O. V. Dolomanov, L. J. Bourhis, R. J. Gildea, J. A. K. Howard and H. Puschmann, *J. Appl. Crystallogr.* **2009**, *42*, 339-341.

[4] L. J. Barbour, *J. Supramol. Chem.* **2001**, *1*, 189-191.

[5] G. Cravotto and P. Cintas, *Chem. Soc. Rev.* **2009**, *38*, 2684-2697.

[6] P. Gans in *HypNMR 2006*, *Vol.* University of Leeds, Leeds, **2006**.
